# Heuristic algorithms for feature selection under Bayesian models with block-diagonal covariance structure

**DOI:** 10.1186/s12859-018-2059-8

**Published:** 2018-03-21

**Authors:** Ali Foroughi pour, Lori A. Dalton

**Affiliations:** 1 Department of Electrical and Computer Engineering, The Ohio State University, 2015 Neil Avenue, Columbus, Ohio, 43210 USA; 2 Department of Biomedical Informatics, The Ohio State University, 250 Lincoln Tower, 1800 Cannon Drive, Columbus, Ohio, 43210 USA

**Keywords:** Feature selection, Bayesian learning, Biomarker discovery, Heuristic search algorithms

## Abstract

**Background:**

Many bioinformatics studies aim to identify markers, or features, that can be used to discriminate between distinct groups. In problems where strong individual markers are not available, or where interactions between gene products are of primary interest, it may be necessary to consider combinations of features as a marker family. To this end, recent work proposes a hierarchical Bayesian framework for feature selection that places a prior on the set of features we wish to select and on the label-conditioned feature distribution. While an analytical posterior under Gaussian models with block covariance structures is available, the optimal feature selection algorithm for this model remains intractable since it requires evaluating the posterior over the space of all possible covariance block structures and feature-block assignments. To address this computational barrier, in prior work we proposed a simple suboptimal algorithm, 2MNC-Robust, with robust performance across the space of block structures. Here, we present three new heuristic feature selection algorithms.

**Results:**

The proposed algorithms outperform 2MNC-Robust and many other popular feature selection algorithms on synthetic data. In addition, enrichment analysis on real breast cancer, colon cancer, and Leukemia data indicates they also output many of the genes and pathways linked to the cancers under study.

**Conclusions:**

Bayesian feature selection is a promising framework for small-sample high-dimensional data, in particular biomarker discovery applications. When applied to cancer data these algorithms outputted many genes already shown to be involved in cancer as well as potentially new biomarkers. Furthermore, one of the proposed algorithms, SPM, outputs blocks of heavily correlated genes, particularly useful for studying gene interactions and gene networks.

**Electronic supplementary material:**

The online version of this article (10.1186/s12859-018-2059-8) contains supplementary material, which is available to authorized users.

## Background

Many bioinformatics studies aim to identify predictive biomarkers that can be used to establish diagnosis or prognosis, or to predict a drug response [1–3]. This problem can often be framed as a feature selection task, where the goal is to identify a list of features (molecular biomarkers) that can discriminate between groups of interest based on high-dimensional data from microarray, RNA-seq, or other high-throughput technologies.

Initially, exploratory studies are often conducted on small samples to generate a shortlist of biomarker candidates before a large-sample validation study is performed [4]. However, such studies have too often been unsuccessful at producing reliable and reproducible biomarkers [5]. Biomarker discovery is inherently difficult, given the large number of features, highly complex interactions between genes and gene products, enormous variety of dysfunctions that can occur, and many sources of error in the data. As a result, feature selection algorithms are often implemented without much consideration of the particular demands of the problem. For instance, variants of t-test are perhaps the most widely implemented selection strategies in bioinformatics, but can only detect strong individual features, and fail to take correlations into account.

Given that molecular signaling is often inherently multivariate, there is a need for methods that can account for correlations and extract combinations of features as a marker family. Wrapper methods do this by ranking sets of features according to some objective function, usually the error of a classifier. However, methods based on classifier error are computationally expensive, and may not necessarily produce the best markers; indeed, strong features can be excluded if they are correlated with other strong features. Furthermore, analysis downstream from feature selection may include gene set enrichment analysis, where the hope is to identify known pathways or other biological mechanisms that contain a statistically significant number of genes in the reported gene set, or may involve the development of new pathways and gene networks. We are thus motivated to develop methods that not only select markers useful for discrimination, but select all relevant markers, even individually weak ones.

To address this, in prior work we proposed a hierarchical Bayesian framework for feature selection, labeling features as “good” or “bad”, where good features are those we wish to select, i.e., biomarkers. This framework places a prior on the set of good features and the underlying distribution parameters. Three Gaussian models have been considered. Under independent features, Optimal Bayesian Filtering reports a feature set of a given size with a maximal expected number of truly good features (CMNC-OBF) [6]. Assuming fully dependent good features and independent bad features, 2MNC-DGIB is a fast suboptimal method that ranks features by evaluating all sets of size 2 [7]. Finally, assuming good and bad features are separately dependent, 2MNC-Robust proposes an approximation of the posterior on good features and uses a ranking strategy similar to 2MNC-DGIB to select features [8].

While 2MNC-DGIB has outstanding performance when its assumptions are satisfied [7], it performs poorly when bad features are dependent [9]. On the other hand, CMNC-OBF and 2MNC-Robust have been shown to have robust performance across Bayesian models with block-diagonal covariances [9]. CMNC-OBF is extremely fast and enjoys particularly excellent performance when markers are individually strong with low correlations, but, like all filter methods, may miss weak features that are of interest due to high correlations with strong features [6, 9]. 2MNC-Robust is computationally very manageable and generally improves upon CMNC-OBF in the presence of correlations.

Although CMNC-OBF and 2MNC-Robust are robust to different block-diagonal covariance structures, they do not attempt to detect these underlying structures, and their assumptions and approximations constrain performance. Thus, in this work we propose three new feature selection algorithms that: (1) use an iterative strategy to update the approximate posterior used in 2MNC-Robust, (2) use a novel scoring function inspired by Bayes factors to improve overall rankings, and (3) attempt to actually detect the underlying block structure of the data. We show that these algorithms have comparable computation time to 2MNC-Robust, while outperforming 2MNC-Robust and many other popular feature selection algorithms on a synthetic Bayesian model assuming block-diagonal covariance matrices, and a synthetic microarray data model. Finally, we apply the proposed algorithms and CMNC-OBF to breast cancer, colon cancer, and AML datasets, and perform enrichment analysis on each to address validation.

## Feature selection model

We review a hierarchical Bayesian model that serves as a reference for the approximate posterior developed in 2MNC-Robust [8, 9] and will be used in the algorithms we present in the next section.

Consider a binary feature selection problem with class labels *y*=0,1. Let *F* be the set of feature indices. Assume features are partitioned into blocks, where features in each block are dependent, but features in different blocks are independent. Assume each block is either good or bad. A good block has different class-conditioned distributions between the two classes, while a bad block has the same distribution in both classes. We denote a partitioning of *F* to good and bad blocks by *P*=(*P*_*G*_,*P*_*B*_), and hereafter call it a feature partition, where *P*_*G*_={*G*_1_,⋯,*G*_*u*_} is the set of *u* good blocks and *P*_*B*_={*B*_1_,⋯,*B*_*v*_} is the set of *v* bad blocks. Furthermore, denote the set of all features in good blocks as good features, $G=\cup _{i=1}^{u} G_{i}$, and denote all features in bad blocks as bad features, $B=\cup _{j=1}^{v} B_{j}$. Denote the random feature partition by $\bar {P}=(\bar {P}_{G},\bar {P}_{B})$, the random set of good features by $\bar {G}$, and the random set of bad features by $\bar {B}$.

We define $\pi (P)=\mathrm {P}(\bar {P}=P)$ to be the prior distribution on $\bar {P}$. Let *P* be fixed. Let *θ*^*P*^ be the parameter describing the joint feature distribution of *P*. Since blocks are independent of each other we can write $\theta ^{P}\,=\,\left [\!\theta ^{G_{1}}_{0},\cdots,\theta ^{G_{u}}_{0},\theta ^{G_{1}}_{1},\cdots,\theta ^{G_{u}}_{1},\theta ^{B_{1}},\cdots,\theta ^{B_{v}}\right ]$, where $\theta ^{G_{i}}_{y}$ parametrizes class-*y* features in *G*_*i*_, and $\phantom {\dot {i}\!}\theta ^{B_{j}}$ parametrizes features in *B*_*j*_. Assume $\theta ^{G_{i}}_{y}$ and $\phantom {\dot {i}\!}\theta ^{B_{j}}$’s are independent given *P*, i.e., $\pi \left (\theta ^{P}\right)=\prod _{i=1}^{u} \pi \left (\theta ^{G_{i}}_{0}\right) \pi \left (\theta ^{G_{i}}_{1}\right) \prod _{j=1}^{v} \pi \left (\theta ^{B_{j}}\right)$.

Given a training set, , of *n* independent and identically distributed (i.i.d.) points, with *n*_*y*_ points in each class, we have  and , where *π*^∗^(.) denotes posterior,  and  are class-*y* points in *G*_*i*_ and points in *B*_*j*_, respectively, and  and  are the likelihoods. Following steps in [7, 10], we have 
1

In addition, the marginal posterior of a feature set *G* is , and marginal posterior of a feature *f* is . Note  is different than .

### Gaussian model

Here we solve Eq. (1) for jointly Gaussian features. We assume for a block *A*, $\theta ^{A}_{y}=\left [\mu ^{A}_{y}, \Sigma ^{A}_{y}\right ]$ and *θ*^*A*^=[*μ*^*A*^,*Σ*^*A*^], where $\mu ^{A}_{y}$ and *μ*^*A*^ are the mean vectors, and $\Sigma ^{A}_{y}$ and *Σ*^*A*^ are the covariance matrices.

Let *P* be a feature partition. Suppose *A* is a good block of *P*. Assume $\pi (\theta ^{A}_{y})$ is Normal-Inverse-Wishart (NIW). Hence, $\pi \left (\theta ^{A}_{y}\right)=\pi \left (\Sigma ^{A}_{y}\right) \pi \left (\mu ^{A}_{y}|\Sigma ^{A}_{y}\right)$, where

$$ \begin{aligned} \pi\left(\Sigma^{A}_{y}\right) &= K^{A}_{y} |\Sigma^{A}_{y}|^{-\frac{\kappa^{A}_{y}+|A|+1}{2}} \text{exp}\left(-0.5 \text{Tr}\left(S^{A}_{y} \left(\Sigma^{A}_{y}\right)^{-1}\right) \right),  \\ \pi\left(\mu^{A}_{y}|\Sigma^{A}_{y}\right) &= L^{A}_{y} |\Sigma^{A}_{y}|^{-0.5}  \\ &\quad\times \text{exp}\left(\!\!-0.5 \nu^{A}_{y} \left(\mu^{A}_{y}\,-\,m^{A}_{y}\right)^{T} \!\left(\!\Sigma^{A}_{y}\right)^{-1}\! \left(\mu^{A}_{y}-m^{A}_{y}\right)\!\! \right)\!,  \end{aligned}  $$

where for a matrix |.| denotes determinant. $S^{A}_{y}, \kappa ^{A}_{y}, m^{A}_{y}$, and $\nu ^{A}_{y}$ are hyperparameters, which are assumed given and fixed. $S^{A}_{y}$ is an |*A*|×|*A*| matrix, where for a set |.| denotes cardinality. For a proper prior $S^{A}_{y}$ is symmetric and positive-definite, and $\kappa ^{A}_{y}>|A|-1$. If $\kappa ^{A}_{y}>|A|+1$, then $E\left (\Sigma ^{A}_{y}\right)=S^{A}_{y}/\left (\kappa ^{A}_{y}-|{A}|-1\right)$. Furthermore, $m^{A}_{y}$ is an |*A*|×1 vector describing the average mean of features and for a proper prior we need $\nu ^{A}_{y}>0$. $K^{A}_{y}$ and $L^{A}_{y}$ represent the relative weights of each distribution. For a proper distribution we have $K^{A}_{y}=|S^{A}_{y}|^{0.5 \kappa ^{A}_{y}}2^{-0.5 \kappa ^{A}_{y} |{A}|} / \Gamma _{|{A}|}\left (0.5 \kappa ^{A}_{y}\right)$ and $L^{A}_{y}=\left (2\pi /\nu ^{A}_{y}\right)^{-0.5|{A}|}$, where *Γ*_*d*_ denotes the multivariate gamma function.

Since NIW is a conjugate prior of Gaussian distribution, given sample, $\pi ^{*}\left (\theta ^{A}_{y}\right)$ is again NIW with updated hyperparameters: $\kappa ^{A^{*}}_{y}=\kappa ^{A}_{y}+n_{y}$, $\nu ^{A^{*}}_{y}=\nu ^{A}_{y}+n_{y}$, $m^{A^{*}}_{y}=\frac {\nu ^{A}_{y} m^{A}_{y}+ n_{y} \hat {\mu }^{A}_{y}}{\nu ^{A^{*}}_{y}}$, and

$$ {}S^{A^{*}}_{y}\!=S^{A}_{y}+(n_{y}-1) \hat{\Sigma}^{A}_{y}+\frac{\nu^{A}_{y} n_{y}}{\nu^{A}_{y} + n_{y}} \left(\hat{\mu}^{A}_{y}-m^{A}_{y}\right)\left(\hat{\mu}^{A}_{y}-m^{A}_{y}\right)^{T},   $$

where $\hat {\mu }^{A}_{y}$ and $\hat {\Sigma }^{A}_{y}$ are class-conditioned sample mean and covariance of , respectively [11]. Now suppose *A* is a bad block. We assume the prior on *θ*^*A*^ is NIW with hyperparameters *S*^*A*^,*κ*^*A*^,*m*^*A*^, and *ν*^*A*^, and relative weights *K*^*A*^ and *L*^*A*^. Given sample, *π*^∗^(*θ*^*A*^) is NIW with $\kappa ^{A^{*}}=\kappa ^{A}+n$, $\nu ^{A^{*}}=\nu ^{A}+n$, $m^{A^{*}}=\frac {\nu ^{A} m^{A}+ n \hat {\mu }^{A}}{\nu ^{A^{*}}}$, and

$${}  S^{A^{*}}=S^{A}+(n-1) \hat{\Sigma}^{A}+\frac{\nu^{A} n}{\nu^{A}+n} \left(\hat{\mu}^{A}-m^{A}\right)\left(\hat{\mu}^{A}-m^{A}\right)^{T},   $$

where $\hat {\mu }^{A}$ and $\hat {\Sigma }^{A}$ are sample mean and covariance of , respectively [11]. As long as *π*^∗^(*P*) is proper, using the normalization constant of NIW distribution to compute the integrals in Eq. (1) we have

$$\begin{array}{*{20}l} \pi^{*}(P) &\propto \pi(P) \prod\limits_{i=1}^{u} Q^{G_{i}}_{0} Q^{G_{i}}_{1} \left|S^{G_{i}^{*}}_{0}\right|^{-0.5 \kappa^{G_{i}^{*}}_{0}} \left|S^{G_{i}^{*}}_{1}\right|^{-0.5 \kappa^{G_{i}^{*}}_{1}}  \\ &\times \prod\limits_{j=1}^{v} Q^{B_{j}} |S^{B_{j}^{*}}|^{-0.5 \kappa^{B_{j}^{*}}},  \end{array} $$

where

$$\begin{array}{*{20}l} Q^{A}_{y} &=K^{A}_{y} L^{A}_{y} 2^{0.5 \kappa^{A*}_{y} |A|} \Gamma_{|A|}\left(0.5\kappa^{A^{*}}_{y}\right) \left({2 \pi}/{\nu^{A^{*}}_{y}} \right)^{0.5|A|},  \\ Q^{A} &=K^{A} L^{A} 2^{0.5 \kappa^{A*} |A|} \Gamma_{|A|}\left(0.5 \kappa^{A^{*}}\right) \left({2 \pi}/{\nu^{A^{*}}} \right)^{0.5|A|}.  \end{array} $$

Assuming: (1) *π*(*P*) is such that the block structure, i.e., the number and size of good and bad blocks, is fixed, (2) for each good block *A*, $K^{A}_{y}$, $L^{A}_{y}$, $\kappa ^{A}_{y}$, and $\nu ^{A}_{y}$ do not depend on the features indices in A, and (3) for each bad block *A*, *K*^*A*^, *L*^*A*^, *κ*^*A*^, and *ν*^*A*^ do not depend on the features indices in *A*,

$$\begin{array}{*{20}l}{} \pi^{*}(P) \propto \pi(P) \left(\prod\limits_{i=1}^{u} \left|S^{G_{i}^{*}}_{0}\right|^{\kappa^{G_{i}^{*}}_{0}} \left|S^{G_{i}^{*}}_{1}\right|^{\kappa^{G_{i}^{*}}_{1}} \prod\limits_{j=1}^{v} \left|S^{B_{j}^{*}}\right|^{\kappa^{B_{j}^{*}}} \right)^{-0.5}.  \end{array} $$

## Methods

Here we describe the set selection methods used. Note we aim to find the set of true good features, rather than the true underlying feature partition. The Maximum Number Correct (MNC) criterion [7] outputs the set maximizing the expected number of correctly labeled features and the Constrained MNC (CMNC) criterion outputs the set with maximum expected number of correctly labeled features constrained to having exactly *D* selected features, where *D* is a parameter of the optimization problem [9]. The solution of MNC is {*f*∈*F*:*π*^∗^(*f*)>0.5} [7] and the solution of CMNC is picking the top *D* features with largest *π*^∗^(*f*) [9]. Therefore, both MNC and CMNC require computing *π*^∗^(*f*) for all *f*∈*F*, which is not computationally feasible for an arbitrary block structure unless |*F*| is very small. We review two previously proposed algorithms, OBF and 2MNC-Robust, and then present three new algorithms.

### Optimal Bayesian filter

Optimal Bayesian Filter (OBF) assumes all blocks have size one, i.e., all features are independent, and assumes the events $\{f \in \bar {G}\}$ are independent a priori. In this case *π*^∗^(*f*) can be found in closed form with little computation cost [6, 9]. OBF is optimal under its modeling assumptions. As argued in [9], in the presence of correlation OBF is a robust suboptimal algorithm that can detect individually strong good features, i.e., those whose mean and/or variance is very different between the two classes, but cannot take advantage of correlations to correctly label individually weak good features, those whose mean and variance are similar in both classes.

### 2MNC-Robust

The 2MNC algorithm [7] suggests approximating *π*^∗^(*f*) using *π*^∗^(*G*) for all sets *G* such that |*G*|=2, and picking the top *D* features. Since finding *π*^∗^(*G*) for all feature partitions where |∪*P*_*G*_|=2 is typically infeasible, an approximate posterior, $\tilde {\pi }^{*}(G)$, is proposed [8], where for all *G*⊆*F* of size 2,



The normalization constant is found such that $\sum _{G \subseteq F: |G|=2} \tilde {\pi }^{*}(G) = 1$. $\tilde {\pi }(G)$ mimics the role of $\pi (G)=P(\bar {G}=G)=\sum _{P:\cup P_{G}=G} \pi (P)$. Using some suboptimal method might affect one’s decision of the value used as the prior of a feature set, replacing *π*^∗^(*G*) with $\tilde {\pi }^{*}(G)$. For example, knowing $|\bar {G}|>2$ implies *π*(*G*)=0 for all sets of size 2; however, 2MNC-Robust only evaluates such sets. In this case, $\tilde {\pi }(G)=P(G \subseteq \bar {G})$ might be a suitable choice to replace *π*(*G*). $\tilde {\pi }^{*}(f)=\sum _{G: f \in G} \tilde {\pi }^{*}(G)$ is the approximate marginal posterior of *f*∈*F*. For the Gaussian model, if the number of good features is fixed and hyperparameters do not depend on the feature indices,

2 $$\begin{array}{*{20}l}  \tilde{\pi}^{*}(G) &\propto \tilde{\pi}(G) \left({|S^{G^{*}}_{0}|^{\kappa^{G^{*}}_{0}} |S^{G^{*}}_{1}|^{\kappa^{G^{*}}_{1}}}\big/{ |S^{G^{*}}|^{\kappa^{G^{*}}}} \right)^{-0.5}. \end{array} $$

2MNC-Robust is implementing 2MNC with $\tilde {\pi }^{*}(f)$. As mentioned before, 2MNC-Robust does not tune itself to the underlying block structure of data.

### Recursive marginal posterior inflation

It is easy to show that $\sum _{f\in F} \tilde {\pi }^{*}(f)=2$ when only sets of size 2 are used to find $\tilde {\pi }^{*}(f)$. Hence, under MNC criterion one would at most pick 4 good features, implying we underestimate *π*^∗^(*f*) by only using sets of size 2 when $|\bar {G}|>>2$. REcursive MArginal posterior INflation (REMAIN) aims to sequentially detect good features by rescaling $\tilde {\pi }^{*}(f)=\sum _{G:f\in G,|G|=2} \tilde {\pi }^{*}(G)$. We initialize REMAIN with the set of all features, *F*_*r*_=*F*. Then, REMAIN uses the MArginal posterior INflation (MAIN) algorithm to identify several features as good, removes them from *F*_*r*_, and feeds MAIN with the truncated *F*_*r*_ to select additional features. This process iterates until MAIN does not output any features. REMAIN is nothing but repetitive calls to MAIN with shrinking feature sets, making MAIN the heart of this algorithm.



Pseudo-code of MAIN is provided in Algorithm 1, where *H*(*G*) is the right hand side of Eq. (2). Inputted with a feature set *F*_*t*_, MAIN finds $\tilde {\pi }^{*}(f)$ using sets of size 2, and finds the set $G_{s}=\{f \in F_{t} : \tilde {\pi }^{*}(f)>T_{1} \}$. MAIN adds *G*_*s*_ to $\tilde {G}$, the set of features in *F*_*t*_ already labeled as good. It then updates *F*_*t*_ to *F*_*t*_∖*G*_*s*_, and rescales $\tilde {\pi }^{*}(f)$ of features *f*∈*F*_*t*_ so that $\sum _{f \in F_{t}} \tilde {\pi }^{*}(f)=2$. Note features in $\tilde {G}$ are used to compute $\tilde {\pi }^{*}(f)$ for features *f*∈*F*_*t*_, but $\tilde {\pi }^{*}(f)$ of features $f \in \tilde {G}$ are not used in the scaling of $\sum _{f \in F_{t}} \tilde {\pi }^{*}(f)=2$. MAIN iterates until $\tilde {G}=\phi $, or *H*(*G*)≤*T*_2_ for all *G*⊆*F*_*t*_ with |*G*|=2.

Not finding new features in MAIN might be due to the remaining good features being weaker and independent of $\tilde {G}$. Hence, REMAIN removes $\tilde {G}$ from *F*_*r*_, and feeds MAIN with the updated *F*_*r*_. This way, features in $\tilde {G}$ are not used to compute $\tilde {\pi }^{*}(f)$ anymore for any feature *f*∈*F*_*r*_, thus making it easier to detect weaker good features that are independent of features already selected by REMAIN. Pseudo-code of REMIAN is provided in Algorithm 2.

*T*_1_ mimics the role of the threshold used in the MNC criterion. Hence, *T*_1_∈[ 0, 1]. Recall that by evaluating sets of size 2 we underestimate $\tilde {\pi }^{*}(f)$ when $|\bar {G}|>>2$. Therefore, when confident $|\bar {G}|>>2$, one might opt for smaller values for *T*_1_ rather than values close to 1. As *T*_2_ is a threshold over un-normalized posteriors, *H*(*G*), extra care must be taken when setting *T*_2_. We suggest *T*_2_=*n* for high-dimensional feature selection applications, which is a good rule of thumb based on our simulation results and asymptotic analysis of *H*(.).



Note the number of features reported by REMAIN is variable; however, one can easily obtain close to a desired number of selected features by tuning *T*_1_. To illustrate, we provide an example based on the data generation model used in the “Synthetic microarray simulations” section, where we assume there are 100 markers, i.e., good features, and 4900 non-markers, i.e., bad features. We use the synergetic model with block size *k*=5 and correlation coefficients *ρ*_0_=*ρ*_1_=0.9. Fixing *T*_2_=*n*, Table 1 lists the average number of markers and non-markers selected over 1000 iterations for *n*=20 and 100 across different values of *T*_1_. REMAIN outputs very few features when *T*_1_ is large, and too many features when *T*_1_ is extremely low. The best choice for *T*_1_ can vary greatly from case to case, but one strategy is to choose *T*_1_ so that REMAIN selects close to a given number of features. For example, *T*_1_=0.05 is a good choice in this simulation if one desires approximately 100 selected features. Another strategy is based on the number of features selected by REMAIN across various values of *T*_1_. When *T*_1_ is large, reducing *T*_1_ only slightly increases the output feature size, for instance when *T*_1_>0.05 in this simulation. However, one might observe a rapid increase in the output size by slightly reducing *T*_1_, for instance *T*_1_ changing from 0.05 to 0.01 in this simulation. For such observed patterns, the value for which this phenomenon occurs might be a desirable choice.

**Table 1:** Performance of REMAIN for various values of *T*_1_

Objective	n	*T*_1_=0.3	*T*_1_=0.2	*T*_1_=0.1	*T*_1_=0.05	*T*_1_=0.01	*T*_1_=0.005
Marker found	20	0.9	1.4	2.5	4.6	15.3	26.2
Non-marker found	20	2.0	3.7	9.4	23.3	164.5	403.0
Marker found	100	52.5	56.2	58.2	60.0	68.9	76.1
Non-marker found	100	1.6	3.3	8.8	20.2	124.7	288.5

### Posterior factor

Feature selection can be construed as a model selection problem where each model is a set of good features. Let *f* be a feature. If *f* is a good feature, we expect that if we add *f* to any model *G*, i.e., a set of good features, then $\tilde {\pi }^{*}(G \cup \{\,f\}) / \tilde {\pi }^{*}(G)>>1$. If *f* is a bad feature, we expect $\tilde {\pi }^{*}(G \cup \{\,f\}) / \tilde {\pi }^{*}(G)$ to be much smaller. Hence, if we average this ratio over a family of models that do not contain *f*, and denote it by *β*(*f*), we expect *β*(*f*)>>1 if *f* is a good feature, and comparable to or smaller than 1 if *f* is a bad feature. $\tilde {\pi }^{*}(G \cup \{\,f\}) / \tilde {\pi }^{*}(G)$ is similar, but not identical, to the *Bayes factor* encountered in model selection [12], where here we compare a model with good feature set *G*∪{ *f*} versus a model with good feature set *G* excluding *f*. The posterior factor, *β*(*f*), averages the approximate posterior ratio across all feature sets *G*∈*F*∖{ *f*}, i.e.,

3 $$\begin{array}{*{20}l} \beta(f)=\frac{1}{|\Omega^{f}|} \sum_{G \in \Omega^{f}} \frac{\tilde{\pi}^{*}(G \cup \{\,f\})}{\tilde{\pi}^{*}(G)}, \end{array} $$

where *Ω*^*f*^={*G*⊆*F*:*f*∉*G*}. As the summation over *Ω*^*f*^ is computationally infeasible, we propose *approximate posterior factor*, hereafter denoted by $\tilde {\beta }$.

4 $$\begin{array}{*{20}l} \tilde{\beta}(f)=\frac{1}{|F|-1} \sum_{f' \in F \backslash \{f\}} \frac{\tilde{\pi}^{*}(\{\,f,f' \})}{\tilde{\pi}^{*}(\{\,f'\})}. \end{array} $$

We propose *approximate POsterior FActor-Constrained* (POFAC) algorithm as follows: Use $\tilde {\beta }(\,f)$ to rank features, and pick the top *D* features. Note that *D* is a parameter of the algorithm.

### Sequential partition mustering

Sequential Partition Mustering (SPM) aims to improve feature selection performance by sequentially detecting good blocks, and adding them to the set of previously selected features. To find a good block, we start with the most significant feature, i.e., the feature with largest $\tilde {\beta }$, and find the block containing it. Note we do not aim to find the structure of bad blocks, and as soon as we declare no more good features remain the algorithm terminates.

Suppose *u*_0_ is the current most significant feature. In order to find the block containing *u*_0_ we propose the Good Seed Grower (GSG) algorithm, which can be construed as a seed growing algorithm with *u*_0_ as the seed. Pseudo-code of GSG is presented in Algorithm 3, where for any two non-empty disjoint sets *G*_1_,*G*_2_⊂*F*,

$${{\begin{aligned} C_{1}(G_{1},G_{2}) &= \frac{\tilde{\pi}(G_{1},G_{2})}{1-\tilde{\pi}(G_{1},G_{2})} \frac{Q^{G_{12}}_{0} Q^{G_{12}}_{1}} {Q^{G_{1}}_{0} Q^{G_{1}}_{1} Q^{G_{2}}_{0} Q^{G_{2}}_{1}} \\ &\quad\times \left(\frac{\left| S^{G^{*}_{12}}_{0}\right|^{\kappa^{G^{*}_{12}}_{0}} \left| S^{G^{*}_{12}}_{1}\right|^{\kappa^{G^{*}_{12}}_{1}} } {\left| S^{G^{*}_{1}}_{0} \right|^{\kappa^{G^{*}_{1}}_{0}} \left| S^{G^{*}_{1}}_{1} \right|^{\kappa^{G^{*}_{1}}_{1}} \left| S^{G^{*}_{2}}_{0} \right|^{\kappa^{G^{*}_{2}}_{0}} \left| S^{G^{*}_{2}}_{1} \right|^{\kappa^{G^{*}_{2}}_{1}} } \right)^{-0.5}, \end{aligned}}} $$

*G*_12_=*G*_1_∪*G*_2_, and $\tilde {\pi }(G_{1},G_{2})$ approximates *π*(*G*_1_,*G*_2_), the prior probability that at least one of the features in *G*_2_ is not independent of *G*_1_. Note , where  is the family of feature partitions that contain a block *U* such that *U*∩*G*_1_≠*ϕ* and *U*∩*G*_2_≠*ϕ*. At each iteration, GSG finds the feature *u*^∗^ that maximizes *C*_1_(*U*,{*u*^∗^}), where *U* is the currently detected sub-block of the block containing *u*_0_. GSG declares *u*^∗^ and *U* belong to the same block if *C*_1_(*U*,{*u*^∗^})>*T*_3_, and adjoins *u*^∗^ to *U*; otherwise, it terminates and declares *U* as the block containing *u*_0_. Here we assume $\phantom {\dot {i}\!}T_{3}=t_{1} n^{t_{2} |U|}$, where *t*_1_,*t*_2_>0 are parameters of GSG. While we have only considered one possible family of thresholds, we expect this family to be large enough for most practical purposes.



Pseudo-code of SPM is explained in Algorithm 4. Let *F*_*t*_ be the feature set used by SPM initialized to *F*_*t*_=*F*. We start with the most significant feature *u*_0_ and find the block containing it, *U*. We then update *F*_*t*_ to *F*_*t*_∖*U*. If $\tilde {\beta }(f)<T_{4}$ for all *f*∈*F*_*t*_, then SPM declares *F*_*t*_ does not contain any good features and terminates; otherwise, it picks the most significant feature of *F*_*t*_ and iterates. Similar to REMAIN, SPM cannot be forced to output a fixed number of features, but *T*_4_ can be used to tune SPM to output close to a desired number of features. In addition, *t*_1_ and *t*_2_ can be used to avoid picking large blocks, which also affects the output feature set size.



**Fig. 1:**
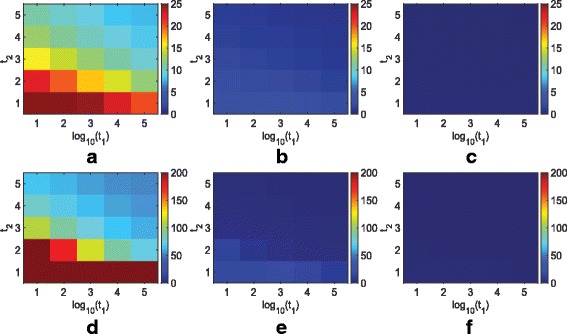
Performance of SPM for various values of *t*_1_, *t*_2_, *T*_4_, and *n*=20. Average number of markers labeled as good for **a** *T*_4_=10^2^, **b** *T*_4_=10^4^, and **c** *T*_4_=10^6^. Average number of non-markers labeled as good for **d** *T*_4_=10^2^, **e** *T*_4_=10^4^, and **f** *T*_4_=10^6^

**Fig. 2:**
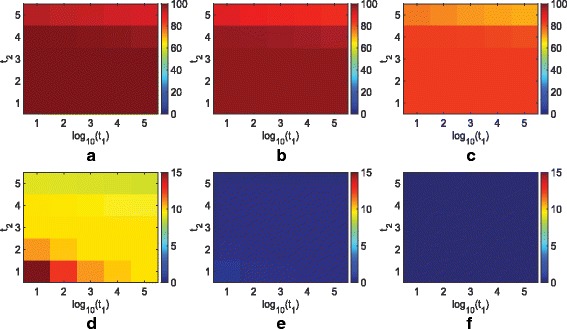
Performance of SPM for various values of *t*_1_, *t*_2_, *T*_4_, and *n*=100. Average number of markers labeled as good for **a** *T*_4_=10^2^, **b** *T*_4_=10^4^, and **c** *T*_4_=10^6^. Average number of non-markers labeled as good for **d** *T*_4_=10^2^, **e** *T*_4_=10^4^, and **f** *T*_4_=10^6^

We again provide an example based on the simulation we did for REMAIN. We let *t*_1_=10^1^,10^2^,⋯,10^5^,*t*_2_=1,2,3,4,5, and *T*_4_=10^2^,10^4^,10^6^, and consider all combinations to construct a very wide range of parameters. Figures 1 and 2 illustrate how parameters of SPM affect its performance for *n*=20 and 100, respectively. While low thresholds mislabel more non-markers as good features, they correctly label more markers compared with large thresholds. When *n*=20, in order to correctly label at least 10 markers on average, at least 50 non-markers are mislabeled, and to mislabel at most 5 non-markers on average, one cannot correctly detect more than 5 markers. On the other hand, when *n*=100, one can simultaneously correctly label at least 80 markers and mislabel at most 10 non-markers for almost all parameters. Moving from lowest parameter values to highest, we observe the average outputted feature size varies from approximately 400 to less than 5 when *n*=20, while it only varies from 120 to 70 when *n*=100. Thereby, *n*=100 is less sensitive to the choice of parameters than *n*=20. Suppose *n*=100 and one aims to find most markers and few non-markers. Many parameters can achieve this goal. In addition, within the range of such parameters, the number of features labeled as good does not change very much by slightly varying the parameters. Hence, for a fixed sample, one can implement SPM over a wide range of parameters, find the range where the output feature size does not vary much by slightly changing the parameters, and pick a value in that region that outputs a feature set with close to a reasonable number of features.

## Simulations

We compare the performance of proposed algorithms with many popular feature selection algorithms over a Bayesian setting, and a synthetic microarray model introduced in [13] and extended in [8, 9].

### Bayesian simulation

In this simulation we assume |*F*|=4100 and $|\bar {G}|=100$. We assume there is 1 good block for each of the following sizes: 10, 20, 30, and 40. We also assume there are 20 bad blocks for each of the following sizes: 5, 10, 15, 20, 50, and 100. We first randomly assign each feature to a block such that the assumed block structure is satisfied, effectively constructing $\bar {P}$. Afterwards, distribution parameters are randomly drawn from the following NIW prior. For each good block, *A*, we have $S^{A}_{0}=S^{A}_{1}=0.5 \times I_{|A| \times |A|}, \kappa ^{A}_{0}=\kappa ^{A}_{1}=|A|+2, m^{A}_{0}=m^{A}_{1}=0$, and $\nu ^{A}_{0}=\nu ^{A}_{1}=4$, where *I* is the identity matrix. Also, for a bad block, *A*, we have *S*^*A*^=0.5×*I*_|*A*|×|*A*|_,*κ*^*A*^=|*A*|+2,*m*^*A*^=0, and *ν*^*A*^=4. Given distribution parameters, a stratified sample of size *n* with equal points in each class is drawn. The following feature selection methods declare the set of good features: t-test, Bhatacharrya Distance (BD), Mutual Information (MI) using the non-parameter method of [14] with spacing parameter *m*=1, Sequential Forward Search using the bolstered error estimate [15] of Regularized Linear Discriminant Analysis applied to the top 300 features of BD (SFS-RLDA), FOrward selection using Hilbert-Schmidt Independence Criterion (FOHSIC) [16] applied to the top 300 features of BD, CMNC-OBF, 2MNC-Robust, REMAIN, POFAC, and SPM. Note t-test, MI, BD, and CMNC-OBF are filter methods. All methods except REMAIN and SPM output $|\bar {G}|$ features. CMNC-OBF assumes the events $\{f \in \bar {G} \}$ are independent and $P(f \in \bar {G})$ is constant for all *f*∈*F*. 2MNC-Robust and REMAIN assume $\tilde {\pi }(G)$ is uniform over all sets of size 2, and zero otherwise. POFAC assumes $\tilde {\pi }(G)$ is uniform over all sets of size 1 and 2. Finally, SPM assumes $\tilde {\pi }(G_{1},G_{2})=0.5$ for all sets *G*_1_,*G*_2_⊆*F*, and uses the same $\tilde {\pi }(G)$ of POFAC to compute $\tilde {\beta }(f)$. Bayesian algorithms use proper priors with hyperparameters of the same form given previously (PP), and Jeffreys non-informative prior (JP), where for each set, *A*, $S^{A}_{y}$ and *S*^*A*^ are zero matrices, $K^{A}_{y}=K^{A}=L^{A}_{y}=L^{A}=1$, and $\kappa ^{A}_{y}=\kappa ^{A}=\nu ^{A}_{y}=\nu ^{A}=0$. With $\nu ^{A}_{y}=\nu ^{A}=0$ we do not need to specify $m^{A}_{y}$ and *m*^*A*^. We use *T*_1_=0.3 and *T*_2_=*n* for REMAIN using both PP and JP. For SPM-PP we set *t*_1_=100, *t*_2_=0.5, and *T*_4_=100*n*^2^, which resulted in adequate performance among all sample sizes. When using SPM-JP we use the same *t*_1_ and *T*_4_, but set *t*_2_=1 to avoid picking large blocks. This process iterates 1000 times.

**Fig. 3:**
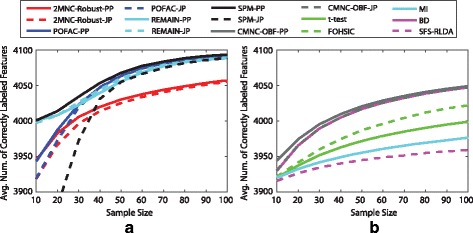
Performance of various feature selection algorithms under Gaussian data. Average number of correctly labeled features versus sample size for randomly generated parameters for (**a**) 2MNC-Robust-PP, 2MNC-Robust-JP, POFAC-PP, POFAC-JP, REMAIN-PP, REMAIN-JP, SPM-PP, SPM-JP, (**b**) CMNC-OBF-PP, CMNC-OBF-JP, t-test, FOHSIC, MI, BD, and SFS-RLDA

Figure 3 plots the average number of correctly labeled features as sample size increases from 10 to 100 in steps of 10. SPM-PP has the best performance; however, SPM-JP experiences a sharp drop under small sample sizes. For larger sample sizes, POFAC-PP performs second only to SPM-PP. However, POFAC-JP outperforms SPM-JP. REMAIN adequately balances performance across all sample sizes. All proposed algorithms, except SPM-JP, outperform 2MNC-Robust, CMNC-OBF, and other feature selection algorithms. SPM-JP outperforms previous algorithms if sample size is not very small.

In this simulation filter methods were the fastest with comparable computation time, and FOHSIC was the most computationally intensive method. A comparison of run-times for this specific simulation is provided in Table 2 assuming the run-time of 2MNC-Robust is the unit of time. Parallel processing can be used to speed up these algorithms, for instance, in the 4th step of GSG, and to compute $\tilde {\pi }^{*}(G)$ in 2MNC-Robust and POFAC. Although SPM is a sequential algorithm, its bottle-neck is step 4 of GSG, making parallel processing a good strategy to extensively speed up SPM.

**Table 2:** Run-time comparison of Bayesian simulation

Alg.	Filter	2MNC-Robust	REMAIN	POFAC	SPM	SFS-RLDA	FOHSIC
Time	< 10^−3^	1	2	1.05	1.5	10	15

### Synthetic microarray simulations

Here an extended version of a synthetic model developed to mimic microarrays is used to generate data. The original model is introduced in [13], and has been extended in [8, 9]. In these models features are markers or non-markers. Markers are either global or heterogeneous. Global markers (GM) are homogeneous within each class. Heterogeneous Markers (HM) compromise *c* subclasses, where for each specific set of heterogeneous markers, a specific subset of the training sample has a different distribution than markers in class 0, and the remaining sample points have the same distribution as class 0. Markers comprise blocks of size *k*, where each block in class *y* is Gaussian with mean *μ*_*y*_ and covariance *σ*_*y*_*Σ*_*y*_. Diagonal elements of *Σ*_*y*_ are 1 and non-diagonal elements are *ρ*_*y*_. The original model of [13] forced *ρ*_0_=*ρ*_1_. We also have *μ*_0_ = [ 0,⋯,0]. There are three types of markers according to their mean in class 1: redundant, synergetic, and marginal, with *μ*_1_ being [ 1,⋯,1],[ 1,1/2,⋯,1/*k*], and [ 1,0,⋯,0], respectively. Non-markers are either Low Variance (LV) or High Variance (HV). In the original model LV non-markers are independent, each with a Gaussian distribution, *N*(0,*σ*_0_). However, in the extended model of [8, 9], similar to markers in class 0, LV non-markers comprise blocks of size *k*, where in each block features are jointly Gaussian with mean *μ*_0_ and covariance *σ*_0_*Σ*_0_. HV non-markers are independent with marginal distribution *pN*(0,*σ*_0_)+(1−*p*)*N*(1,*σ*_1_), where *p* is drawn from the uniform distribution over [ 0,1].

**Fig. 4:**
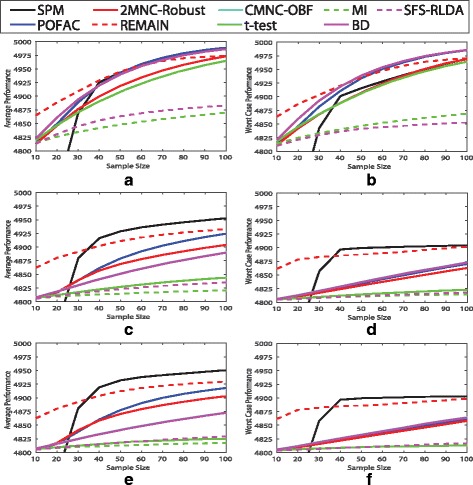
Average and worst case performance of feature selection algorithms. Average and worst case performance are obtained using 27 combinations of the synthetic microarray model parameters *k*, *ρ*_0_ and *ρ*_1_ with fixed mean type, where performance is defined to be the average number of markers identified as good plus the average number of non-markers identified as bad over 500 iterations: **a** average performance for redundant means, **b** worst case performance for redundant means, **c** average performance for synergetic means, **d** worst case performance for synergetic means, **e** average performance for marginal means, and **f** worst case performance for marginal means

We assume |*F*|=5000,|*GM*|=20,|*HM*|=80,|*HV*|=2000, and *c*=2. We consider all possible combinations of the following parameters: all 3 mean types, *k*=5,10,20, and *ρ*_0_,*ρ*_1_=0.1,0.5,0.9. We also consider the “large and unequal variance” setting of Table 1 in [13], which sets *σ*_0_=0.25 and *σ*_1_=0.64. Given each set of distribution parameters, we randomly assign features to blocks of global markers, heterogeneous markers, and LV non-markers. The remaining features comprise the independent HV non-markers. We generate a stratified sample of size *n* with equal points in each class. The following algorithms are used to declare the set of good features: t-test, BD, MI, SFS-RLDA, CMNC-OBF, 2MNC-Robust, REMAIN, POFAC, and SPM. We removed FOHSIC due to its computation cost. All Bayesian algorithms use JP. We use thresholds of the Bayesian simulation, except we set *T*_1_=0.05. One can tune *T*_3_ and *T*_4_ for one of the 81 possible settings, or a specific sample size, but it can affect the performance of other settings. We picked the thresholds of the Bayesian simulation as they provided satisfactory performance among large sample sizes. This process iterates 500 times. For each set of distribution parameters we define performance as the average number of markers identified as good plus the average number of non-markers identified as bad. Figure 4 plots the average and worst case performance for each fixed mean type across other distribution parameters as sample size increases from 10 to 100 in steps of 10. Bayesian methods tend to outperform non-Bayesian methods. While simpler methods such as CMNC-OBF outperform more complicated methods when sample size is small, complicated methods such as SPM have superior performance when sample size is large.

For small sample sizes, or cases where correctly labeling good features is more difficult, REMAIN tends to output very few features resulting in very good performance, in contrast to methods that are forced to output $|\bar {G}|=100$ features. OBF can be implemented with the MNC objective instead of CMNC to enjoy this characteristic of REMAIN. SPM seems to have the most diverse behavior. While it performs inferior to all feature selection algorithms when sample size is very small, it tends to outperform all other methods for larger sample sizes. In order for the quantities used in SPM to be well-defined under JP, sample size must be larger than the block size. Hence, under small samples SPM with JP tends to break good blocks into smaller blocks, thereby losing some of its ability to identify weak good features with strong dependencies, and making it more prone to detecting blocks incorrectly. Also note that we have used the same parameters for SPM across all data models and sample sizes, and performance is expected to improve if *t*_1_,*t*_2_ and *T*_4_ are calibrated each time it is run.

POFAC is an interesting option, enjoying competitive performance across all sample sizes. It outperforms 2MNC-Robust while its computation cost is only slightly larger. CMNC-OBF tends to select individually strong markers, i.e., markers with class 1 mean far from 0. CMNC-OBF performs very similar to BD in this simulation, with their performance graphs almost overlapping.

**Fig. 5:**
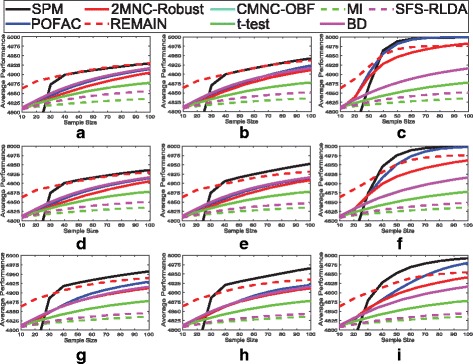
Average performance of feature selection algorithms. Average performance is obtained using 9 combinations of the synthetic microarray model parameters *k* and mean type with fixed *ρ*_0_ and *ρ*_1_, where performance is defined to be the average number of markers identified as good plus the average number of non-markers identified as bad over 500 iterations: **a** *ρ*_0_=0.1,*ρ*_1_=0.1, **b** *ρ*_0_=0.5,*ρ*_1_=0.1, **c** *ρ*_0_=0.9,*ρ*_1_=0.1, **d** *ρ*_0_=0.1,*ρ*_1_=0.5, **e** *ρ*_0_=0.5,*ρ*_1_=0.5, **f** *ρ*_0_=0.9,*ρ*_1_=0.5, **g** *ρ*_0_=0.1,*ρ*_1_=0.9, **h** *ρ*_0_=0.5,*ρ*_1_=0.9, and **i** *ρ*_0_=0.9,*ρ*_1_=0.9

Figure 5 plots average performance for fixed class-conditioned correlation coefficients across other distribution parameters. Simpler methods outperform complicated algorithms when sample size is small, and REMAIN enjoys outstanding performance for small sample sizes by reporting very few features. REMAIN has difficulty detecting weak markers, i.e., heterogeneous markers with class 1 mean close to 0, as for larger sample sizes its performance increment is very little for a 10 point increase in sample size. Average performance with respect to sample size for each of the 81 possible data generation settings is provided in the supplementary [see Additional file 1].

While correctly labeling more features tends to result in lower classification error, maximizing the average number of correctly labeled features does not necessarily minimize classification error [see Additional file 1]. An example can be seen in the Supplementary, where we examine the prediction error of several popular classifiers with feature selection on the synthetic microarray model [see Additional file 1].

## Results

We apply CMNC-OBF, POFAC, REMAIN, and SPM with the same priors used for synthetic microarray simulations to cancer microarray datasets, select the top genes, and perform enrichment analysis. We list the top 5 genes selected by CMNC-OBF, POFAC, and REMAIN. The top 100 genes are provided in the supplementary [see Additional file 1]. REMAIN ranks genes as follows. In each call to MAIN, we rank genes of $\tilde {G}$ by the order they are added to $\tilde {G}$, and if several genes are added at once in step 5 of MAIN, they are ranked based on $\tilde {\pi }^{*}(f)$. In addition, $\tilde {G}$’s are ranked by the order they are obtained using consecutive calls to the MAIN subroutine. Note SPM outputs a set of feature blocks, not a feature ranking. Studying blocks of SPM might provide invaluable information about the underlying biological mechanisms of the disease under study, but we leave this for future work.

We perform enrichment analysis using PANTHER [17, 18]. The top 20 enriched pathways are reported in the supplementary [see Additional file 1]. We list their names, number of known genes in each pathway, number of selected genes that belong to the pathway, and the corresponding *p*-value. Here we only list the top 3 pathways and their *p*-values. We study if among the top genes and pathways any are already suggested to be involved in the cancer under study. The complete analysis, with references that suggest involvement of the top reported genes and pathways involved in cancer, is provided in the supplementary [see Additional file 1]. Here, we only report the conclusions made in the supplementary based on our literature review [see Additional file 1].

CMNC-OBF tends to find individually strong genes, which are typically those already known to be involved in cancer. Hence, CMNC-OBF tends to give the best enrichment analysis results, but it might not be the best option to find biomarkers that are individually weak, but heavily correlated to strong biomarkers. POFAC and REMAIN tend to find genes that are individually strong or highly correlated to individually strong biomarkers. Hence, they might be very useful for many practical applications, particularly for those where it is desired to target genes directly involved in cancer, or genes directly interacting with them. SPM is specifically designed to find all genes correlated to individually strong biomarkers. Hence, it tends to report large gene sets. Thereby, this algorithm is particularly useful for identifying and hypothesizing which biological functions are affected in the cancer under study.

POFAC and CMNC-OBF require the user to specify the number of genes to select, which we fix to 2000 so that a reasonable number of genes are identified by the pathway enrichment analysis database. On the other hand, REMAIN and SPM cannot take a predetermined number of genes to select. We adjust their thresholds for each dataset so that a reasonable number of genes are selected. We fix *T*_2_=*n*, and tune *T*_1_,*t*_1_,*t*_2_, and *T*_4_.

The following process is used on each dataset. We first remove probes that are not mapped to any genes. We then use OBF and POFAC to rank probes, and use REMAIN to select a subset of probes. If multiple probes are mapped to the same genes, only the probe with the highest rank is retained. This gives the selected genes of REMAIN, and final gene rankings of OBF and POFAC. *D*=2000 is used to obtain gene sets of CMNC-OBF and POFAC. SPM uses the gene ranking obtained by POFAC with the corresponding $\tilde {\beta }(.)$, where among probes mapped to the same genes only the probe ranking highest is retained. Running all algorithms, using MATLAB2015b, on a server with 4 XEON E5-4650L processors and 512GB of RAM took about 20 minutes for the breast cancer dataset, and about 70 minutes for each of the colon cancer and AML datasets. For all datasets REMAIN and SPM took about 55% and 25% of the total run-time, respectively.

### Breast cancer

Data obtained in [19] is curated on Gene Expression Omnibus (GEO) [20] with accession number GSE1456, containing 159 points. 119 breast cancer relapse free patients comprise class 0 and 40 patients with breast cancer relapses comprise class 1. In this dataset, “the raw expression data were normalized using the global mean method” [19]. Feature selection algorithms pick the top genes, and enrichment analysis is performed using PANTHER. Here we implement REMAIN with *T*_1_=0.005, and obtain 1413 genes, and SPM with *T*_4_=1000*n*^2^,*t*_1_=1000, and *t*_2_=1, and obtain 101 blocks containing 1048 genes. Top genes and pathways are listed in Tables 3 and 4, respectively. PANTHER pathways recognize 358, 254, 328, and 183 of the genes selected by CMNC-OBF, REMAIN, POFAC, and SPM, respectively. Many of the top genes and pathways are suggested to be involved in breast cancer. For instance, PHTF1, ZNF192, and MUC5AC are already shown to be involved in breast cancer. Furthermore, DCT and ZP2 are high-profile biomarkers, and their role in breast cancer requires further investigation. Among pathways, the gonadotropin-releasing hormone receptor pathway, ubiquitin proteasome pathway, CCKR signaling map, and integrin signalling pathway are shown to be involved in breast cancer.

**Table 3:** Top genes of breast cancer

Rank	CMNC-OBF	REMAIN	POFAC
1	DCT	DCT	DCT
2	PHTF1	ZNF192	PHTF1
3	ZNF227	ZP2	MUC5AC
4	ZP2	PCSK6	HUWE1
5	CEACAM7	CEACAM7	MLANA

**Table 4:** Top pathways of breast cancer

Algorithm	Pathway	*P*-value
CMNC-OBF	Gonadotropin-releasing hormone receptor pathway	4.93*E*−05
	p53 pathway	8.34*E*−05
	Ubiquitin proteasome pathway	1.26*E*−04
REMAIN	Ubiquitin proteasome pathway	3.33*E*−07
	Angiogenesis	3.40*E*−04
	FAS signaling pathway	7.23*E*−04
POFAC	CCKR signaling map	3.82*E*−05
	p53 pathway	2.61*E*−04
	Ubiquitin proteasome pathway	4.18*E*−04
SPM	Ubiquitin proteasome pathway	6.25*E*−07
	Integrin signalling pathway	1.72*E*−06
	Pyrimidine Metabolism	2.87*E*−05

Due to different properties of these algorithms, different types of biomarkers they tend to pick, and our limited knowledge of cancer pathways, it is natural to obtain different gene sets, *p*-values, and pathway rankings. However, there is reasonable consistency between the enrichment analysis results. For instance, the ubiquitin proteasome pathway, which is in the top 3 pathways of all algorithms, is shown to be involved in breast cancer. Many of the top 20 pathways are in common between at least 3 algorithms and are shown to be involved in breast cancer. For instance, the gonadotropin-releasing hormone receptor pathway, FAS signaling pathway, P53 pathway, CCKR signaling map, de novo purine biosynthesis, TCA cycle, Cytoskeletal regulation by Rho GTPase, and cell cycle are involved in breast cancer. In addition, many of the top 20 genes are in common between algorithms ranking features. For instance, PHTF1, MUC5AC, ZNF192, PCSK6, and HDGFRP3 are shown to be involved in breast cancer, and some common genes such as DCT, ZP2, and CEACAM7 might be involved in breast cancer.

### Colon cancer

Data obtained in [21, 22] is curated on GEO [20] with accession number GSE17538, containing gene expression levels of 238 patients in stages 1-4 of colon cancer. Twenty eight stage 1 patients comprise class 0 and the remaining patients comprise class 1. Bioconductor’s affy package with its default settings has normalized the data. Feature selection algorithms pick the top genes, and enrichment analysis is performed using PANTHER. Here we implement REMAIN with *T*_1_=0.01 to obtain 1289 genes, and SPM with *T*_4_=10^7^*n*^4^, *t*_1_=10^6^, and *t*_2_=4, to obtain 159 blocks containing 1560 genes. Top genes and pathways are listed in Tables 5 and 6, respectively. PANTHER pathways recognize 312, 159, 208, and 174 of the genes selected by CMNC-OBF, REMAIN, POFAC, and SPM, respectively. Many of the top genes and pathways are suggested to be involved in colon cancer. For instance, CPNE4 and EPHA7 are already shown to be involved in colon cancer. Among pathways, the cadherin signaling pathway and ionotropic glutamate receptor pathway are shown to be involved in colon cancer.

**Table 5:** Top genes of colon cancer

Rank	CMNC-OBF	REMAIN	POFAC
1	CPNE4	EPHA7	EPHA7
2	GAGE1,12,4,5,6,7	NBLA00301	CPNE4
3	GAGE1,12,2,4,5,6,7,8	LOC100133920 LOC286297	LOC100133920 LOC286297
4	S100A7	PDK4	SCN7A
5	EPHA7	MYH11	NBLA00301

**Table 6:** Top pathways of colon cancer

Algorithm	Pathway	*P*-value
CMNC-OBF	Cadherin signaling pathway	1.83*E*−20
	Wnt signaling pathway	7.25*E*−14
	Plasminogen activating cascade	7.67*E*−05
REMAIN	Cadherin signaling pathway	2.21*E*−10
	Plasminogen activating cascade	7.95*E*−05
	Wnt signaling pathway	9.27*E*−05
POFAC	Ionotropic glutamate receptor pathway	1.72*E*−05
	Metabotropic glutamate receptor group III pathway	1.04*E*−02
	Nicotinic acetylcholine receptor signaling pathway	1.70*E*−02
SPM	Ionotropic glutamate receptor pathway	2.98*E*−03
	Heterotri. G-prot. sig. P.W., Gi alpha & Gs alpha med. P.W.	4.73*E*−03
	Axon guidance mediated by Slit/Robo	5.79*E*−03

In the supplementary (Additional file 1) we show: (1) CPNE4, EPHA7, and LOC286297, which are among the top 20 genes of all three algorithms that rank genes, are shown to be involved in colon cancer, (2) many of the top 20 genes in common between two of the gene ranking algorithms, such as the GAGE genes, RYR3, PDK4, and MYH11, are suggested to be involved in colon cancer, and (3) among the common top 20 enriched pathways, the plasminogen activating cascade, blood coagulation, and the beta1 adrenergic receptor signaling pathway are suggested to be involved in colon cancer.

### Acute myeloid leukemia

Data obtained in [23–25] is deposited on GEO with accession number GSE13204, containing gene expression levels of 2096 points. 74 points belong to healthy people, 542 points belong to Acute Myeloid Leukemia (AML) patients, and the remaining points are other subtypes of leukemia. Healthy points comprise class 0 and AML patients comprise class 1. The data is already pre-processed, including a summarization and quantile normalization step. Feature selection algorithms pick the top genes, and enrichment analysis is performed using PANTHER. Here we implement REMAIN with *T*_1_=0.05 to obtain 957 genes, and SPM with *T*_4_=10^7^*n*^10^, *t*_1_=10^6^, and *t*_2_=4, to obtain 522 blocks containing 5172 genes. Although the thresholds of SPM are chosen to be very large, we still pick very many genes. This might imply that many of the genes involved in AML might be individually weak, but highly correlated.

Top genes and pathways are listed in Tables 7 and 8, respectively. PANTHER pathways recognize 276, 141, 266, and 671 of the genes selected by CMNC-OBF, REMAIN, POFAC, and SPM, respectively. Many of the top genes and pathways are suggested to be involved in AML. For instance, ORM1 and ORM2 are already shown to be involved in AML, LTF is a high-profile gene whose role in AML needs further investigation, and S100A12 is shown to be involved in similar subtypes of leukemia, such as ALL, and is suggested to be involved in AML as well. Among pathways, heme biosynthesis, the interferon-gamma signaling pathway, pentose phosphate pathway and ubiquitin proteasome pathway have suggested involvement in AML.

**Table 7:** Top genes of AML

Rank	CMNC-OBF	REMAIN	POFAC
1	ORM1 ORM2	LTF	S100A12
2	LTF	CRISP3	S100A9
3	CRISP3	ORM1 ORM2	ORM1 ORM2
4	CHIT1	CHIT1	CRISP3
5	DNAH10	DNAH10	LTF

**Table 8:** Top pathways of AML

Algorithm	Pathway	*P*-value
CMNC-OBF	Heme biosynthesis	2.78*E*−04
	Pentose phosphate pathway	7.34*E*−03
	De novo purine biosynthesis	8.40*E*−03
REMAIN	Interferon-gamma signaling pathway	5.86*E*−04
	Alzheimer disease-presenilin pathway	6.11*E*−03
	Inflammation med. by chemokine & cytokine sig. P.W.	6.22*E*−03
POFAC	Pentose phosphate pathway	1.15*E*−03
	Formyltetrahydroformate biosynthesis	1.15*E*−03
	Heme biosynthesis	1.77*E*−03
SPM	Ubiquitin proteasome pathway	1.72*E*−08
	T cell activation	2.07*E*−05
	Inflammation med. by chemokine & cytokine sig. P.W.	3.00*E*−05

Studying the top 20 genes and pathways in the supplementary (Additional file 1) we see that ORM1, ORM2, LTF, CAMP, LCN2, MMP9, CYP4F3, WT1, and CRISP3 are among the top 20 genes of all gene ranking algorithms, and are shown or suggested to be involved in AML. Among the top pathways in common between all methods, the interferon signaling pathway, and the inflammation mediated by chemokine and cytokine signaling pathway are involved in AML. Many of the top pathways picked by at least 3 methods, such as heme biosynthesis, denovo purine biosynthesis, and T-cell activation are also suggested to be involved in AML.

## Discussion

Here we proposed several suboptimal feature selection algorithms outperforming many popular algorithms. However, the ability to correctly detect weaker biomarkers via these suboptimal methods comes at the expense of less intuitive objective functions compared with the optimal solutions. Although the proposed algorithms are more computationally intensive than OBF and 2MNC-Robust, they are still much faster than many popular feature selection algorithms.

While the previously introduced OBF is suitable to find individually strong biomarkers, REMAIN and POFAC find individually strong biomarkers as well as individually weak biomarkers heavily correlated to strong biomarkers, which is useful for many practical applications. For instance, in drug development it might be desirable to target genes directly involved in biological mechanisms of the cancer under study, or target genes strongly correlated to them to indirectly control the behavior of genes directly involved in cancer.

When sample size is small or correlations are not very strong one could use REMAIN to find a small set of high-profile biomarkers. REMAIN cannot be forced to output a predetermined number of features, but for a given fixed dataset its parameters can be tuned to output close to a desired number of features. Note the output of REMAIN is a feature ranking that greatly depends on *T*_1_. The larger *T*_1_ is, the smaller is the output feature ranking. However, the user would have more confidence that declared features are biomarkers. While *π*^∗^(*f*) is a very intuitive quantity to evaluate the quality of a feature, $\tilde {\pi }^{*}(f)$ obtained by finding $\tilde {\pi }^{*}(G)$ for sets of size 2 is not as easy to work with.

On the other hand, if sample size is large or correlations are strong, POFAC is a suitable option. POFAC provides a feature ranking based on $\tilde {\beta }(f)$ and the user specifies how many features to select, similar to CMNC-OBF and 2MNC-Robust. However, $\tilde {\beta }(f)$ is not as intuitive as *π*^∗^(*f*).

SPM outputs a family of good blocks, which is very useful in studying the interactions between biomarkers, and is very useful to hypothesize about biological mechanisms that are involved in the disease under study. As SPM is designed to pick all biomarkers correlated to strong ones, it typically reports larger feature sets. SPM is very desirable when correlations are large; however, it should only be used when sample size is relatively large. Finally, parameters of SPM can be used to adjust the trade-off between the size of detected blocks, and the minimum desired dependence between biomarkers. However, one cannot intuitively determine what values should be used to achieve a certain point in the trade-off. Trial-and-error can be used on a fixed sample to find the desired parameters, as we did in this paper. One of the main reasons it is not easy to predetermine SPM parameters is their dependence on sample size and the underlying distribution parameters.

## Conclusion

The proposed Bayesian framework is indeed promising for biomarker discovery applications. The objective of finding the posterior probability that a feature set is the set of good features does not suffer many of the drawbacks of heuristics used in biomarker discovery. For instance, while t-test only captures differences in the means, OBF can capture differences in variances, 2MNC-Robust, REMAIN, and POFAC take pairwise dependencies into account, and SPM looks at the joint distribution of good blocks. While the proposed suboptimal methods can efficiently take advantage of dependencies to find the set of good features, many heuristics proposed to consider dependencies do not perform well under small samples [13], which is observed in our simulations as well.

While the optimal solution under the general block model is computationally infeasible, the success of proposed suboptimal algorithms shows the Bayesian framework can serve as a foundation to model biomarker discovery problems and develop efficient suboptimal methods. Future work includes studying the properties of the proposed algorithms, for instance their asymptotic properties, further analyzing outputs of the proposed algorithms on real datasets, and exploring the specific applications suitable for each algorithm in greater detail. In addition, prior construction may be used to design prior distributions for SPM to boost its performance under small samples. Finally, while here we have studied SPM’s ability to select all relevant features, it actually outputs blocks of features that appear highly correlated and differentially expressed. In future work we will examine SPM as a block detection algorithm, which has important applications in gene network modeling.

## Additional file

Additional file 1 Supplementary. Additional detail on the synthetic simulations and a comparison of classification error of the selected features of each algorithm is provided. The supplementary contains the top 100 selected genes and top 20 enriched pathways of each of the proposed algorithms as well as limma. (PDF 507 kb)
