# Supplementary material for: Heuristic algorithms for feature selection under Bayesian models with block-diagonal covariance structure
Source: BMC Bioinformatics. 2018 Mar 21;19(Suppl 3):70. doi: 10.1186/s12859-018-2059-8 (PMC5872553; doi:10.1186/s12859-018-2059-8)
Supplement: Supplementary file 1 — Supplementary. Additional detail on the synthetic simulations and a comparison of classification error of the selected features of each algorithm is provided. The supplementary contains the top 100 selected genes and top 20 enriched pathways of each of the proposed algorithms as well as limma. (PDF 507 kb) [file 12859_2018_2059_MOESM1_ESM.pdf]

RESEARCH

Heuristic algorithms for feature selection under Bayesian models with block-diagonal covariance structure: Supplementary

Ali Foroughi pour[1*] and Lori A Dalton[1,2]

*Correspondence: foroughipour.1@osu.edu
[1]Department of Electrical and Computer Engineering, The Ohio State University, 2015 Neil Avenue, 43210 Columbus, USA
Full list of author information is available at the end of the article

1 Synthetic Microarray Simulation

Here we include the figures of average number of correctly labeled features versus sample size of all feature selection algorithms used in the simulation of section 4.2 for each set of distribution parameters, which are the mean type, block size, ρ_0, and ρ_1. Figs. 1 to 9 plot the results. Each figure contains all 9 combinations of ρ_0 and ρ_1 for a fixed mean type and block size.

2 Classification on Synthetic Microarray Data

Here we use the synthetic microarray model with block size $k = 5$, and consider all means types and correlations of section 4.2. Given the selected features of a feature selection algorithm, we train the following classifiers: Regularized Quadratic Discriminant Analysis (RQDA), Diagonal Quadratic Discriminant Analysis (DQDA), Regularized Linear Discriminant Analysis (RLDA), Diagonal Linear Discriminant Analysis (DLDA), a linear Support Vector Machine (SVM), a quadratic SVM, an SVM with Radial Basis Function (RBF) kernel, a Generalized Linear Model (GLM) with logit link, and a GLM with probit link. We generate 1000 points from each class, and test the trained classifiers on generated test data. We iterate 500 times to find the classification error of each classifier. For each feature selection algorithm we assign the minimum error of all constructed classifiers as the error of that algorithm. Figures 10 to 12 plot the error of classifiers built using feature selection algorithms of section 4.2. As the results indicate, typically methods that perform better on feature selection yield lower classification error. However, there are few exceptions. For instance, when markers are marginal and $\rho_0 = \rho_1 = 0.1$, POFAC slightly outperforms 2MNC-Robust in features selection, but has higher classification error. Furthermore, as long as sample size is larger than 30, proposed algorithms yield lower classification error compared with other feature selection algorithms.

3 Real Datasets

Here we present the top 100 genes of CMNC-OBF, REMAIN, and POFAC on the cancer datasets. We also present the top 20 pathways of CMNC-OBF, REMAIN, POFAC, and SPM, and study if the top genes and pathways are already shown or suggested to be involved in the cancer under study. We also find potential biomarkers which their role in cancer requires further investigation.

We also report the results of linear models for microarray and RNA-Seq data (limma) on these real datasets. In order to find the differentially expressed genes

we fit a linear model with design matrix X comprised of two columns, where each column is the indicator of sample points belonging to a class. We also consider the contrast matrix C to be [1, −1], differentiating the mean between two classes. We then find the adjusted p-values using moderated t-statistic obtained by the empirical Bayes approach described in [1]. We pick the top 2000 genes as well as significant genes bounding FDR by 0.05 using Benjamini-Hochberg procedure [2]. We report the top 100 genes, and top enriched pathways of PANTHER for both top 2000 genes and significant genes.

3.1 Breast Cancer Dataset

This dataset is curated on GEO with accession number GSE1456, containing 159 points. 119 breast cancer relapse free patients comprise class 0 and 40 patients with breast cancer relapses comprise class 1. The top 100 genes of REMAIN, CMNC-OBF, and POFAC are provided in Tabs. 1, 2 and 3, respectively. The top 20 pathways of CMNC-OBF, REMAIN, POFAC, and SPM are listed in Tabs. 5, 6, 7, and 8, respectively.

For CMNC-OBF, the top pathway, gonadotropin-releasing hormone receptor pathway [3, 4, 5], is suggested to be involved in breast cancer, as well as the second top gene, PHTF1 [6, 7, 8, 9].

For REMIAN with $T_1 = 0.005$, the top pathway, ubiquitin proteasome pathway, is suggested to be involved in breast cancer [10], and the second top gene, ZNF192, is suggested to be involved in metastatic progression of breast cancer [11].

Using POFAC to do enrichment analysis, the top pathway, CCKR signaling map [12], is suggested to be involved in breast cancer, as well as the second and third top genes, PHTF1 [6, 7, 8, 9] and MUC5AC [13, 14].

Using genes detected by SPM with $T_4 = 1000n^2$, $t_1 = 1000$ and $t_2 = 1$, and performing enrichment analysis, the top pathway, integrin signaling pathway, is shown to be involved in breast cancer [15, 16, 17, 18].

Among the top 20 pathways selected by all 4 algorithms, ubiquitin proteasome pathway, which is among the top 3 pathways of all algorithms, is shown to be involved in breast cancer [10]. Also, many other pathways are in common between at least 3 of these algorithms, which are also shown to be involved in breast cancer, for instance, gonadotropin-releasing hormone receptor pathway [3, 4, 5], FAS signaling pathway[19, 20], P53 pathway [21], CCKR signaling map [12, 22], de novo purine biosynthesis [23, 24], TCA cycle [25, 26], Cytoskeletal regulation by Rho GTPase [27, 28], and cell cycle [29, 30] . Also, many of the top 20 genes are in common between algorithms ranking features such as PHTF1 [6, 7, 8, 9], MUC5AC [13, 14], ZNF192 [11], PCSK6 [31, 32, 33], HDGFRP3 [34, 35, 36], which are shown to be involved in breast cancer. ZP2, and CEACAM7, are also selected by gene ranking algorithms, and might be involved in breast cancer. CEACAM7 might be involved in breast cancer [37]. Also, it can be inferred that ZP2 might be involved in breast cancer [38]. Note that PAX3 is involved in breast cancer [39], and represses DCT [40]. Hence, DCT is a high-profile gene which might be involved in breast cancer. However, its role in breast cancer requires further investigation.

Finally, we report the results of limma on this dataset. Tab. 4 lists the top 100 genes, and Tabs. 9 and 10 report the enriched pathways for the top 2000 genes,

and significant genes, respectively. Note using top 2000 genes and the 782 unique significant genes, PANTHER pathways recognize 350 and 157 genes, respectively.

Many of the top genes selected by the proposed algorithms were not among the top 2000 genes of limma. For instance, DCT, PHTF1, MUCA5C, ZNF227, ZP2, and CEACAM7 are not selected by limma. On the other hand, many of the top genes of limma are among the top 100 genes of the proposed algorithms. For instance, the top gene of limma, CENPF, is among the top 100 genes of CMNC-OBF, POFAC, and REMAIN, or the second and fourth top genes of limma, KIAA0101 and H2AFZ, are selected by CMNC-OBF and REMAIN. Furthermore, the third top gene of limma, PDGFD, is selected by CMNC-OBF.

3.2 Colon Cancer Dataset

This dataset is curated on GEO with accession number GSE41850, containing gene expression levels of 238 patients in stages 1-4 of colon cancer. 28 stage 1 patients comprise class 0 and the remaining patients comprise class 1. The top 100 genes of REMAIN, CMNC-OBF, and POFAC are provided in Tabs. 11, 12 and 13, respectively. The top 20 pathways of CMNC-OBF, REMAIN, POFAC, and SPM are listed in Tabs. 15, 16, 17, and 18, respectively.

We again start by CMNC-OBF. The top pathway, cadherin signaling pathway [41, 42], and the top gene, CPNE4 [43], are suggested to be involved in colon cancer.

Now we look at enrichment analysis of REMAIN with $T_1 = 0.01$. The top pathway, cadherin signaling pathway [41, 42], and the top gene, EPHA7 [44, 45], are suggested to be involved in colon cancer.

Now we use POFAC gene set to do enrichment analysis. The top pathway, ionotropic glutamate receptor pathway [46, 47], is suggested to be involved in colon cancer, as well as the top two genes, EPHA7 [44, 45], and CPNE4 [43].

Using genes detected by SPM with $T_4 = 10^7 n^4$, $t_1 = 10^6$ and $t_2 = 4$, we performed enrichment analysis. The top pathway, ionotropic glutamate receptor pathway, is shown to be involved in colon cancer [46, 47].

We also find many top genes and pathways selected by several algorithms among their top 20 genes and pathways. For instance, CPNE4 [43], EPHA7 [44, 45], LOC286297 [48] and LOC100133920 are selected by all 3 algorithms ranking genes, where the first 3 genes are shown to be involved in colon cancer. In addition, many of the genes selected by 2 of the gene ranking algorithms, such as GAGE genes [49], RYR3 [50], PDK4 [51, 52], and MYH11 [53, 54] are suggested to be involved in colon cancer. Among the top pathways, plasminogen activating cascade [55, 56], blood coagulation [57], and beta1 adrenergic receptor signaling pathway [58, 59], are all suggested to be involved in colon cancer.

Finally, we report the results of limma on this dataset. Tab. 14 lists the top 100 genes, and Tabs. 19 and 20 report the enriched pathways for the top 2000 genes, and significant genes, respectively. Note using top 2000 genes and the 194 unique significant genes, PANTHER pathways recognize 333 and 44 genes, respectively.

While some top genes are shared between limma and other Bayesian methods, such as SCN7A, many of the top genes of Bayesian methods, such as EPHA7 and SPNE4, are not among the top 2000 genes of limma. On the other hand, many top genes of limma are among the top 100 genes of the proposed Bayesian algorithms.

For instance, the top 4 genes of limma are also selected by CMNC-OBF, and the fifth top gene, GPM6A, is among the top genes of CMCN-OBF, REMAIN, and POFAC.

3.3 AML

This dataset is curated on GEO with accession number GSE1456, containing 2096 points. 74 points corresponding to healthy people comprise class 0 and 542 points corresponding to AML patients comprise class 1. The top 100 genes of REMAIN, CMNC-OBF, and POFAC are provided in Tabs. 21, 22 and 23, respectively. The top 20 pathways of CMNC-OBF, REMAIN, POFAC, and SPM are listed in Tabs. 25, 26, 27, and 28, respectively.

We start with CMNC-OBF and perform enrichment analysis. The top pathway, heme biosynthesis, [60, 61, 62] and the top genes, ORM1 [63, 64] and ORM2 [65], are shown to be involved in AML.

We use the gene set of REMAIN with $T_1 = 0.05$ to perform enrichment analysis. The top pathway, interferon-gamma signaling pathway, is suggested to be involved in AML [66, 67]. The top gene, LTF, might be involved in AML [68].

Now we use POFAC gene set to perform enrichment analysis. The top pathway, pentose phosphate pathway [69, 70], is suggested to be involved in AML. The top gene, S100A12, is shown to be involved in similar subtypes of leukemia, such as ALL, and is suggested to be involved in AML as well [71, 72, 73].

We use SPM with thresholds $T_4 = 10^7 n^{10}$, $t_1 = 10^6$, and $t_2 = 4$ to find biomarkers and perform enrichment analysis. The top pathway, ubiquitin proteasome pathway is involved in AML [74, 75].

Among the top 20 genes and pathways, we again find many top genes and pathways selected by several algorithms. For instance, all algorithms ranking genes pick ORM1 [63, 64], ORM2 [65], LTF [68], CAMP [76, 77, 78], LCN2 [64, 79], MMP9 [80, 81], CYP4F3 [82], and WT1 [83] are shown or suggested to be involved in AML. Also, CRISP3 [84, 85, 86, 87, 88] has been suggested to be involved in AML and other subtypes of leukemia. Among the top pathways in common between all 4 methods, interferon signaling pathway [67, 89] and inflammation mediated by chemokine and cytokine signaling pathway [90, 91] are shown to be involved in AML. In addition, many of the top pathways picked by 3 methods, such as heme biosynthesis [60, 61, 62], denovo purine biosynthesis [92, 93], and T-cell activation [94, 95, 96] are also shown to be involved in AML.

Finally, we report the results of limma on this dataset. Tab. 24 lists the top 100 genes, and Tab. 29 reports the enriched pathways of the top 2000 genes, for which PANTHER pathways recognize 159 genes. Note bounding FDR by 0.05 we found 2001 significant genes, where the gene ranking 2001 was not recognized by PANTHER pathways, and hence did not improve enrichment results. Note on this data set, in contrast to previous examples, many of the top genes of limma and Bayesian methods are in common. For instance, CRISP3, MMP9, LCN2, ORM1, and WT1 are selected by limma as well as the proposed algorithms.

Author details

[1]Department of Electrical and Computer Engineering, The Ohio State University, 2015 Neil Avenue, 43210 Columbus, USA. [2]Department of Biomedical Informatics,The Ohio State University, 250 Lincoln Tower, 1800 Cannon Drive, 43210 Columbus, USA.

References

1. Smyth, G.K.: Linear models and empirical bayes methods for assessing differential expression in microarray experiments. Statistical applications in genetics and molecular biology **3**(1), 1–25 (2004)
2. Benjamini, Y., Hochberg, Y.: Controlling the false discovery rate: a practical and powerful approach to multiple testing. Journal of the royal statistical society. Series B (Methodological), 289–300 (1995)
3. Winer, E.P., Hudis, C., Burstein, H.J., Wolff, A.C., Pritchard, K.I., Ingle, J.N., Chlebowski, R.T., Gelber, R., Edge, S.B., Gralow, J., Cobleigh, M.A., Mamounas, E.P., Goldstein, L.J., Whelan, T.J., Powles, T.J., Bryant, J., Perkins, C., Perotti, J., Braun, S., Langer, A.S., Browman, G.P., Somerfield, M.R.: American society of clinical oncology technology assessment on the use of aromatase inhibitors as adjuvant therapy for postmenopausal women with hormone receptor–positive breast cancer: status report 2004. Journal of Clinical Oncology **23**(3), 619–629 (2005)
4. Eidne, K.A., Flanagan, C.A., Millar, R.P.: Gonadotropin-releasing hormone binding sites in human breast carcinoma. Science **229**, 989–992 (1985)
5. Del Mastro, L., Boni, L., Michelotti, A., Gamucci, T., Olmeo, N., Gori, S., Giordano, M., Garrone, O., Pronzato, P., Bighin, C., Levaggi, A., Giraudi, S., Cresti, N., Magnolfi, E., Scotto, T., Vecchio, C., Venturini, M.: Effect of the gonadotropin-releasing hormone analogue triptorelin on the occurrence of chemotherapy-induced early menopause in premenopausal women with breast cancer: a randomized trial. JAMA **306**(3), 269–276 (2011)
6. Castro, N.P., Osório, C.A., Torres, C., Bastos, E.P., Mourão-Neto, M., Soares, F.A., Brentani, H.P., Carraro, D.M.: Evidence that molecular changes in cells occur before morphological alterations during the progression of breast ductal carcinoma. Breast Cancer Research **10**(5), 87 (2008)
7. Fan, M., Sethuraman, A., Brown, M., Sun, W., Pfeffer, L.M.: Systematic analysis of metastasis-associated genes identifies mir-17-5p as a metastatic suppressor of basal-like breast cancer. Breast Cancer Research and Treatment **146**(3), 487–502 (2014)
8. Amaya, C., Kurisetty, V., Stiles, J., Nyakeriga, A.M., Arumugam, A., Lakshmanaswamy, R., Botez, C.E., Mitchell, D.C., Bryan, B.A.: A genomics approach to identify susceptibilities of breast cancer cells to "fever-range" hyperthermia. BMC Cancer **14**(1), 81 (2014)
9. Natrajan, R., Williams, R.D., Grigoriadis, A., Mackay, A., Fenwick, K., Ashworth, A., Dome, J.S., Grundy, P.E., Pritchard-Jones, K., Jones, C.: Delineation of a 1mb breakpoint region at 1p13 in wilms tumors by fine-tiling oligonucleotide array cgh. Genes, Chromosomes and Cancer **46**(6), 607–615 (2007)
10. Orlowski, R.Z., Dees, E.C.: The role of the ubiquitination-proteasome pathway in breast cancer: applying drugs that affect the ubiquitin-proteasome pathway to the therapy of breast cancer. Breast Cancer Research **5**(1), 1–7 (2002)
11. Addison, J.B., Koontz, C., Fugett, J.H., Creighton, C.J., Chen, D., Farrugia, M.K., Padon, R.R., Voronkova, M.A., McLaughlin, S.L., Livengood, R.H., Lin, C.-C., Ruppert, J.M., Pugacheva, E.N., Ivanov, A.V.: Kap1 promotes proliferation and metastatic progression of breast cancer cells. Cancer Research **75**(2), 344–355 (2015)
12. Reubi, J.C., Schaer, J.-C., Waser, B.: Cholecystokinin(cck)-a and cck-b/gastrin receptors in human tumors. Cancer Research **57**(7), 1377–1386 (1997)
13. Rakha, E.A., Boyce, R.W.G., El-Rehim, D.A., Kurien, T., Green, A.R., Paish, E.C., Robertson, J.F.R., Ellis, I.O.: Expression of mucins (muc1, muc2, muc3, muc4, muc5ac and muc6) and their prognostic significance in human breast cancer. Modern Pathology **18**(10), 1295–1304 (2005)
14. Matsukita, S., Nomoto, M., Kitajima, S., Tanaka, S., Goto, M., Irimura, T., Kim, Y.S., Sato, E., Yonezawa, S.: Expression of mucins (muc1, muc2, muc5ac and muc6) in mucinous carcinoma of the breast: comparison with invasive ductal carcinoma. Histopathology **42**(1), 26–36 (2003)
15. Felding-Habermann, B., O'Toole, T.E., Smith, J.W., Fransvea, E., Ruggeri, Z.M., Ginsberg, M.H., Hughes, P.E., Pampori, N., Shattil, S.J., Saven, A., Mueller, B.M.: Integrin activation controls metastasis in human breast cancer. Proceedings of the National Academy of Sciences **98**(4), 1853–1858 (2001)
16. Gasparini, G., Brooks, P.C., Biganzoli, E., Vermeulen, P.B., Bonoldi, E., Dirix, L.Y., Ranieri, G., Miceli, R., Cheresh, D.A.: Vascular integrin alpha(v)beta3: a new prognostic indicator in breast cancer. Clinical Cancer Research **4**(11), 2625–2634 (1998)
17. White, D.E., Kurpios, N.A., Zuo, D., Hassell, J.A., Blaess, S., Mueller, U., Muller, W.J.: Targeted disruption of β1-integrin in a transgenic mouse model of human breast cancer reveals an essential role in mammary tumor induction. Cancer Cell **6**(2), 159–170 (2004)
18. Park, C.C., Zhang, H., Pallavicini, M., Gray, J.W., Baehner, F., Park, C.J., Bissell, M.J.: β1 integrin inhibitory antibody induces apoptosis of breast cancer cells, inhibits growth, and distinguishes malignant from normal phenotype in three dimensional cultures and in vivo. Cancer Research **66**(3), 1526–1535 (2006)
19. Kuhajda, F.P.: Fatty acid synthase and cancer: new application of an old pathway. Cancer Research **66**(12), 5977–5980 (2006)
20. Turley, J.M., Fu, T., Ruscetti, F.W., Mikovits, J.A., Bertolette, D.C., Birchenall-Roberts, M.C.: Vitamin e succinate induces fas-mediated apoptosis in estrogen receptor-negative human breast cancer cells. Cancer Research **57**(5), 881–890 (1997)
21. Bartek, J., Iggo, R., Gannon, J., Lane, D.P.: Genetic and immunochemical analysis of mutant p53 in human breast cancer cell lines. Oncogene **5**(6), 893–899 (1990)
22. Gajbhiye, A., Dabhi, R., Taunk, K., Vannuruswamy, G., RoyChoudhury, S., Adhav, R., Seal, S., Mane, A., Bayatigeri, S., Santra, M.K., Chaudhury, K., Rapole, S.: Urinary proteome alterations in her2 enriched breast cancer revealed by multipronged quantitative proteomics. PROTEOMICS **16**(17), 2403–2418 (2016)
23. Allegra, C.J., Hoang, K., Yeh, G.C., Drake, J.C., Baram, J.: Evidence for direct inhibition of de novo purine synthesis in human mcf-7 breast cells as a principal mode of metabolic inhibition by methotrexate. Journal of Biological Chemistry **262**(28), 13520–13526 (1987)
24. Jain, M., Nilsson, R., Sharma, S., Madhusudhan, N., Kitami, T., Souza, A.L., Kafri, R., Kirschner, M.W., Clish, C.B., Mootha, V.K.: Metabolite profiling identifies a key role for glycine in rapid cancer cell proliferation.

Science **336**(6084), 1040–1044 (2012)
25. Eichner, L.J., Perry, M.-C., Dufour, C.R., Bertos, N., Park, M., St-Pierre, J., Giguère, V.: mir-378 mediates metabolic shift in breast cancer cells via the pgc-1β/errγ transcriptional pathway. Cell Metabolism **12**(4), 352–361 (2010)
26. Chen, E.I., Hewel, J., Krueger, J.S., Tiraby, C., Weber, M.R., Kralli, A., Becker, K., Yates, J.R., Felding-Habermann, B.: Adaptation of energy metabolism in breast cancer brain metastases. Cancer Research **67**(4), 1472–1486 (2007)
27. Tilghman, S.L., Townley, I., Zhong, Q., Carriere, P.P., Zou, J., Llopis, S.D., Preyan, L.C., Williams, C.C., Skripnikova, E., Bratton, M.R., Zhang, Q., Wang, G.: Proteomic signatures of acquired letrozole resistance in breast cancer: suppressed estrogen signaling and increased cell motility and invasiveness. Molecular & Cellular Proteomics **12**(9), 2440–2455 (2013)
28. Ellenbroek, S.I.J., Collard, J.G.: Rho gtpases: functions and association with cancer. Clinical & Experimental Metastasis **24**(8), 657–672 (2007)
29. Porter, P.L., Malone, K.E., Heagerty, P.J., Alexander, G.M., Gatti, L.A., Firpo, E.J., Daling, J.R., Roberts, J.M.: Expression of cell-cycle regulators p27kip1 and cyclin e, alone and in combination, correlate with survival in young breast cancer patients. Nature Medicine **3**(2), 222–225 (1997)
30. Paruthiyil, S., Parmar, H., Kerekatte, V., Cunha, G.R., Firestone, G.L., Leitman, D.C.: Estrogen receptor β inhibits human breast cancer cell proliferation and tumor formation by causing a g2 cell cycle arrest. Cancer Research **64**(1), 423–428 (2004)
31. Venables, J.P., Klinck, R., Bramard, A., Inkel, L., Dufresne-Martin, G., Koh, C., Gervais-Bird, J., Lapointe, E., Froehlich, U., Durand, M., Gendron, D., Brosseau, J.-P., Thibault, P., Lucier, J.-F., Tremblay, K., Prinos, P., Wellinger, R.J., Chabot, B., Rancourt, C., Elela, S.A.: Identification of alternative splicing markers for breast cancer. Cancer Research **68**(22), 9525–9531 (2008)
32. Terasaka, S., Inoue, A., Tanji, M., Kiyama, R.: Expression profiling of estrogen-responsive genes in breast cancer cells treated with alkylphenols, chlorinated phenols, parabens, or bis- and benzoylphenols for evaluation of estrogenic activity. Toxicology Letters **163**(2), 130–141 (2006)
33. Turashvili, G., Bouchal, J., Baumforth, K., Wei, W., Dziechciarkova, M., Ehrmann, J., Klein, J., Fridman, E., Skarda, J., Srovnal, J., Hajduch, M., Murray, P., Kolar, Z.: Novel markers for differentiation of lobular and ductal invasive breast carcinomas by laser microdissection and microarray analysis. BMC Cancer **7**(1), 55 (2007)
34. Abba, M.C., Hu, Y., Sun, H., Drake, J.A., Gaddis, S., Baggerly, K., Sahin, A., Aldaz, C.M.: Gene expression signature of estrogen receptor α status in breast cancer. BMC Genomics **6**(1), 37 (2005)
35. Garcia, T., Jackson, A., Bachelier, R., Clément-Lacroix, P., Baron, R., Clézardin, P., Pujuguet, P.: A convenient clinically relevant model of human breast cancer bone metastasis. Clinical & Experimental Metastasis **25**(1), 33–42 (2008)
36. Karagoz, K., Sinha, R., Arga, K.Y.: Triple negative breast cancer: a multi-omics network discovery strategy for candidate targets and driving pathways. Omics: A Journal Of Integrative Biology **19**(2), 115–130 (2015)
37. Nishikawa, N., Toyota, M., Suzuki, H., Honma, T., Fujikane, T., Ohmura, T., Nishidate, T., Ohe-Toyota, M., Maruyama, R., Sonoda, T., Sasaki, Y., Urano, T., Imai, K., Hirata, K., Tokino, T.: Gene amplification and overexpression of prdm14 in breast cancers. Cancer Research **67**(20), 9649–9657 (2007)
38. Assou, S., Cerecedo, D., Tondeur, S., Pantesco, V., Hovatta, O., Klein, B., Hamamah, S., De Vos, J.: A gene expression signature shared by human mature oocytes and embryonic stem cells. BMC Genomics **10**(1), 10 (2009)
39. Stephens, P.J., McBride, D.J., Lin, M.-L., Varela, I., Pleasance, E.D., Simpson, J.T., Stebbings, L.A., Leroy, C., Edkins, S., Mudie, L.J., Greenman, C.D., Jia, M., Latimer, C., Teague, J.W., Lau, K.W., Burton, J., Quail, M.A., Swerdlow, H., Churcher, C., Natrajan, R., Sieuwerts, A.M., Martens, J.W.M., Silver, D.P., Langerød, A., Russnes, H.E.G., Foekens, J.A., Reis-Filho, J.S., Veer, L.v., Richardson, A.L., Børresen-Dale, A.-L., Campbell, P.J., Futreal, P.A., Stratton, M.R.: Complex landscapes of somatic rearrangement in human breast cancer genomes. Nature **462**(7276), 1005–1010 (2009)
40. Robson, E.J.D., He, S.-J., Eccles, M.R.: A panorama of PAX genes in cancer and development. Nature Reviews Cancer **6**(1), 52–62 (2006)
41. Avizienyte, E., Wyke, A.W., Jones, R.J., McLean, G.W., Westhoff, M.A., Brunton, V.G., Frame, M.C.: Src-induced de-regulation of e-cadherin in colon cancer cells requires integrin signalling. Nature Cell Biology **4**(8), 632–638 (2002)
42. Peña, C., García, J.M., Silva, J., García, V., Rodríguez, R., Alonso, I., Millán, I., Salas, C., de Herreros, A.G., Muñoz, A., Bonilla, F.: E-cadherin and vitamin d receptor regulation by snail and zeb1 in colon cancer: clinicopathological correlations. Human Molecular Genetics **14**(22), 3361–3370 (2005)
43. Shin, S., Cha, H.J., Lee, E.-M., Jung, J.H., Lee, S.-J., Park, I.-C., Jin, Y.-W., An, S.: Micrornas are significantly influenced by p53 and radiation in hct116 human colon carcinoma cells. International Journal of Oncology **34**(6), 1645 (2009)
44. Wang, J., Kataoka, H., Suzuki, M., Sato, N., Nakamura, R., Tao, H., Maruyama, K., Isogaki, J., Kanaoka, S., Ihara, M., Tanaka, M., Kanamori, M., Nakamura, T., Shinmura, K., Sugimura, H.: Downregulation of epha7 by hypermethylation in colorectal cancer. Oncogene **24**(36), 5637–5647 (2005)
45. Kim, M.S., Lee, J., Sidransky, D.: Dna methylation markers in colorectal cancer. Cancer and Metastasis Reviews **29**(1), 181–206 (2010)
46. Rzeski, W., Turski, L., Ikonomidou, C.: Glutamate antagonists limit tumor growth. Proceedings of the National Academy of Sciences **98**(11), 6372–6377 (2001)
47. Julio-Pieper, M., Flor, P.J., Dinan, T.G., Cryan, J.F.: Exciting times beyond the brain: metabotropic glutamate receptors in peripheral and non-neural tissues. Pharmacological Reviews **63**(1), 35–58 (2011)
48. Hu, H., Shu, M., He, L., Yu, X., Liu, X., Lu, Y., Chen, Y., Miao, X., Chen, X.: Epigenomic landscape of 5-hydroxymethylcytosine reveals its transcriptional regulation of lncrnas in colorectal cancer. British Journal of Cancer **116**(5), 658–668 (2017)

49. Novikov, D.V., Belova, T.V., Plekhanova, E.S., Yanchenko, O.S., Novikov, V.V.: Early detection of cancer/testis mrnas in tumor cells circulating in the peripheral blood of colorectal cancer patients. Molecular Biology **46**(5), 687–692 (2012)
50. Chae, Y.S., Kim, J.G., Kang, B.W., Lee, S.J., Lee, Y.J., Park, J.S., Choi, G.S., Lee, W.K., Jeon, H.-S.: Functional polymorphism in the microrna-367 binding site as a prognostic factor for colonic cancer. Anticancer Research **33**(2), 513–519 (2013)
51. Bush, C.R., Havens, J.M., Necela, B.M., Su, W., Chen, L., Yanagisawa, M., Anastasiadis, P.Z., Guerra, R., Luxon, B.A., Thompson, E.A.: Functional genomic analysis reveals cross-talk between peroxisome proliferator-activated receptor γ and calcium signaling in human colorectal cancer cells. Journal of Biological Chemistry **282**(32), 23387–23401 (2007)
52. Van Erk, M.J., Roepman, P., Van der Lende, T.R., Stierum, R.H., Aarts, J.M.M.J.G., Van Bladeren, P.J., Van Ommen, B.: Integrated assessment by multiple gene expression analysis of quercetin bioactivity on anticancer–related mechanisms in colon cancer cells in vitro. European Journal of Nutrition **44**(3), 143–156 (2005)
53. Alhopuro, P., Sammalkorpi, H., Niittymäki, I., Biström, M., Raitila, A., Saharinen, J., Nousiainen, K., Lehtonen, H.J., Heliövaara, E., Puhakka, J., Tuupanen, S., Sousa, S., Seruca, R., Ferreira, A.M., Hofstra, R.M.W., Mecklin, J.-P., Järvinen, H., Ristimäki, A., Ørntoft, T.F., Hautaniemi, S., Arango, D., Karhu, A., Aaltonen, L.A.: Candidate driver genes in microsatellite-unstable colorectal cancer. International Journal of Cancer **130**(7), 1558–1566 (2012)
54. Wang, R.-J., Wu, P., Cai, G.-X., Wang, Z.-M., Xu, Y., Peng, J.-J., Sheng, W.-Q., Lu, H.-F., Cai, S.-J.: Down-regulated myh11 expression correlates with poor prognosis in stage ii and iii colorectal cancer. Asian Pacific Journal of Cancer Prevention **15**(17), 7223–7228 (2014)
55. Ganesh, S., Sier, C.F.M., Griffioen, G., Vloedgraven, H.J.M., de Boer, A., Welvaart, K., Van de Velde, C.J.H., Van Krieken, J.H.J.M., Verheijen, J.H., Lamers, C.B.H.W., Verspaget, H.W.: Prognostic relevance of plasminogen activators and their inhibitors in colorectal cancer. Cancer Research **54**(15), 4065–4071 (1994)
56. Baker, E.A., Leaper, D.J.: The plasminogen activator and matrix metalloproteinase systems in colorectal cancer: relationship to tumour pathology. European Journal of Cancer **39**(7), 981–988 (2003)
57. Wojtukiewicz, M.Z., Zacharski, L.R., Memoli, V.A., Kisiel, W., Kudryk, B.J., Rousseau, S.M., Stump, D.C.: Indirect activation of blood coagulation in colon cancer. Thrombosis and Haemostasis **62**(4), 1062–1066 (1989)
58. Wong, H.P.S., Yu, L., Lam, E.K.Y., Tai, E.K.K., Wu, W.K.K., Cho, C.-H.: Nicotine promotes colon tumor growth and angiogenesis through β-adrenergic activation. Toxicological Sciences **97**(2), 279–287 (2007)
59. Liu, X., Wu, W.K.K., Yu, L., Li, Z.J., Sung, J.J.Y., Zhang, S.T., Cho, C.H.: Epidermal growth factor-induced esophageal cancer cell proliferation requires transactivation of β-adrenoceptors. Journal of Pharmacology and Experimental Therapeutics **326**(1), 69–75 (2008)
60. Andersson, A., Edén, P., Lindgren, D., Nilsson, J., Lassen, C., Heldrup, J., Fontes, M., Borg, Å., Mitelman, F., Johansson, B., Höglund, M., Fioretos, T.: Gene expression profiling of leukemic cell lines reveals conserved molecular signatures among subtypes with specific genetic aberrations. Leukemia **19**(6), 1042–1050 (2005)
61. Ning, B., Liu, G., Liu, Y., Su, X., Anderson, G.J., Zheng, X., Chang, Y., Guo, M., Liu, Y., Zhao, Y., Nie, G.: 5-aza-2'-deoxycytidine activates iron uptake and heme biosynthesis by increasing c-myc nuclear localization and binding to the e-boxes of transferrin receptor 1 (tfr1) and ferrochelatase (fech) genes. Journal of Biological Chemistry **286**(43), 37196–37206 (2011)
62. Krishnamurthy, P., Ross, D.D., Nakanishi, T., Bailey-Dell, K., Zhou, S., Mercer, K.E., Sarkadi, B., Sorrentino, B.P., Schuetz, J.D.: The stem cell marker bcrp/abcg2 enhances hypoxic cell survival through interactions with heme. Journal of Biological Chemistry **279**(23), 24218–24225 (2004)
63. Stegmaier, K., Ross, K.N., Colavito, S.A., O'Malley, S., Stockwell, B.R., Golub, T.R.: Gene expression–based high-throughput screening (ge-hts) and application to leukemia differentiation. Nature Genetics **36**(3), 257–263 (2004)
64. Gery, S., Gombart, A.F., Yi, W.S., Koeffler, C., Hofmann, W.-K., Koeffler, H.P.: Transcription profiling of c/ebp targets identifies per2 as a gene implicated in myeloid leukemia. Blood **106**(8), 2827–2836 (2005)
65. Sun, Y., Boyd, K., Xu, W., Ma, J., Jackson, C.W., Fu, A., Shillingford, J.M., Robinson, G.W., Hennighausen, L., Hitzler, J.K., Ma, Z., Morris, S.W.: Acute myeloid leukemia-associated mkl1 (mrtf-a) is a key regulator of mammary gland function. Molecular and Cellular Biology **26**(15), 5809–5826 (2006)
66. Broxmeyer, H.E., Williams, D.E., Lu, L., Cooper, S., Anderson, S.L., Beyer, G.S., Hoffman, R., Rubin, B.Y.: The suppressive influences of human tumor necrosis factors on bone marrow hematopoietic progenitor cells from normal donors and patients with leukemia: synergism of tumor necrosis factor and interferon-gamma. The Journal of Immunology **136**(12), 4487–4495 (1986)
67. Berthon, C., Driss, V., Liu, J., Kuranda, K., Leleu, X., Jouy, N., Hetuin, D., Quesnel, B.: In acute myeloid leukemia, b7-h1 (pd-l1) protection of blasts from cytotoxic t cells is induced by tlr ligands and interferon-gamma and can be reversed using mek inhibitors. Cancer Immunology, Immunotherapy **59**(12), 1839–1849 (2010)
68. Gombart, A.F., Krug, U., O'Kelly, J., An, E., Vegesna, V., Koeffler, H.P.: Aberrant expression of neutrophil and macrophage-related genes in a murine model for human neutrophil-specific granule deficiency. Journal of Leukocyte Biology **78**(5), 1153–1165 (2005)
69. Scotland, S., Saland, E., Skuli, N., De Toni, F., Boutzen, H., Micklow, E., Senegas, I., Peyraud, R., Peyriga, L., Theodoro, F., Dumon, E., Martineau, Y., Danet-Desnoyers, G., Bono, F., Rocher, C., Levade, C., Manenti, S., Junot, C., Portais, J.C., Alet, N., Récher, C., Selak, M.A., Carroll, M., Sarry, J.E.: Mitochondrial energetic and akt status mediate metabolic effects and apoptosis of metformin in human leukemic cells. Leukemia **27**(11), 2129–2138 (2013)
70. Chen, Y., Xu, Q., Ji, D., Wei, Y., Chen, H., Li, T., Wan, B., Yuan, L., Huang, R., Chen, G.: Inhibition of pentose phosphate pathway suppresses acute myelogenous leukemia. Tumor Biology **37**(5), 6027–6034 (2016)
71. Ferrer, A., Ollila, J., Tobin, G., Nagy, B., Thunberg, U., Aalto, Y., Vihinen, M., Vilpo, J., Rosenquist, R., Knuutila, S.: Different gene expression in immunoglobulin-mutated and immunoglobulin-unmutated forms of chronic lymphocytic leukemia. Cancer Genetics and Cytogenetics **153**(1), 69–72 (2004)

72. Niini, T., Vettenranta, K., Hollmén, J., Larramendy, M.L., Aalto, Y., Wikman, H., Nagy, B., Seppänen, J.K., Salvador, A.F., Mannila, H., Saarinen-Pihkala, U.M., Knuutila, S.: Expression of myeloid-specific genes in childhood acute lymphoblastic leukemia-a cdna array study. Leukemia **16**(11), 2213–2221 (2002)
73. Wittkowski, H., Frosch, M., Wulffraat, N., Goldbach-Mansky, R., Kallinich, T., Kuemmerle-Deschner, J., Frühwald, M.C., Dassmann, S., Pham, T.-H., Roth, J., Foell, D.: S100a12 is a novel molecular marker differentiating systemic-onset juvenile idiopathic arthritis from other causes of fever of unknown origin. Arthritis & Rheumatology **58**(12), 3924–3931 (2008)
74. Huang, G., Shigesada, K., Ito, K., Wee, H.-J., Yokomizo, T., Ito, Y.: Dimerization with pebp2β protects runx1/aml1 from ubiquitin–proteasome-mediated degradation. The EMBO Journal **20**(4), 723–733 (2001)
75. Cortes, J., Thomas, D., Koller, C., Giles, F., Estey, E., Faderl, S., Garcia-Manero, G., McConkey, D., Patel, G., Guerciolini, R., Wright, J., Kantarjian, H.: Phase i study of bortezomib in refractory or relapsed acute leukemias. Clinical Cancer Research **10**(10), 3371–3376 (2004)
76. Pigazzi, M., Ricotti, E., Germano, G., Faggian, D., Aricò, M., Basso, G.: camp response element binding protein (creb) overexpression creb has been described as critical for leukemia progression. Haematologica **92**(10), 1435–1437 (2007)
77. Gombart, A.F., O'Kelly, J., Saito, T., Koeffler, H.P.: Regulation of the CAMP gene by 1,25(oh)2d3 in various tissues. The Journal of Steroid Biochemistry and Molecular Biology **103**(3), 552–557 (2007)
78. Nakamura, T., Datta, R., Sherman, M.L., Kufe, D.: Regulation of c-jun gene expression by camp in hl-60 myeloid leukemia cells. Journal of Biological Chemistry **265**(35), 22011–22015 (1990)
79. Yang, W.-C., Lin, P.-M., Yang, M.-Y., Liu, Y.-C., Chang, C.-S., Chou, W.-C., Hsu, J.-F., Huang, C.-T., Cho, S.-F., Yu, W.-H., Lin, S.-F.: Higher lipocalin 2 expression may represent an independent favorable prognostic factor in cytogenetically normal acute myeloid leukemia. Leukemia & Lymphoma **54**(8), 1614–1625 (2013)
80. Lobry, C., Ntziachristos, P., Ndiaye-Lobry, D., Oh, P., Cimmino, L., Zhu, N., Araldi, E., Hu, W., Freund, J., Abdel-Wahab, O., Ibrahim, S., Skokos, D., Armstrong, S.A., Levine, R.L., Park, C.Y., Aifantis, I.: Notch pathway activation targets aml-initiating cell homeostasis and differentiation. Journal of Experimental Medicine **210**(2), 301–319 (2013)
81. Quere, R., Andradottir, S., Brun, A.C.M., Zubarev, R.A., Karlsson, G., Olsson, K., Magnusson, M., Cammenga, J., Karlsson, S.: High levels of the adhesion molecule cd44 on leukemic cells generate acute myeloid leukemia relapse after withdrawal of the initial transforming event. Leukemia **25**(3), 515–526 (2011)
82. Mahadevan, D., DiMento, J., Croce, K.D., Riley, C., George, B., Fuchs, D., Mathews, T., Wilson, C., Lobell, M.: Transcriptosome and serum cytokine profiling of an atypical case of myelodysplastic syndrome with progression to acute myelogenous leukemia. American Journal of Hematology **81**(10), 779–786 (2006)
83. Menssen, H.D., Renkl, H.J., Rodeck, U., Maurer, J., Notter, M., Schwartz, S., Reinhardt, R., Thiel, E.: Presence of wilms' tumor gene (wt1) transcripts and the wt1 nuclear protein in the majority of human acute leukemias. Leukemia **9**(6), 1060–1067 (1995)
84. Sui, S., Wang, X., Zheng, H., Guo, H., Chen, T., Ji, D.-M.: Gene set enrichment and topological analyses based on interaction networks in pediatric acute lymphoblastic leukemia. Oncology Letters **10**(6), 3354–3362 (2015)
85. Masetti, R., Bertuccio, S.N., Astolfi, A., Chiarini, F., Lonetti, A., Indio, V., De Luca, M., Bandini, J., Serravalle, S., Franzoni, M., Pigazzi, M., Martelli, A.M., Basso, G., Locatelli, F., Pession, A.: Hh/gli antagonist in acute myeloid leukemia with cbfa2t3-glis2 fusion gene. Journal of Hematology & Oncology **10**(1), 26 (2017)
86. Klimenkova, O., Klimiankou, M., Schmidt, A., Stocking, C., Zeidler, C., Link, D.C., Welte, K., Skokowa, J.: Co-acquisition of runx1 and csf3r mutations transforms hematopoietic progenitor cells of cn patients into more primitive highly proliferative blasts: evidence in cn patients and in a mouse model. Blood **124**(21), 223–223 (2014)
87. Trojani, A., Lodola, M., Di Camillo, B., Rossi, G., Capucci, A., Perego, A., Pogliani, E.M., Orlandi, E., Iurlo, A., Malato, S., Corradini, P., Cairoli, R., Bregni, M., Artale, S., Morra, E., Pungolino, E.: Gene expression profiling of cd34+/lin- cells of patients with chronic myeloid leukemia at diagnosis and after 12 months of nilotinib. Blood **124**(21), 5177–5177 (2014)
88. Charfi, C., Voisin, V., Levros, L.-C., Edouard, E., Rassart, E.: Gene profiling of graffi murine leukemia virus induced lymphoid leukemias: identification of leukemia markers and fmn2 as a potential oncogene. Blood **18**(8), 165–179 (2010)
89. Irish, J.M., Kotecha, N., Nolan, G.P.: Mapping normal and cancer cell signalling networks: towards single-cell proteomics. Nature Reviews Cancer **6**(2), 146–155 (2006)
90. Olsnes, A.M., Motorin, D., Ryningen, A., Zaritskey, A.Y., Bruserud, Ø.: T lymphocyte chemotactic chemokines in acute myelogenous leukemia (aml): local release by native human aml blasts and systemic levels of cxcl10 (ip-10), ccl5 (rantes) and ccl17 (tarc). Cancer Immunology, Immunotherapy **55**(7), 830–840 (2006)
91. Kornblau, S.M., McCue, D., Singh, N., Chen, W., Estrov, Z., Coombes, K.R.: Recurrent expression signatures of cytokines and chemokines are present and are independently prognostic in acute myelogenous leukemia and myelodysplasia. Blood **116**(20), 4251–4261 (2010)
92. Peeters, M., Poon, A.: Down syndrome and leukemia: unusual clinical aspects and unexpected methotrexate sensitivity. European Journal of Pediatrics **146**(4), 416–422 (1987)
93. Becher, H., Weber, M., Löhr, G.W.: Purine nucleotide synthesis in normal and leukemic blood cells. Klinische Wochenschrift **56**(6), 275–283 (1978)
94. Le Dieu, R., Taussig, D.C., Ramsay, A.G., Mitter, R., Miraki-Moud, F., Fatah, R., Lee, A.M., Lister, T.A., Gribben, J.G.: Peripheral blood t cells in acute myeloid leukemia (aml) patients at diagnosis have abnormal phenotype and genotype and form defective immune synapses with aml blasts. Blood **114**(18), 3909–3916 (2009)
95. Dunussi-Joannopoulos, K., Weinstein, H.J., Nickerson, P.W., Strom, T.B., Burakoff, S.J., Croop, J.M., Arceci, R.J.: Irradiated b7-1 transduced primary acute myelogenous leukemia (aml) cells can be used as therapeutic vaccines in murine aml. Blood **87**(7), 2938–2946 (1996)
96. Appelbaum, F.R.: Haematopoietic cell transplantation as immunotherapy. Nature **411**(6835), 385–389 (2001)

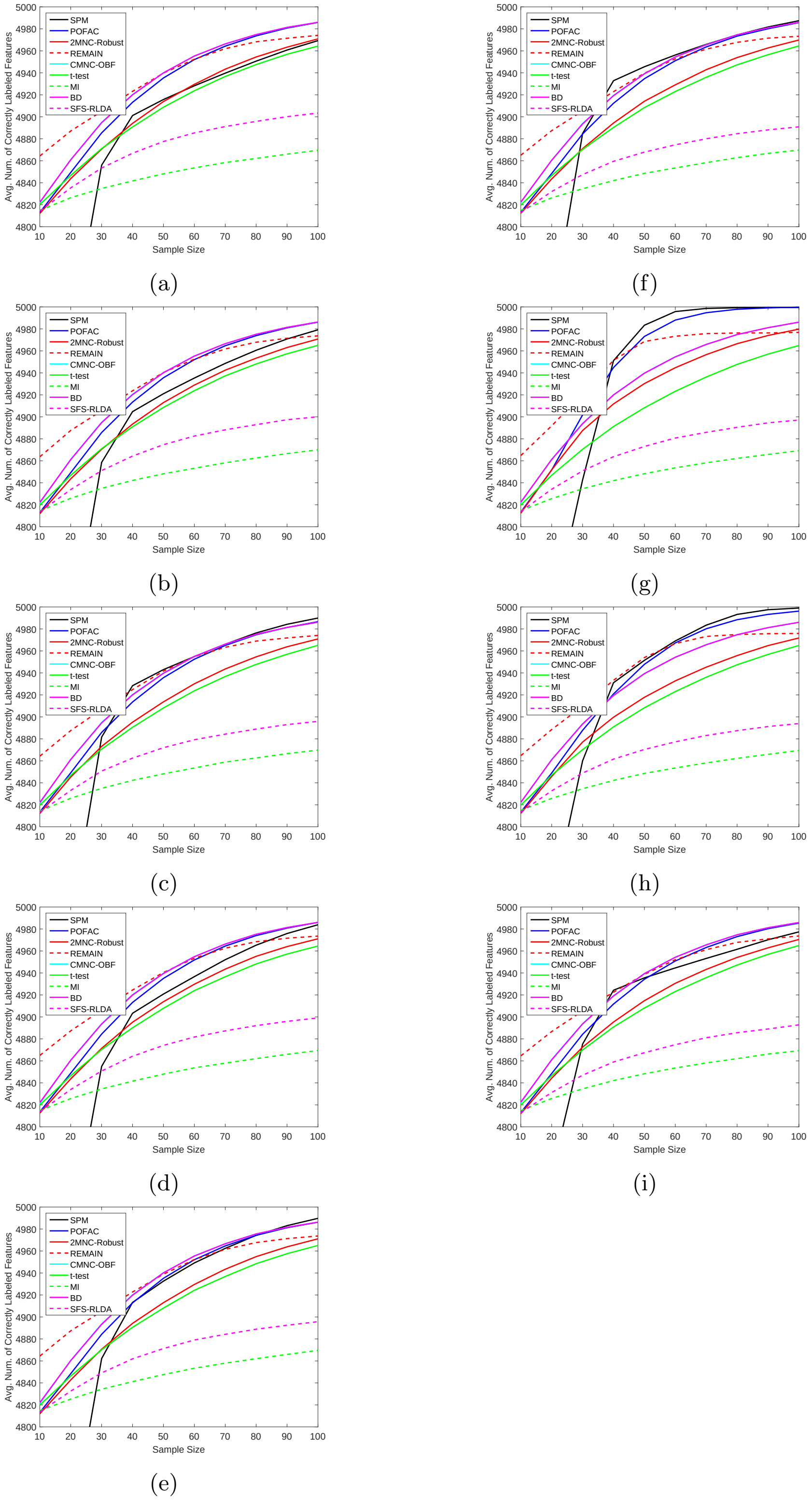

Figure 1: Average number of correctly labeled features versus sample size for redundant markers when block size is 5, and ρ_0 and ρ_1 are (a) $0.1, 0.1$, (b) $0.1, 0.5$, (c) $0.1, 0.9$, (d) $0.5, 0.1$, (e) $0.5, 0.5$, (f) $0.5, 0.9$, (g) $0.9, 0.1$, (h) $0.9, 0.5$, and (i) $0.9, 0.9$, respectively.

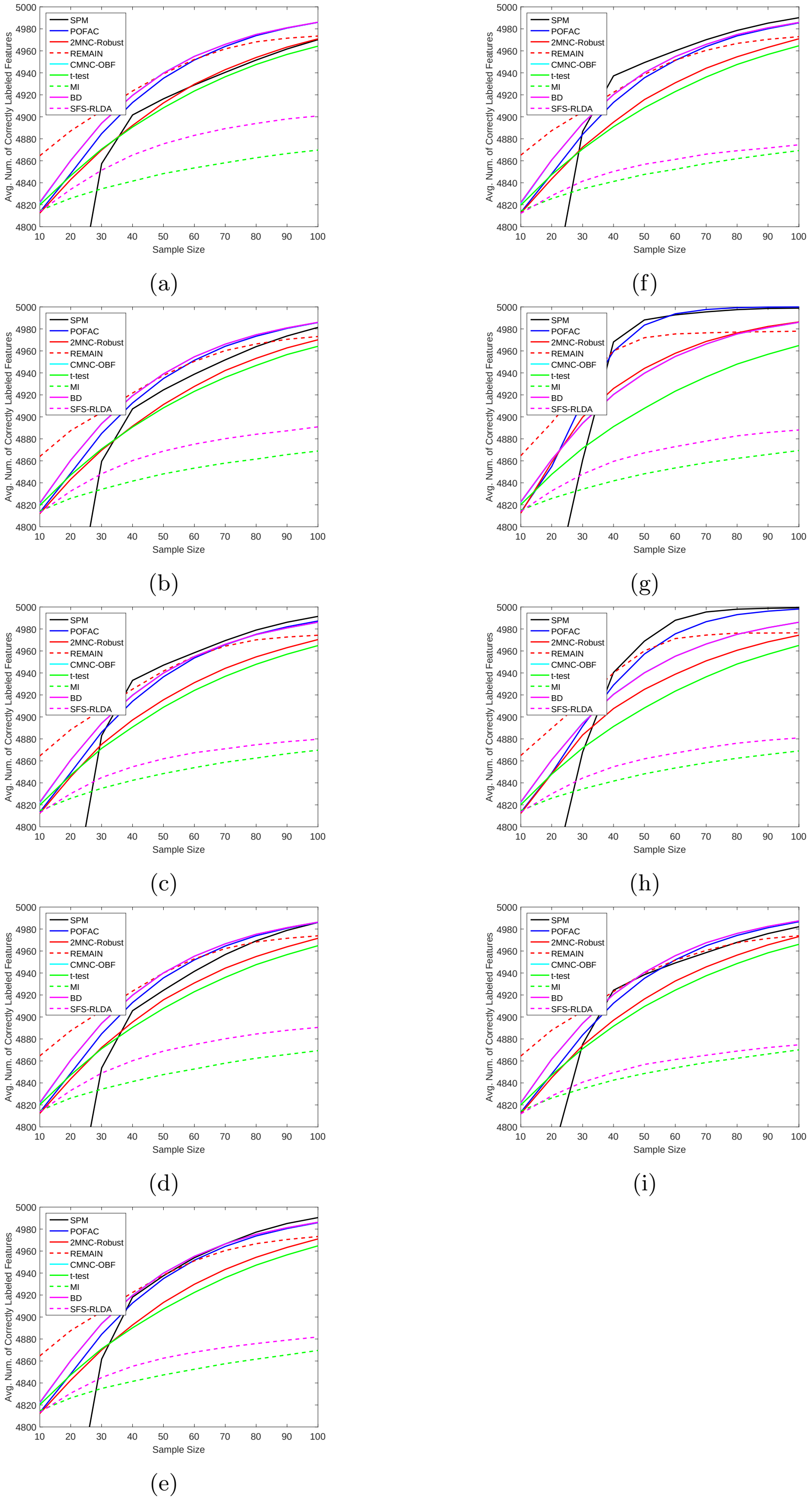

Figure 2: Average number of correctly labeled features versus sample size for redundant markers when block size is 10, and ρ_0 and ρ_1 are (a) $0.1, 0.1$, (b) $0.1, 0.5$, (c) $0.1, 0.9$, (d) $0.5, 0.1$, (e) $0.5, 0.5$, (f) $0.5, 0.9$, (g) $0.9, 0.1$, (h) $0.9, 0.5$, and (i) $0.9, 0.9$, respectively.

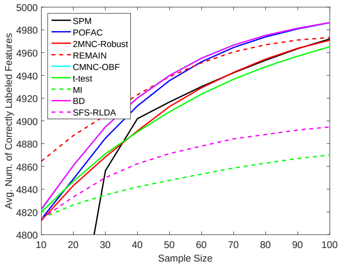

(a)

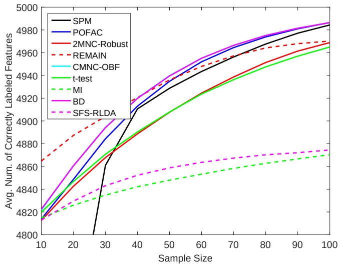

(b)

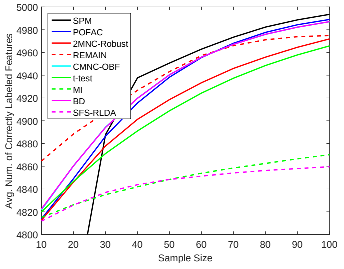

(c)

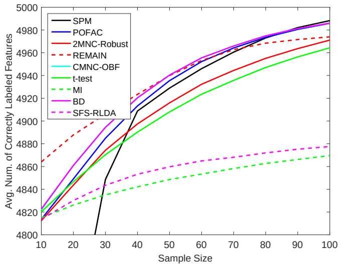

(d)

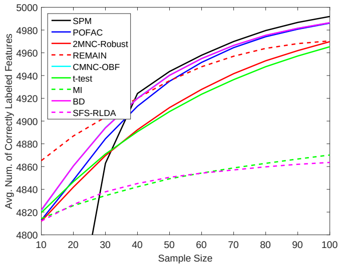

(e)

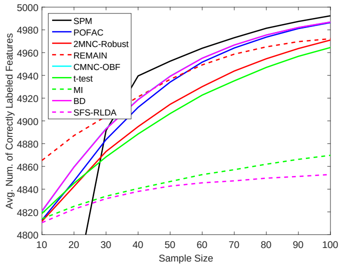

(f)

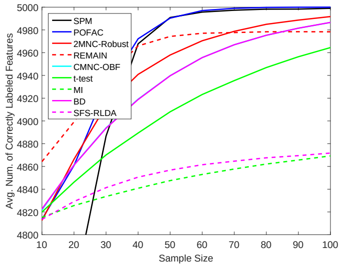

(g)

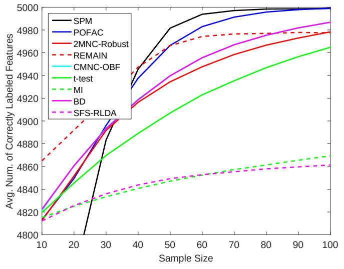

(h)

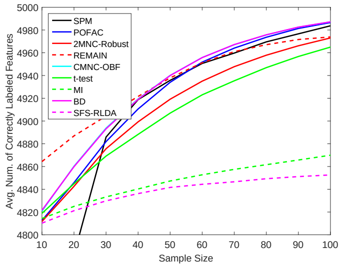

(i)

Figure 3: Average number of correctly labeled features versus sample size for redundant markers when block size is 20, and ρ_0 and ρ_1 are (a) $0.1, 0.1$, (b) $0.1, 0.5$, (c) $0.1, 0.9$, (d) $0.5, 0.1$, (e) $0.5, 0.5$, (f) $0.5, 0.9$, (g) $0.9, 0.1$, (h) $0.9, 0.5$, and (i) $0.9, 0.9$, respectively.

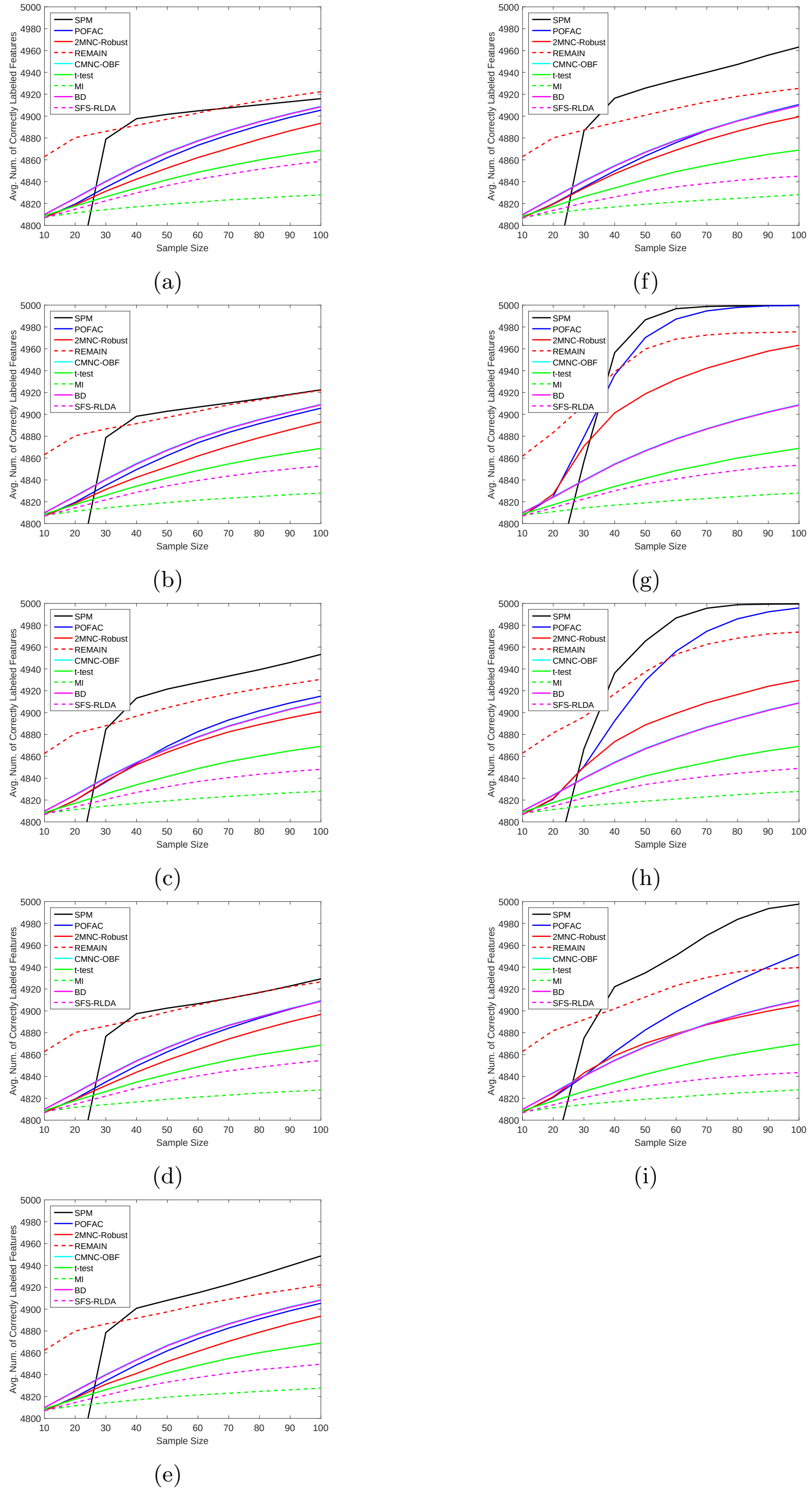

Figure 4: Average number of correctly labeled features versus sample size for synergetic markers when block size is 5, and ρ_0 and ρ_1 are (a) $0.1, 0.1$, (b) $0.1, 0.5$, (c) $0.1, 0.9$, (d) $0.5, 0.1$, (e) $0.5, 0.5$, (f) $0.5, 0.9$, (g) $0.9, 0.1$, (h) $0.9, 0.5$, and (i) $0.9, 0.9$, respectively.

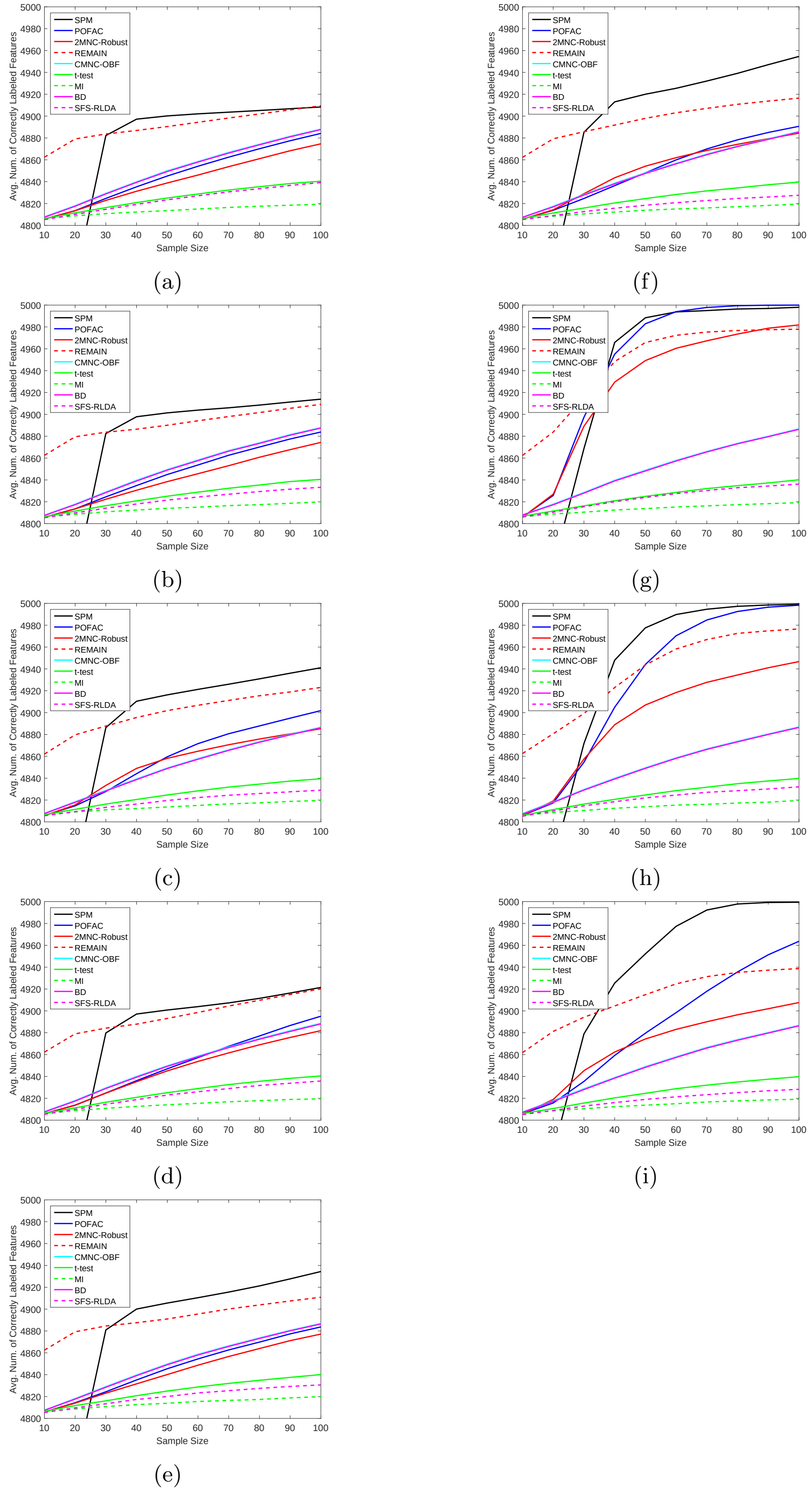

Figure 5: Average number of correctly labeled features versus sample size for synergetic markers when block size is 10, and ρ_0 and ρ_1 are (a) $0.1, 0.1$, (b) $0.1, 0.5$, (c) $0.1, 0.9$, (d) $0.5, 0.1$, (e) $0.5, 0.5$, (f) $0.5, 0.9$, (g) $0.9, 0.1$, (h) $0.9, 0.5$, and (i) $0.9, 0.9$, respectively.

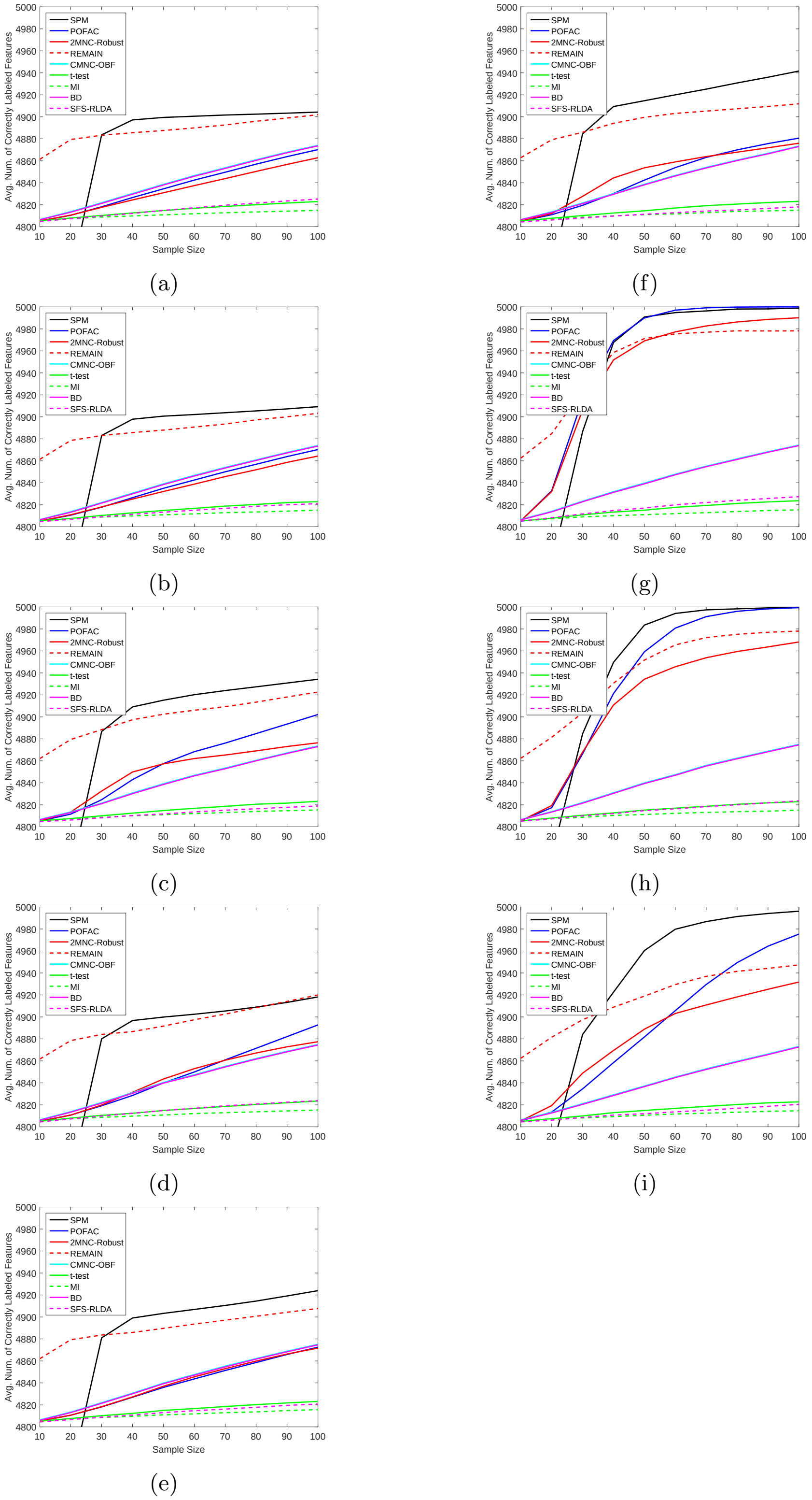

Figure 6: Average number of correctly labeled features versus sample size for synergetic markers when block size is 20, and ρ_0 and ρ_1 are (a) $0.1, 0.1$, (b) $0.1, 0.5$, (c) $0.1, 0.9$, (d) $0.5, 0.1$, (e) $0.5, 0.5$, (f) $0.5, 0.9$, (g) $0.9, 0.1$, (h) $0.9, 0.5$, and (i) $0.9, 0.9$, respectively.

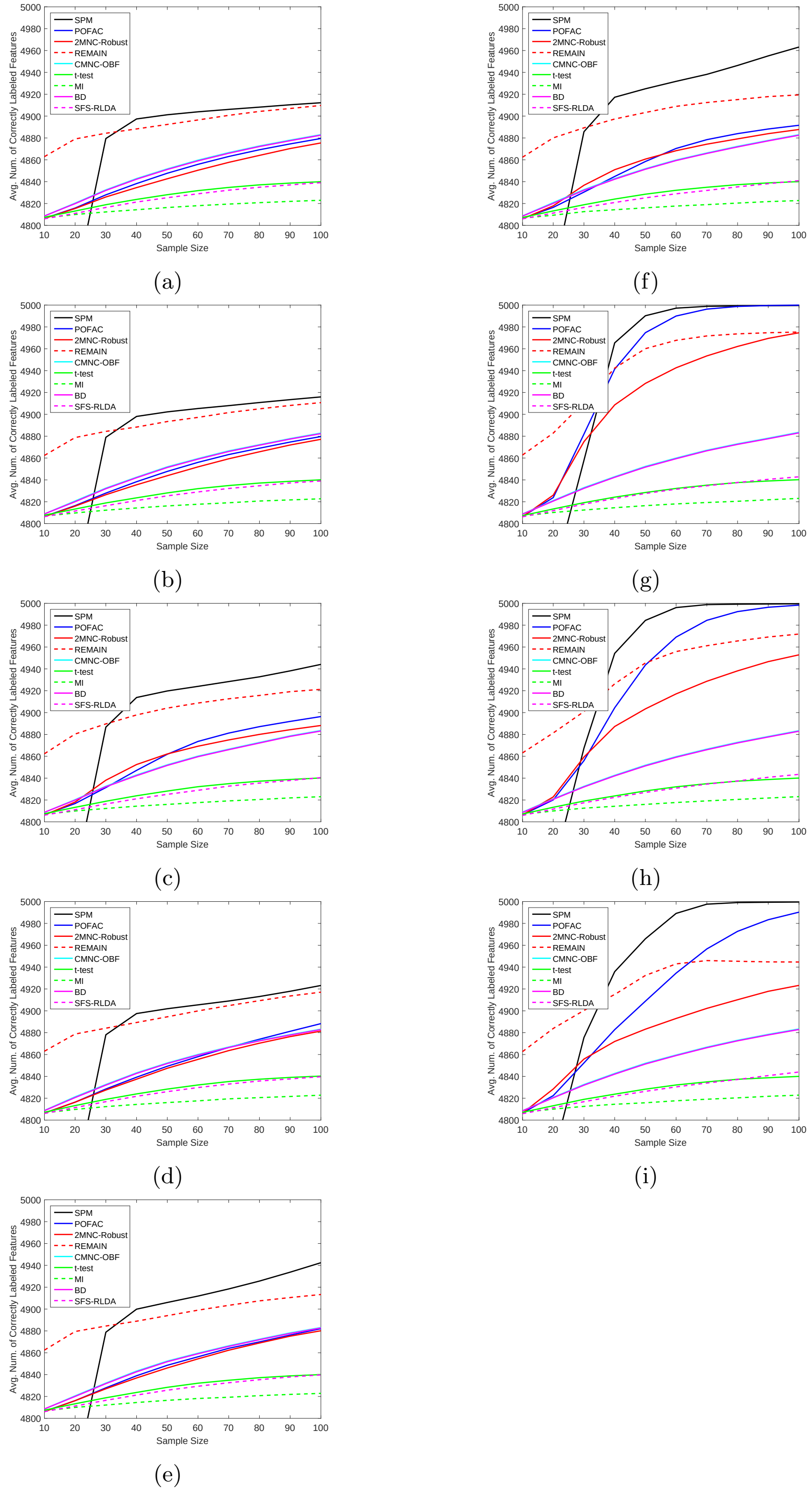

Figure 7: Average number of correctly labeled features versus sample size for marginal markers when block size is 5, and ρ_0 and ρ_1 are (a) $0.1, 0.1$, (b) $0.1, 0.5$, (c) $0.1, 0.9$, (d) $0.5, 0.1$, (e) $0.5, 0.5$, (f) $0.5, 0.9$, (g) $0.9, 0.1$, (h) $0.9, 0.5$, and (i) $0.9, 0.9$, respectively.

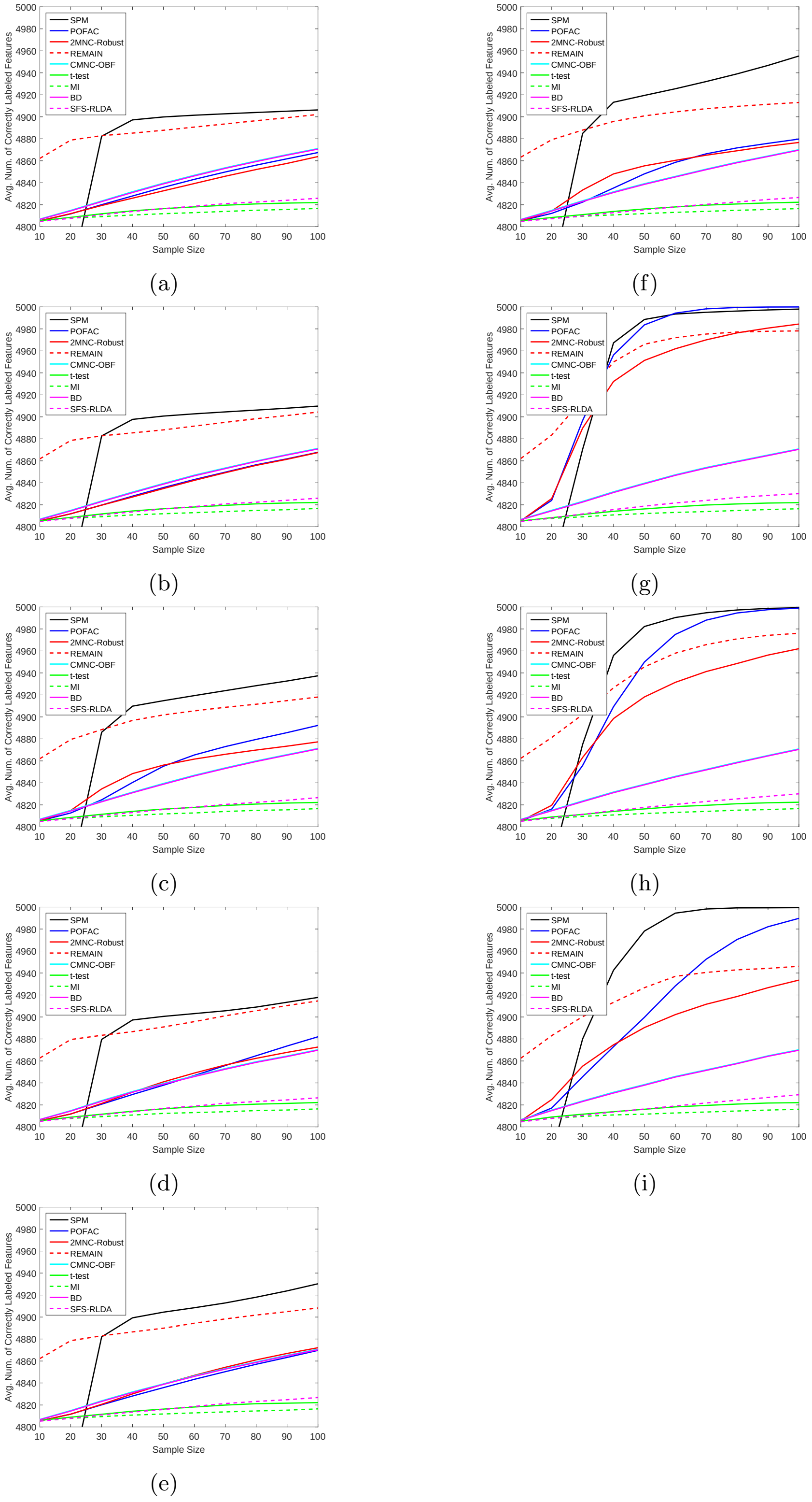

Figure 8: Average number of correctly labeled features versus sample size for marginal markers when block size is 10, and ρ_0 and ρ_1 are (a) $0.1, 0.1$, (b) $0.1, 0.5$, (c) $0.1, 0.9$, (d) $0.5, 0.1$, (e) $0.5, 0.5$, (f) $0.5, 0.9$, (g) $0.9, 0.1$, (h) $0.9, 0.5$, and (i) $0.9, 0.9$, respectively.

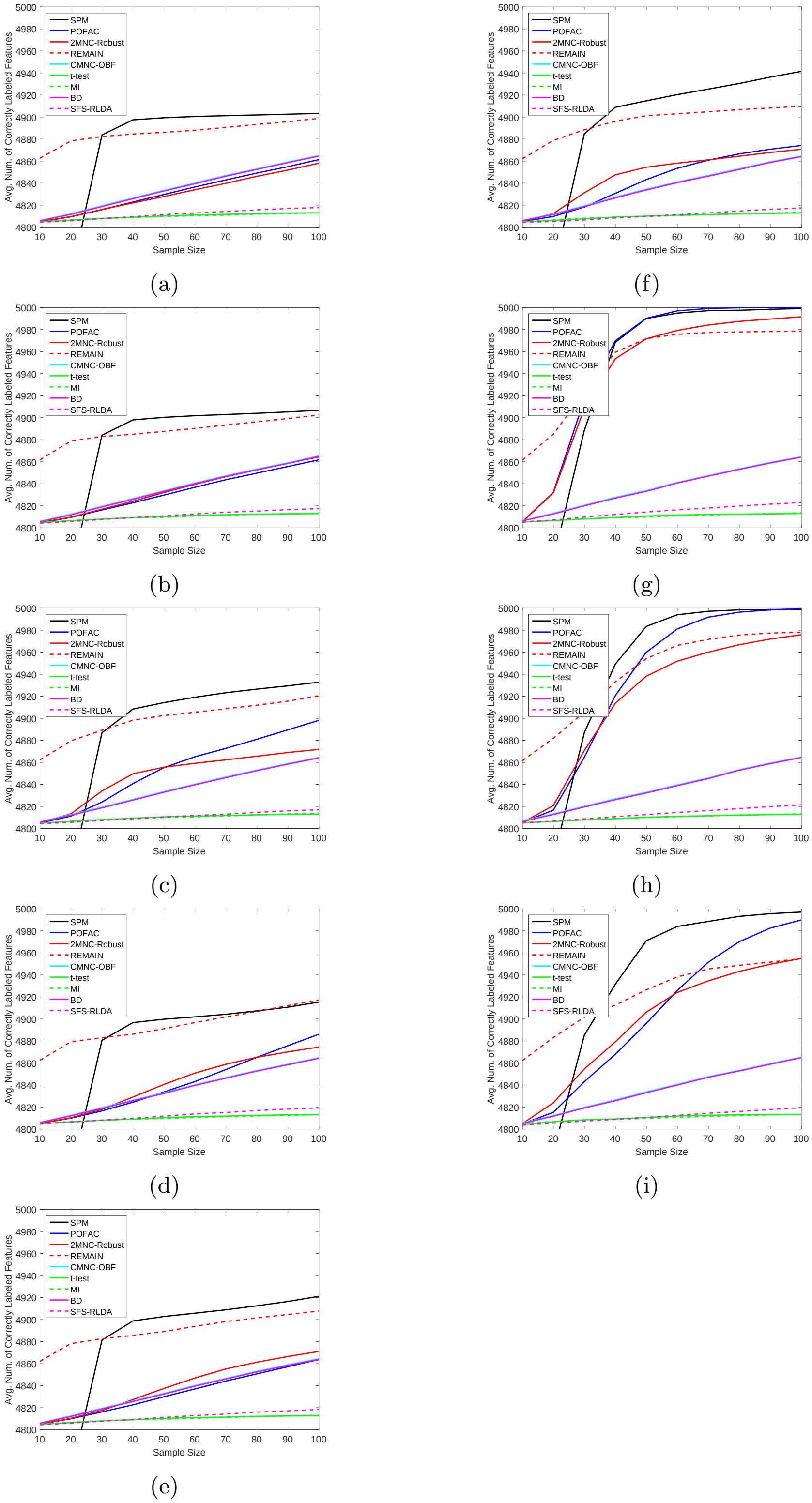

Figure 9: Average number of correctly labeled features versus sample size for marginal markers when block size is 20, and ρ_0 and ρ_1 are (a) $0.1, 0.1$, (b) $0.1, 0.5$, (c) $0.1, 0.9$, (d) $0.5, 0.1$, (e) $0.5, 0.5$, (f) $0.5, 0.9$, (g) $0.9, 0.1$, (h) $0.9, 0.5$, and (i) $0.9, 0.9$, respectively.

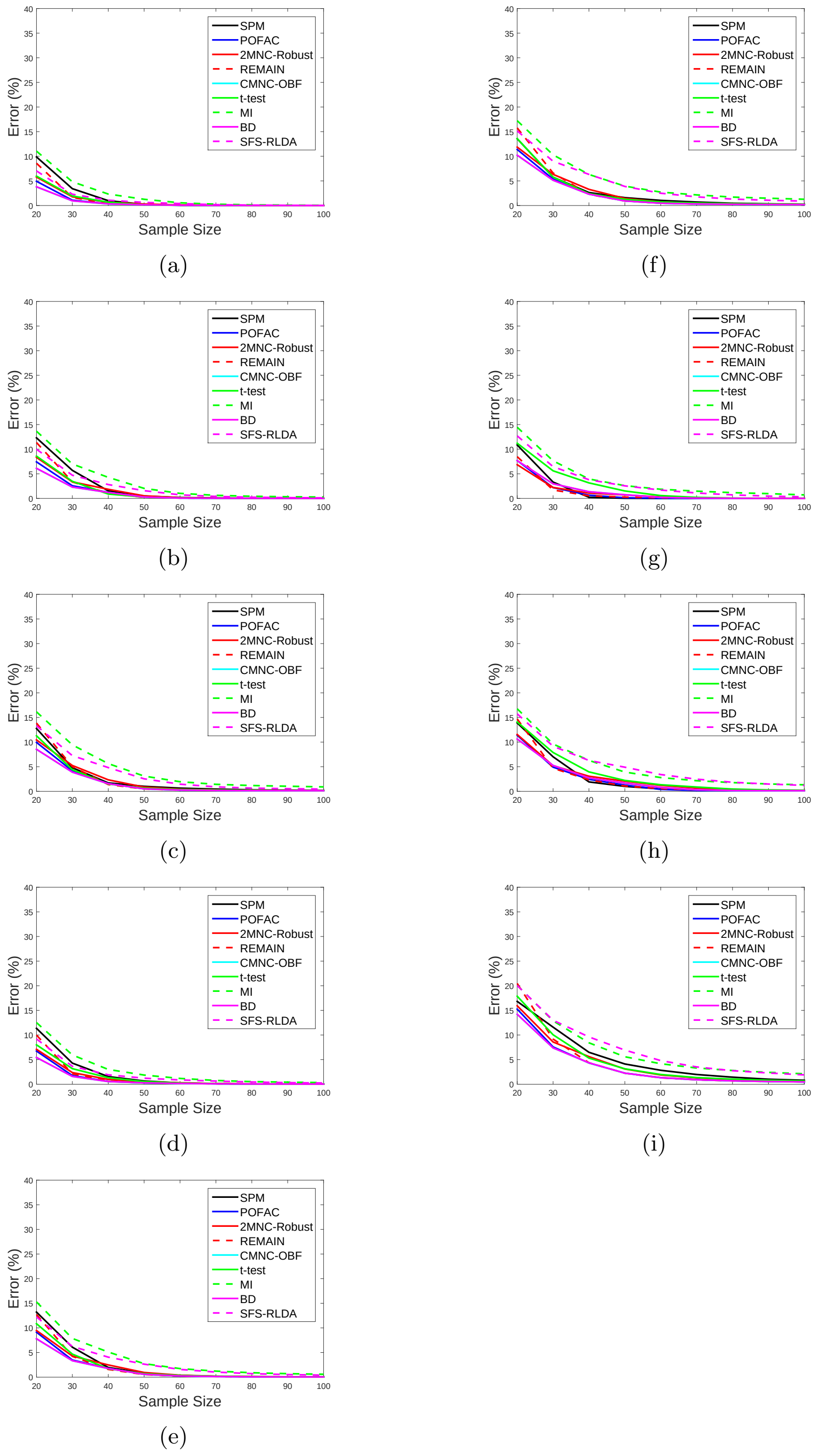

Figure 10: Misclassification error of feature selection algorithms versus sample size for redundant markers when block size is 5, and ρ_0 and ρ_1 are (a) $0.1, 0.1$, (b) $0.1, 0.5$, (c) $0.1, 0.9$, (d) $0.5, 0.1$, (e) $0.5, 0.5$, (f) $0.5, 0.9$, (g) $0.9, 0.1$, (h) $0.9, 0.5$, and (i) $0.9, 0.9$, respectively.

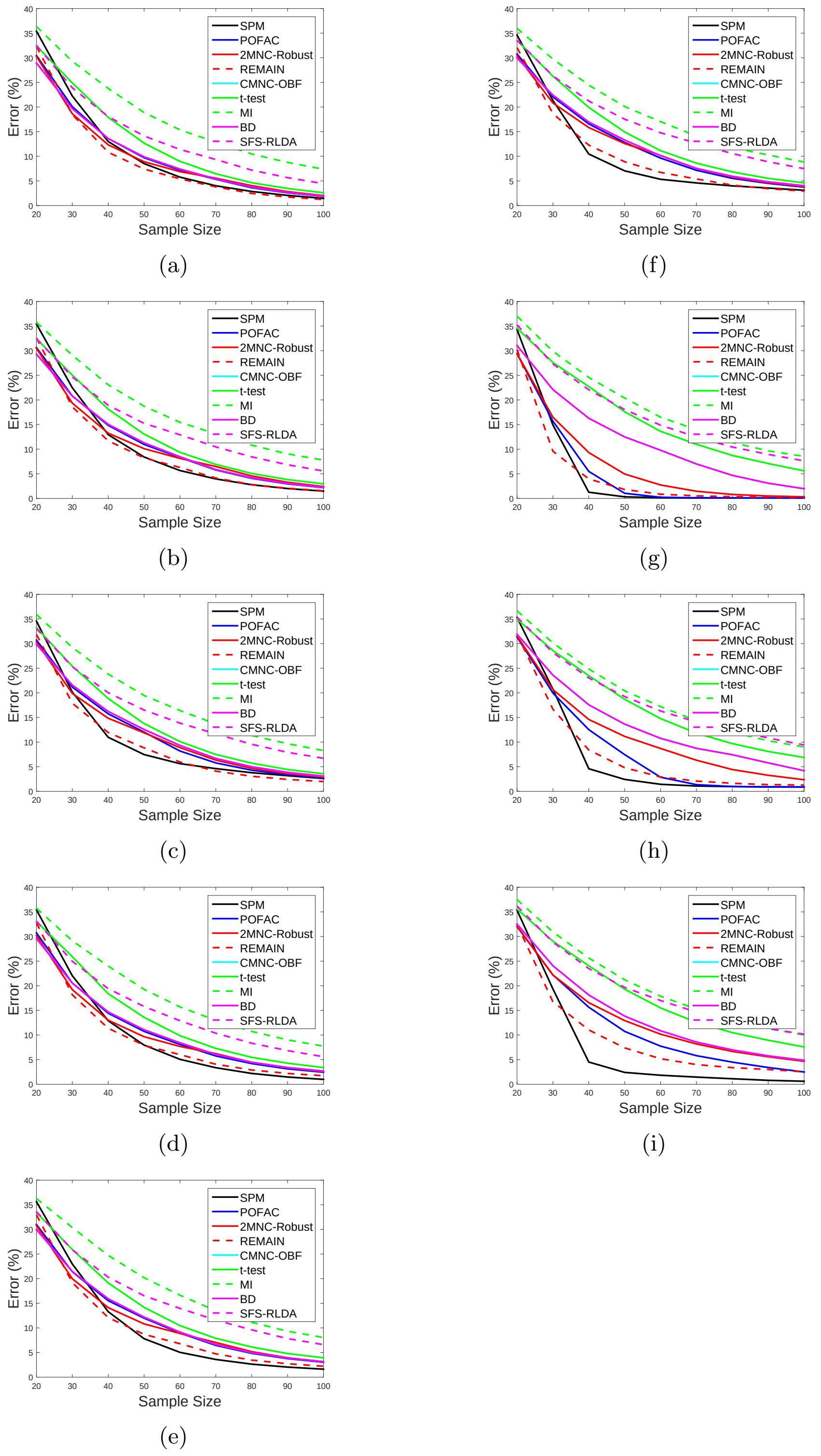

Figure 11: Misclassification error of feature selection algorithms versus sample size for synergetic markers when block size is 5, and ρ_0 and ρ_1 are (a) $0.1, 0.1$, (b) $0.1, 0.5$, (c) $0.1, 0.9$, (d) $0.5, 0.1$, (e) $0.5, 0.5$, (f) $0.5, 0.9$, (g) $0.9, 0.1$, (h) $0.9, 0.5$, and (i) $0.9, 0.9$, respectively.

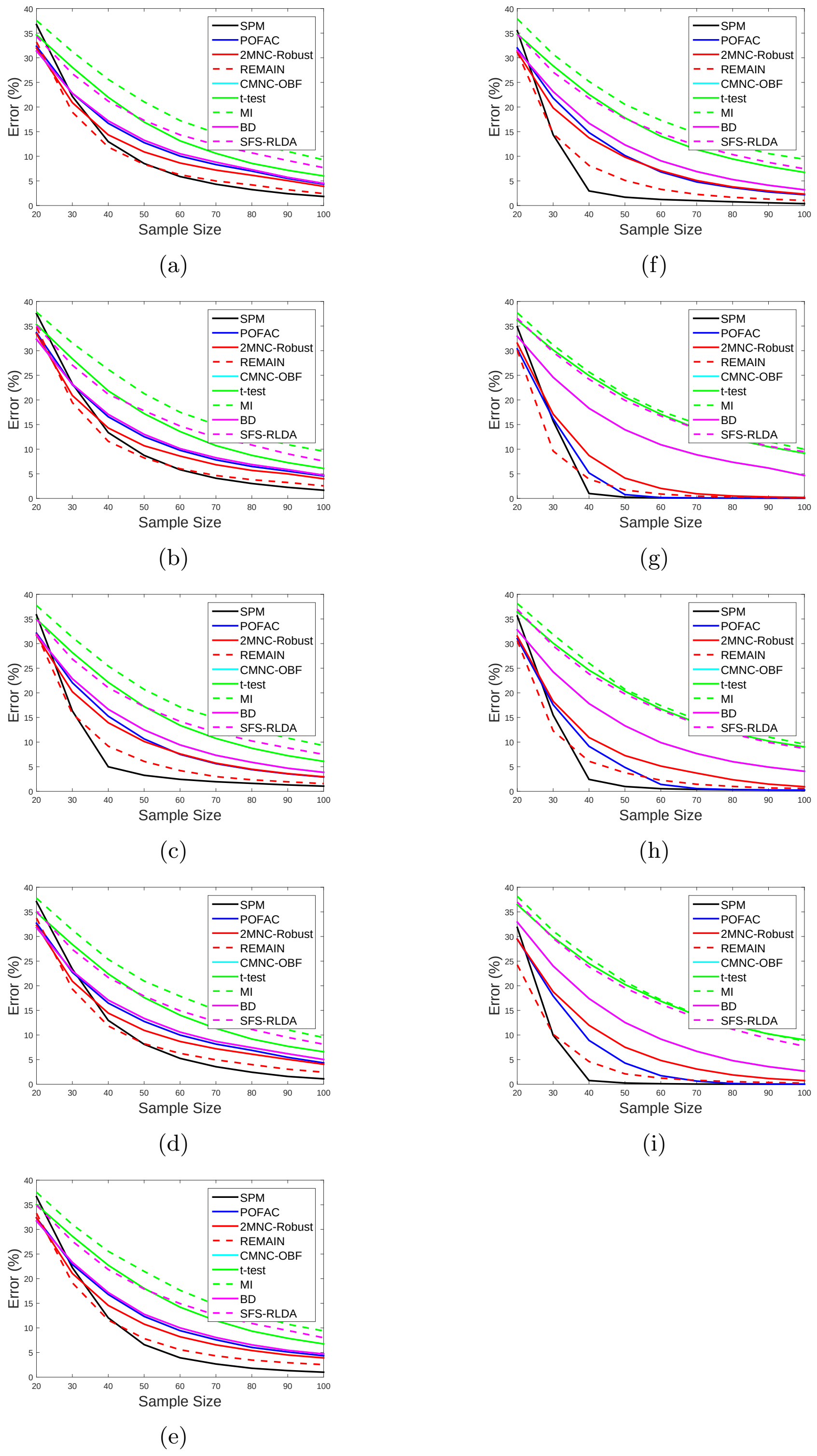

Figure 12: Misclassification error of feature selection algorithms versus sample size for marginal markers when block size is 5, and ρ_0 and ρ_1 are (a) $0.1, 0.1$, (b) $0.1, 0.5$, (c) $0.1, 0.9$, (d) $0.5, 0.1$, (e) $0.5, 0.5$, (f) $0.5, 0.9$, (g) $0.9, 0.1$, (h) $0.9, 0.5$, and (i) $0.9, 0.9$, respectively.

Table 1: Top Genes of Breast Cancer Using REMAIN

Rank	Gene	Rank	Gene	Rank	Gene	Rank	Gene
1	DCT	26	KIF20A	51	CENPF	76	POLQ
2	ZNF192	27	ABCA11P	52	NR2E3	77	HHIPL2
3	ZP2	28	KIF18A	53	ATIC	78	IL1F9
4	PCSK6	29	NARS	54	SMARCA4	79	RBM26
5	CEACAM7	30	PSMB7	55	HSPA4	80	AUP1
6	MPHOSPH10	31	CCT7	56	DDX18	81	TRIM48
7	MUC5AC	32	H2AFZ	57	CDC16	82	CHRNA9
8	PHTF1	33	PSMC2	58	HOXB13	83	MTM1
9	CCT8	34	ETF1	59	ART3	84	ESPL1
10	TMEM189-UBE2V1 UBE2V1	35	PWP1	60	LST1	85	C19orf22
11	SON	36	UBXN1	61	PAPPA2	86	MAPRE1
12	GSS	37	C7orf28B CCZ1	62	MARS	87	STMN1
13	PTBP1	38	NID1	63	HOMER1	88	EIF2S1
14	NAGA	39	TUBB3	64	MET	89	TOP2A
15	DGUOK	40	MTHFD1	65	ACTL8	90	MGST3
16	KIAA0649	41	TIAL1	66	MYO16	91	SRM
17	PEX10	42	KIAA0101	67	ASCC2	92	SAMM50
18	HMMR	43	FADD	68	TBCB	93	DNMT1
19	LONP1	44	NPC1	69	MLLT10	94	TUBG1
20	HDGFRP3	45	GOLIM4	70	C16orf80	95	CHMP1A
21	ABCB1	46	LSM7	71	UBL5	96	GNL2
22	HTR2C	47	MLLT3	72	APPL1	97	NASP
23	BICD2	48	ZNF167	73	NOL8	98	PSMB4
24	BRAP	49	APCS	74	EXOSC4	99	RAB13
25	ZNF227	50	AP3D1	75	MED31	100	STXBP1

Table 2: Top Genes of Breast Cancer Using CMNC-OBF

Rank	Gene	Rank	Gene	Rank	Gene	Rank	Gene
1	DCT	26	LST1	51	MYL10	76	BICD2
2	PHTF1	27	ACTL8	52	SREK1IP1	77	ATF7IP
3	ZNF227	28	HOMER1	53	C16orf80	78	ST3GAL5
4	ZP2	29	CELF1	54	KIAA0101	79	TOP2A
5	CEACAM7	30	CENPF	55	UBE2W	80	POLQ
6	MUC5AC	31	TRIM48	56	HHIPL2	81	DKFZp686O1327
7	PCSK6	32	UBXN1	57	METTL3	82	MYCBP
8	ABCA11P	33	CHRNA9	58	CRIM1	83	BDH1
9	KIAA0649	34	MLANA	59	NPC1	84	BIRC5
10	ZNF192	35	ESPL1	60	TMEM8B	85	PRC1
11	MET	36	SNCA	61	PAN2	86	MLH3
12	BRAP	37	ART3	62	RSF1	87	NUDT21
13	HDGFRP3	38	COL9A1	63	SPINT2	88	H2AFZ
14	KIF18A	39	MKI67	64	SHMT1	89	ZXDB
15	HMMR	40	TESK1	65	LILRA5	90	SMARCA2
16	SRP72	41	HK3	66	PPME1	91	TRPM8
17	ASCC2	42	CEP192	67	ZNF167	92	F5
18	APPL1	43	ASPM	68	NAGA	93	EDNRB
19	SON	44	HOXB13	69	ATF7	94	GPR56
20	KIF20A	45	FADD	70	PDGFD	95	HMGA2
21	NUP98	46	SNX1	71	HSPA4	96	BUB1B
22	MTM1	47	THSD1 THSD1P1	72	FANCA	97	OXTR
23	MYO16	48	NR2E3	73	CDK12	98	DHRS7B
24	SILV	49	FANCI	74	AARS	99	SPTLC2
25	GOLIM4	50	MLLT3	75	N4BP2L2	100	KIF23

Table 3: Top Genes of Breast Cancer Using POFAC

Rank	Gene	Rank	Gene	Rank	Gene	Rank	Gene
1	DCT	26	NPC1	51	PAFAH1B1	76	NOL8
2	PHTF1	27	SRP72	52	ZNF167	77	ALG8
3	MUC5AC	28	SNCA	53	CEP135	78	DAZ1,2,3,4
4	HUWE1	29	BRAP	54	SHMT1	79	SPRR1A
5	MLANA	30	APPL1	55	DNAH6	80	ZEB1
6	ZNF227	31	FGF1	56	VWF	81	NR2E3
7	ZP2	32	CELF1	57	SYNRG	82	CRIM1
8	ACTN2	33	FAT4	58	C18orf1	83	FADD
9	SPINT2	34	KIF18A	59	MYCBP	84	TMEM189-UBE2V1
10	KIAA0649	35	MYO16	60	GOLIM4	85	VAMP4
11	PCSK6	36	HMMR	61	ASPM	86	NOD1
12	CEACAM7	37	CEP192	62	ATF7IP	87	CHRNA9
13	TPT1	38	ASCC2	63	FXYD1	88	STXBP1
14	HDGFRP3	39	SON	64	BAAT	89	PSD4
15	ZNF192	40	KDM6A	65	ACTL8	90	NID1
16	SNX1	41	UBXN1	66	COL9A1	91	RHOQ
17	ABCA11P	42	CDC16	67	NAGA	92	SPG7
18	CSRP3	43	ANGPT1	68	SMARCA2	93	HEY1
19	LST1	44	EDNRB	69	NEB	94	DHRS7B
20	GAS6	45	MTM1	70	CHST4	95	ZFP161
21	ZFHX4	46	METTL3	71	MED31	96	PPME1
22	MET	47	KIF20A	72	HOMER1	97	CA12
23	SILV	48	TRIM48	73	ART3	98	HEY2
24	NFIB	49	HOXB13	74	MPHOSPH10	99	N4BP2L2
25	NUP98	50	THSD1 THSD1P1	75	CENPF	100	SPTLC2

Table 4: Top Genes of Breast Cancer Using limma

Rank	Gene	Rank	Gene	Rank	Gene	Rank	Gene
1	CENPF	26	CRIM1	51	TOP2A	76	EZH1
2	KIAA0101	27	PRC1	52	HJURP	77	DRAP1
3	PDGFD	28	HOXA5	53	KCND3	78	PKMYT1
4	H2AFZ	29	TUBB3	54	GRK5	79	EXOSC4
5	FANCA	30	CENPA	55	CCT5	80	ADRA2A
6	ASPM	31	MELK	56	MCM10	81	FLRT2
7	GPR56	32	PBK	57	RACGAP1	82	KIFC1
8	FOXM1	33	CCNB1	58	CD302	83	CARHSP1
9	BUB1B	34	GINS2	59	CDKN3	84	SMARCA4
10	CENPE	35	RRM2	60	SPARCL1	85	SYNC
11	KIF18A	36	CEP68	61	KIF4A	86	MAB21L1
12	MLF1IP	37	BUB1	62	UBE2S	87	CDC20
13	FANCI	38	NEK2	63	IGF1	88	TRIP13
14	SF1	39	TPX2	64	SQLE	89	LDB2
15	MKI67	40	METTL13	65	LEPR	90	TDO2
16	GPRASP1	41	SNRPB	66	DDX39	91	DTL
17	MMRN2	42	ANKRD12	67	TXNIP	92	TMEM208
18	KIF23	43	GPC3	68	IL1RN	93	C14orf139
19	ESPL1	44	CDK1	69	ITM2A	94	MTCH2
20	NUSAP1	45	AIMP2	70	CCNB2	95	TALDO1
21	EXO1	46	KIF20A	71	SPAG5	96	TEAD4
22	DLGAP5	47	PTN	72	STMN1	97	C1orf106
23	COG8 PDF	48	FAM129A	73	KIF14	98	PGP
24	HMMR	49	C16orf80	74	FAM172A	99	TUBB2C
25	BIRC5	50	CX3CR1	75	SETBP1	100	N4BP2L1

Table 5: Top Breast Cancer Pathways Using CMNC-OBF Gene Set

Rank	Pathway	P.W. size	#Found	P-value
1	Gonadotropin-releasing hormone receptor P.W.	235	44	$4.93E-05$
2	p53 P.W.	88	22	$8.34E-05$
3	Ubiquitin proteasome P.W.	66	18	$1.26E-04$
4	CCKR sig. map	173	34	$1.40E-04$
5	Huntington disease	139	29	$1.60E-04$
6	FAS sig. P.W.	34	12	$1.71E-04$
7	De novo purine biosyn.	30	10	$8.86E-04$
8	Heterotrimeric G-prot. sig. P.W.-Gi alpha & Gs alpha med. P.W.	164	29	$2.00E-03$
9	PDGF sig. P.W.	149	27	$2.01E-03$
10	p53 P.W. feedback loops 2	53	13	$2.57E-03$
11	5HT2 type receptor med. sig. P.W.	67	15	$2.96E-03$
12	TCA cycle	10	5	$3.24E-03$
13	Oxytocin receptor med. sig. P.W.	58	13	$5.37E-03$
14	B cell activation	72	15	$5.63E-03$
15	5HT4 type receptor med. sig. P.W.	33	9	$5.77E-03$
16	Beta2 adrenergic receptor sig. P.W.	46	11	$6.32E-03$
17	Beta3 adrenergic receptor sig. P.W.	28	8	$6.90E-03$
18	Muscarinic acetylcholine receptor 1 and 3 sig. P.W.	60	13	$7.02E-03$
19	Vitamin B6 metabolism	4	3	$7.34E-03$
20	ATP synthesis	8	4	$8.24E-03$

Table 6: Top Breast Cancer Pathways Using REMAIN Gene Set

Rank	Pathway	P.W. size	#Found	P-value
1	Ubiquitin proteasome P.W.	66	19	$3.33E-07$
2	Angiogenesis	176	26	$3.40E-04$
3	FAS sig. P.W.	34	9	$7.23E-04$
4	TCA cycle	10	5	$7.31E-04$
5	Heme biosyn.	13	5	$2.29E-03$
6	CCKR sig. map	173	23	$2.72E-03$
7	p53 P.W.	88	14	$3.91E-03$
8	Huntington disease	139	19	$4.55E-03$
9	Gonadotropin-releasing hormone receptor P.W.	235	28	$4.61E-03$
10	Cell cycle	22	6	$4.67E-03$
11	De novo purine biosyn.	30	7	$5.38E-03$
12	p53 P.W. feedback loops 2	53	9	$1.26E-02$
13	VEGF sig. P.W.	72	11	$1.29E-02$
14	De novo pyrimidine deoxyribonucleotide biosyn.	13	4	$1.32E-02$
15	ATP synthesis	8	3	$1.86E-02$
16	Parkinson disease	100	13	$2.40E-02$
17	p38 MAPK P.W.	42	7	$2.84E-02$
18	Hypoxia response via HIF activation	33	6	$2.86E-02$
19	Cytoskeletal regulation by Rho GTPase	83	11	$3.19E-02$
20	PI3 kinase P.W.	55	8	$3.95E-02$

Table 7: Top Breast Cancer Pathways Using POFAC Gene Set

Rank	Pathway	P.W. size	#Found	P-value
1	CCKR sig. map	173	36	$3.82E-05$
2	p53 P.W.	88	21	$2.61E-04$
3	Ubiquitin proteasome P.W.	66	17	$4.18E-04$
4	TCA cycle	10	6	$5.46E-04$
5	De novo purine biosyn.	30	10	$9.73E-04$
6	Gonadotropin-releasing hormone receptor P.W.	235	39	$1.52E-03$
7	Heme biosyn.	13	6	$2.06E-03$
8	FAS sig. P.W.	34	10	$2.41E-03$
9	Cytoskeletal regulation by Rho GTPase	83	17	$4.48E-03$
10	Huntington disease	139	24	$7.05E-03$
11	p53 P.W. feedback loops 2	53	12	$7.43E-03$
12	Vitamin B6 metabolism	4	3	$7.59E-03$
13	PDGF sig. P.W.	149	25	$8.47E-03$
14	De novo pyrimidine deoxyribonucleotide biosyn.	13	5	$1.00E-02$
15	Angiogenesis	176	28	$1.08E-02$
16	Dopamine receptor med. sig. P.W.	59	12	$1.60E-02$
17	5HT4 type receptor med. sig. P.W.	33	8	$1.82E-02$
18	Beta3 adrenergic receptor sig. P.W.	28	7	$2.26E-02$
19	PI3 kinase P.W.	55	11	$2.28E-02$
20	Cell cycle	22	6	$2.33E-02$

Table 8: Top Breast Cancer Pathways Using SPM Gene Set

Rank	Pathway	P.W. size	#Found	P-value
1	Ubiquitin proteasome P.W.	66	16	$6.25E-07$
2	Integrin signalling P.W.	192	28	$1.72E-06$
3	Pyrimidine Metabolism	11	6	$2.87E-05$
4	Parkinson disease	100	15	$3.09E-04$
5	Alzheimer disease-presenilin P.W.	124	16	$9.76E-04$
6	Angiogenesis	176	19	$2.63E-03$
7	Cytoskeletal regulation by Rho GTPase	83	11	$4.69E-03$
8	Cell cycle	22	5	$6.22E-03$
9	ATP synthesis	8	3	$8.67E-03$
10	S-adenosylmethionine biosyn.	3	2	$1.09E-02$
11	Huntington disease	139	14	$1.54E-02$
12	Salvage pyrimidine deoxyribonucleotides	4	2	$1.87E-02$
13	Inflammation med. by chemokine and cytokine sig. P.W.	261	22	$1.98E-02$
14	Notch sig. P.W.	44	6	$2.86E-02$
15	Insulin/IGF P.W.-mitogen act. prot. kinase/MAP kinase cas.	33	5	$3.00E-02$
16	De novo pyrimidine deoxyribonucleotide biosyn.	13	3	$3.08E-02$
17	Biotin biosyn.	1	1	$5.04E-02$
18	Apoptosis sig. P.W.	122	11	$5.63E-02$
19	Formyltetrahydroformate biosyn.	8	2	$6.53E-02$
20	T cell activation	96	9	$6.55E-02$

Table 9: Top Breast Cancer Pathways Using Top 2000 limma Gene Set

Rank	Pathway	P.W. size	#Found	P-value
1	Gonadotropin-releasing hormone receptor P.W.	235	49	$1.45E-06$
2	Ubiquitin proteasome P.W.	66	21	$4.34E-06$
3	Parkinson disease	100	26	$1.19E-05$
4	Huntington disease	139	31	$3.44E-05$
5	CCKR sig. map	173	36	$3.50E-05$
6	FAS sig. P.W.	34	13	$4.59E-05$
7	Cell cycle	22	10	$8.48E-05$
8	DNA replication	35	12	$2.38E-04$
9	p53 P.W.	88	21	$2.46E-04$
10	De novo purine biosynthesis	30	10	$9.42E-04$
11	Cholesterol biosynthesis	13	6	$2.01E-03$
12	Cytoskeletal regulation by Rho GTPase	83	17	$4.30E-03$
13	Apoptosis sig. P.W.	122	22	$5.71E-03$
14	Angiotensin II-stimulated sig. through G proteins and beta-arrestin	39	10	$5.99E-03$
15	Glycolysis	22	7	$6.70E-03$
16	JAK/STAT sig. P.W.	17	6	$7.27E-03$
17	Inflammation mediated by chemokine and cytokine sig. P.W.	261	39	$7.74E-03$
18	ATP synthesis	8	4	$8.47E-03$
19	PI3 kinase P.W.	55	12	$9.43E-03$
20	De novo pyrimidine deoxyribonucleotide biosynthesis	13	5	$9.83E-03$

Table 10: Top Breast Cancer Pathways Using Significant limma Gene Set

Rank	Pathway	P.W. size	#Found	P-value
1	Huntington disease	139	16	$1.16E-04$
2	Ubiquitin proteasome P.W.	66	10	$2.70E-04$
3	Cytoskeletal regulation by Rho GTPase	83	11	$4.18E-04$
4	p53 P.W.	88	11	$6.73E-04$
5	Cell cycle	22	5	$1.68E-03$
6	Formyltetrahydroformate biosynthesis	8	3	$3.70E-03$
7	JAK/STAT sig. P.W.	17	4	$4.29E-03$
8	CCKR sig. map	173	14	$7.38E-03$
9	Integrin sig. P.W.	192	15	$7.70E-03$
10	FAS sig. P.W.	34	5	$1.02E-02$
11	Vitamin B6 metabolism	4	2	$1.04E-02$
12	Cholesterol biosynthesis	13	3	$1.38E-02$
13	Serine glycine biosynthesis	5	2	$1.58E-02$
14	Tetrahydrofolate biosynthesis	5	2	$1.58E-02$
15	Asparagine and aspartate biosynthesis	5	2	$1.58E-02$
16	p53 P.W. feedback loops 2	53	6	$1.68E-02$
17	Angiotensin II-stimulated sig. through G proteins and beta-arrestin	39	5	$1.75E-02$
18	Gonadotropin-releasing hormone receptor P.W.	235	16	$1.97E-02$
19	Insulin/IGF P.W.-protein kinase B sig. cascade	41	5	$2.11E-02$
20	Alzheimer disease-presenilin P.W.	124	10	$2.19E-02$

Table 11: Top Genes of Colon Cancer Using REMAIN

Rank	Gene	Rank	Gene	Rank	Gene	Rank	Gene
1	EPHA7	26	PPARGC1A	51	LATS1	76	LOC100505801
2	NBLA00301	27	MYLK	52	PMPCB	77	ATP6
3	LOC100133920 LOC286297	28	MAGEA4	53	ATP1A2	78	DEFB125
4	PDK4	29	IL1F6	54	PCDH11X PCDH11Y	79	CNTNAP5
5	MYH11	30	C4orf17	55	C8orf71	80	YEATS2
6	SCN7A	31	OCR1	56	TCEAL2	81	LOC100130275
7	CPNE4	32	RNF17	57	ZNF440	82	MCTS1
8	KCNIP4	33	LONRF2	58	APBB2	83	LOC729121
9	LOC100507186	34	RTDR1	59	C22orf36	84	C21orf41
10	RYR3	35	LOC100287728 ZNF75D	60	PTPN1	85	LOC645188
11	GAGE1,12,4,5,6,7	36	LOC100507741	61	HMP19	86	VWA3B
12	CCDC7	37	ONECUT2	62	LOC51145	87	LOC283089
13	RSPO2	38	LOC285943	63	PURG	88	PWRN1
14	LOC641515	39	NCRNA00052	64	HOXC8	89	D21S2090E
15	S100A7	40	C12orf40	65	GJD2	90	LOC93444
16	GAGE1,12,2,4,5,6,7,8	41	CYP19A1	66	TMPRSS13	91	C10orf90
17	TRIM10	42	UBE2DNL	67	LOC400236	92	LOC100130264
18	CACNA1C	43	RASGRF1	68	HSPB8	93	SRRM5
19	C19orf26	44	UBE2W	69	ERBB4	94	LOC149086
20	LOC652276	45	C14orf34	70	DEPDC6	95	ATP8B5P
21	TSGA13	46	POLR3B	71	ARHGEF12	96	LOC653110
22	LOC100508567	47	LOC100133308	72	ASB5	97	AGTR2
23	LOC400620	48	NIPAL1	73	LOC646522	98	HTR7
24	LOC219688	49	CDH4	74	RNF219	99	HIST1H1T
25	OPN5	50	MAGIX	75	LOC100144602	100	GPM6A

Table 12: Top Genes of Colon Cancer Using CMNC-OBF

Rank	Gene	Rank	Gene	Rank	Gene	Rank	Gene
1	CPNE4	26	CPS1	51	CACNA1C	76	C6orf58
2	GAGE1,12,4,5,6,7	27	CECR4	52	NCRNA00230A NCRNA00230B	77	CNR1
3	GAGE1,12,2,4,5,6,7,8	28	NOL4	53	CARHSP1	78	SERPINC1
4	S100A7	29	CD36	54	CHRNA9	79	FLJ13744
5	EPHA7	30	FUT9	55	MYH11	80	LYVE1
6	MAGEA4	31	IL17A	56	TSPAN2	81	LOC100271840
7	LOC100133920 LOC286297	32	HTR2C	57	CIB4	82	PEG3
8	GPM6A	33	NBLA00301	58	PCDHA1,10,11,12,13,2,3,4,5,6,7,8,9 PCDHAC1,2	83	APOA4
9	SLC2A2	34	TTR	59	DKK1	84	LGALS4
10	CEACAM5	35	F5	60	AGTR1	85	IGHD
11	ADIPOQ	36	MEOX2	61	MAGEA5	86	CDH1
12	CT45A1,2,3,4,5,6	37	CELF3	62	MAGEA12	87	HP HPR
13	HORMAD1	38	PCDHB6	63	DIO1	88	LCA5
14	FGG	39	FSTL5	64	CBLN4	89	FCRL5
15	AHSG	40	C8orf71	65	PDE1A	90	C20orf103
16	MMP8	41	EPHA8	66	MUC16	91	MARCO
17	TF	42	ALB	67	C6orf15	92	LOC100506377
18	LOC100507186	43	APOH	68	BEAN1	93	SNAP25
19	SLC14A1	44	CD24	69	IL26	94	C17orf104
20	FLJ37786	45	TFPI	70	CTAG1A CTAG1B	95	DES FAM48A
21	TTN	46	CLC	71	SLC10A2	96	TCEAL2
22	FGA	47	CDH17	72	GPR64	97	TMC5
23	ZIC1	48	PLIN1	73	MUC5AC	98	NRK
24	GAGE3	49	ASB4	74	SERPINB13	99	PLBD1
25	ASB5	50	MAGEC2	75	SYNM	100	HPR

Table 13: Top Genes of Colon Cancer Using POFAC

Rank	Gene	Rank	Gene	Rank	Gene	Rank	Gene
1	EPHA7	26	FOXP2	51	ANK2	76	CST11
2	CPNE4	27	GNGT1	52	FLJ32955	77	GNRHR2
3	LOC100133920 LOC286297	28	RIBC1	53	ZNF396	78	GABRR3
4	SCN7A	29	C3orf43	54	ASB5	79	GPR68
5	NBLA00301	30	FLJ42627	55	C6orf168	80	LOC100507164
6	LOC100507186	31	LOC57399	56	MAP3K2	81	EYA3
7	RSPO2	32	PARD3B	57	ST8SIA1	82	ATN1
8	MYH11	33	PTGS1	58	IMPG1	83	BCL8
9	KCNIP4	34	BRD7P3	59	HSPB8	84	PLCB2
10	PDK4	35	NRXN1	60	SLC35F4	85	LOC728543
11	ATP1A2	36	ATXN1	61	LOC100129250	86	CCDC148
12	GPM6A	37	NCRNA00247	62	TES	87	GRM5
13	TCEAL2	38	LHX9	63	LMOD1	88	LOC100130548
14	RYR3	39	A2LD1	64	DNAJC6	89	NANOS2
15	HAND1	40	NFATC2	65	AFF3	90	FLJ34521
16	CACNA1C	41	SLC35A2	66	PRM1	91	C3orf67
17	RIMKLB	42	CXorf31	67	LOC100129476	92	ALDOAP2
18	MYLK	43	CSNK1E LOC400927	68	KCNIP2	93	KCNC2
19	SEMG2	44	LIPI	69	SSBP2	94	S100A7
20	LONRF2	45	LOC286190	70	PDLIM5	95	FLJ32224
21	DPP6	46	C21orf67	71	BIVM	96	PHLDB1
22	GRIK3	47	SYCE1	72	GAGE1,12,2,4,5,6,7,8	97	ELAVL4
23	SYNPO2	48	LOC342918	73	USP49	98	LOC100509020
24	GAGE1,12,4,5,6,7	49	LOC100129129	74	AKAP9	99	BLOC1S3
25	POLD3	50	HCG27	75	ANGPTL3	100	CAMK2B

Table 14: Top Genes of Colon Cancer Using limma

Rank	Gene	Rank	Gene	Rank	Gene	Rank	Gene
1	TSPAN2	26	ATP10B	51	WASF1	76	TNFSF13
2	PLBD1	27	TMEM22	52	PEG3	77	LEPR
3	TMC5	28	PRELP	53	CD109	78	SPATS2
4	CD36	29	PPP1R3C	54	PRNP	79	FAM65A
5	GPM6A	30	PLN	55	PPIL4 ZC3H12D	80	EPM2AIP1
6	MAB21L2	31	ASPN	56	NEXN	81	CEACAM5
7	AKAP12	32	SYNM	57	SGCD	82	CFL2
8	SPP1	33	SCG2	58	MLLT11	83	TMEM47
9	NPR3	34	BMP7	59	TMEM65	84	NRK
10	AGTR1	35	GULP1	60	JAZF1	85	CLEC11A
11	HSPB8	36	RRAGD	61	FN1	86	CCL28
12	MMP8	37	WWC2	62	MAST1	87	MSRB3
13	C20orf103	38	FIBIN	63	DDR2	88	LOC400643
14	MAOB	39	MEOX2	64	DUOX2	89	NRP2
15	UCHL1	40	CRYAB	65	IGFBP5	90	ULBP2
16	RERG	41	PDE1A	66	LCA5	91	EPB41L4B
17	GNB5	42	TNS1	67	SYNC	92	RBPMS2
18	PCDHA1,...,13 PCDHAC1,2	43	CLC	68	PRKD1	93	APBB2
19	F5	44	KCNE4	69	FAM78B	94	CSRP2
20	BEAN1	45	ENTPD5	70	CASP1	95	PI15
21	MARCO	46	GPA33	71	TRPS1	96	LOC100509130
22	FAM84A	47	CXCL14	72	PPAPDC1A	97	LOC220930
23	CLDN11	48	CILP	73	TFPI	98	HIP1
24	KLHL21	49	DACT3	74	DES	99	DES FAM48A
25	GPX3	50	ZC3H12A	75	SMPX	100	LTBP3

Table 15: Top Colon Cancer Pathways Using CMNC-OBF Gene Set

Rank	Pathway	P.W. size	#Found	P-value
1	Cadherin sig. P.W.	158	63	$1.83E-20$
2	Wnt sig. P.W.	311	78	$7.25E-14$
3	Plasminogen activating cas.	18	9	$7.67E-05$
4	Alzheimer disease-presenilin P.W.	124	27	$1.02E-04$
5	CCKR sig. map	173	31	$8.84E-04$
6	Integrin signalling P.W.	192	32	$2.27E-03$
7	Blood coagulation	47	12	$2.32E-03$
8	Cytoskeletal regulation by Rho GTPase	83	17	$3.32E-03$
9	Huntington disease	139	24	$4.92E-03$
10	5HT4 type receptor med. sig. P.W.	33	8	$1.54E-02$
11	Heterotrimeric G-prot. sig. P.W.-rod outer seg. phototrans.	40	9	$1.63E-02$
12	p53 P.W. by glucose deprivation	22	6	$2.04E-02$
13	P53 P.W. feedback loops 1	6	3	$2.05E-02$
14	Adrenaline and noradrenaline biosyn.	30	7	$2.70E-02$
15	Inflammation med. by chemokine and cytokine sig. P.W.	261	35	$3.16E-02$
16	p53 P.W. feedback loops 2	53	10	$3.38E-02$
17	Sulfate assimilation	3	2	$3.39E-02$
18	Beta2 adrenergic receptor sig. P.W.	46	9	$3.53E-02$
19	Beta1 adrenergic receptor sig. P.W.	46	9	$3.53E-02$
20	Nicotinic acetylcholine receptor sig. P.W.	101	16	$3.70E-02$

Table 16: Top Colon Cancer Pathways Using REMAIN Gene Set

Rank	Pathway	P.W. size	#Found	P-value
1	Cadherin sig. P.W.	158	33	$2.21E-10$
2	Plasminogen activating cas.	18	7	$7.95E-05$
3	Wnt sig. P.W.	311	35	$9.27E-05$
4	Blood coagulation	47	10	$3.65E-04$
5	MYO sig. P.W.	2	2	$5.67E-03$
6	Activin beta sig. P.W.	2	2	$5.67E-03$
7	ALP23B sig. P.W.	2	2	$5.67E-03$
8	Ionotropic glutamate receptor P.W.	50	8	$7.47E-03$
9	Cytoskeletal regulation by Rho GTPase	83	11	$7.50E-03$
10	Nicotinic acetylcholine receptor sig. P.W.	101	12	$1.20E-02$
11	BMP/activin sig. P.W.-drosophila	4	2	$2.11E-02$
12	Heterotrimeric G-prot. sig. P.W.-Gi alpha & Gs alpha med. P.W.	164	16	$2.28E-02$
13	Synaptic vesicle trafficking	29	5	$2.37E-02$
14	Beta2 adrenergic receptor sig. P.W.	46	6	$4.47E-02$
15	Beta1 adrenergic receptor sig. P.W.	46	6	$4.47E-02$
16	Alzheimer disease-presenilin P.W.	124	12	$4.65E-02$
17	GBB sig. P.W.	1	1	$5.38E-02$
18	Axon guidance med. by Slit/Robo	26	4	$5.79E-02$
19	Gonadotropin-releasing hormone receptor P.W.	235	19	$6.82E-02$
20	5HT3 type receptor med. sig. P.W.	17	3	$6.94E-02$

Table 17: Top Colon Cancer Pathways Using POFAC Gene Set

Rank	Pathway	P.W. size	#Found	P-value
1	Ionotropic glutamate receptor P.W.	50	15	$1.72E-05$
2	Metabotropic glutamate receptor group III P.W.	69	12	$1.04E-02$
3	Nicotinic acetylcholine receptor sig. P.W.	101	15	$1.70E-02$
4	EGF receptor sig. P.W.	139	19	$1.75E-02$
5	Synaptic vesicle trafficking	29	6	$2.97E-02$
6	Plasminogen activating cas.	18	4	$5.66E-02$
7	Asparagine and aspartate biosyn.	5	2	$6.05E-02$
8	Nicotine pharmacodynamics P.W.	35	6	$6.25E-02$
9	Heterotrimeric G-prot. sig. P.W.-Gi alpha & Gs alpha med. P.W.	164	19	$6.86E-02$
10	Cytoskeletal regulation by Rho GTPase	83	11	$7.06E-02$
11	Beta1 adrenergic receptor sig. P.W.	46	7	$7.67E-02$
12	Gamma-aminobutyric acid synthesis	6	2	$8.28E-02$
13	Blood coagulation	47	7	$8.36E-02$
14	Huntington disease	139	16	$9.24E-02$
15	Interleukin sig. P.W.	98	12	$9.50E-02$
16	Coenzyme A biosyn.	7	2	$1.07E-01$
17	Heterotrimeric G-prot. sig. P.W.-Gq alpha & Go alpha med. P.W.	123	14	$1.17E-01$
18	Toll P.W.-drosophila	2	1	$1.46E-01$
19	Tyrosine biosyn.	2	1	$1.46E-01$
20	Pyridoxal phosphate salvage P.W.	2	1	$1.46E-01$
21	Histidine biosyn.	2	1	$1.46E-01$
22	Carnitine metabolism	2	1	$1.46E-01$
23	Coenzyme A linked carnitine metabolism	2	1	$1.46E-01$

Table 18: Top Colon Cancer Pathways Using SPM Gene Set

Rank	Pathway	P.W. size	#Found	P-value
1	Ionotropic glutamate receptor P.W.	50	11	$2.98E-03$
2	Heterotrimeric G-prot. sig. P.W.-Gi alpha & Gs alpha med. P.W.	164	24	$4.73E-03$
3	Axon guidance med. by Slit/Robo	26	7	$5.79E-03$
4	Heterotrimeric G-prot. sig. P.W.-Gq alpha & Go alpha med. P.W.	123	18	$1.31E-02$
5	Blood coagulation	47	8	$3.95E-02$
6	Cytoskeletal regulation by Rho GTPase	83	12	$4.05E-02$
7	Endogenous cannabinoid sig.	24	5	$4.70E-02$
8	Metabotropic glutamate receptor group III P.W.	69	10	$5.70E-02$
9	Plasminogen activating cas.	18	4	$5.97E-02$
10	Asparagine and aspartate biosyn.	5	2	$6.24E-02$
11	Nicotinic acetylcholine receptor sig. P.W.	101	13	$7.07E-02$
12	Beta1 adrenergic receptor sig. P.W.	46	7	$8.26E-02$
13	P53 P.W. feedback loops 1	6	2	$8.54E-02$
14	Gamma-aminobutyric acid synthesis	6	2	$8.54E-02$
15	Coenzyme A biosyn.	7	2	$1.10E-01$
16	Alpha adrenergic receptor sig. P.W.	25	4	$1.46E-01$
17	Toll P.W.-drosophila	2	1	$1.49E-01$
18	Tyrosine biosyn.	2	1	$1.49E-01$
19	Pyridoxal phosphate salvage P.W.	2	1	$1.49E-01$
20	Histidine biosyn.	2	1	$1.49E-01$

Table 19: Top Colon Cancer Pathways Using Top 2000 limma Gene Set

Rank	Pathway	P.W. size	#Found	P-value
1	Cadherin sig. P.W.	158	57	$4.79E-10$
2	Wnt sig. P.W.	311	69	$3.70E-06$
3	Integrin sig. P.W.	192	41	$2.68E-04$
4	Alzheimer disease-presenilin P.W.	124	26	$5.49E-04$
5	Gonadotropin-releasing hormone receptor P.W.	235	40	$6.16E-04$
6	Cytoskeletal regulation by Rho GTPase	83	19	$2.45E-03$
7	Nicotinic acetylcholine receptor sig. P.W.	101	20	$6.56E-03$
8	CCKR sig. map	173	28	$6.82E-03$
9	Blood coagulation	47	11	$8.66E-03$
10	Plasminogen activating cascade	18	6	$2.18E-02$
11	Nicotine pharmacodynamics P.W.	35	8	$2.54E-02$
12	Axon guidance mediated by semaphorins	23	6	$3.17E-02$
13	Huntington disease	139	21	$3.44E-02$
14	Sulfate assimilation	3	2	$4.57E-02$
15	Metabotropic glutamate receptor group II P.W.	48	9	$4.91E-02$
16	Muscarinic acetylcholine receptor 2 and 4 sig. P.W.	64	11	$4.96E-02$
17	p53 P.W.	88	14	$5.44E-02$
18	Hedgehog sig. P.W.	21	5	$6.31E-02$
19	Dopamine receptor mediated sig. P.W.	59	10	$7.27E-02$
20	Adrenaline and noradrenaline biosynthesis	30	6	$8.02E-02$

Table 20: Top Colon Cancer Pathways Using Top 2000 limma Gene Set

Rank	Pathway	P.W. size	#Found	P-value
1	Cadherin sig. P.W.	158	17	$1.04E-12$
2	Wnt sig. P.W.	311	18	$4.35E-09$
3	VEGF sig. P.W.	72	4	$6.46E-03$
4	Muscarinic acetylcholine receptor 2 and 4 sig. P.W.	64	3	$2.78E-02$
5	Angiogenesis	176	5	$3.43E-02$
6	Alzheimer disease-presenilin P.W.	124	4	$3.81E-02$
7	Angiotensin II-stimulated sig. through G proteins and beta-arrestin	39	2	$5.99E-02$
8	Blood coagulation	47	2	$8.26E-02$
9	Nicotinic acetylcholine receptor sig. P.W.	101	3	$8.38E-02$
10	Gonadotropin-releasing hormone receptor P.W.	235	5	$9.17E-02$
11	PI3 kinase P.W.	55	2	$1.07E-01$
12	Dopamine receptor mediated sig. P.W.	59	2	$1.21E-01$
13	Salvage pyrimidine ribonucleotides	13	1	$1.23E-01$
14	Muscarinic acetylcholine receptor 1 and 3 sig. P.W.	60	2	$1.24E-01$
15	Plasminogen activating cascade	18	1	$1.66E-01$
16	EGF receptor sig. P.W.	139	3	$1.67E-01$
17	5-Hydroxytryptamine degredation	21	1	$1.91E-01$
18	PDGF sig. P.W.	149	3	$1.92E-01$
19	p53 P.W. by glucose deprivation	22	1	$1.99E-01$
20	Cytoskeletal regulation by Rho GTPase	83	2	$2.05E-01$

Table 21: Top Genes of AML Using REMAIN

Rank	Gene	Rank	Gene	Rank	Gene	Rank	Gene
1	LTF	26	SYNE1	51	COL17A1	76	MMP8
2	CRISP3	27	PRDM5	52	CAST	77	IVNS1ABP
3	ORM1 ORM2	28	BASP1	53	FGR	78	CYBB
4	CHIT1	29	DEFA4	54	CEACAM1	79	HOMER3
5	DNAH10	30	PGLYRP1	55	RAB31	80	FPR1
6	ORM1	31	OLFM4	56	TTPAL	81	SPINT2
7	S100A9	32	CHI3L1	57	KDELR1	82	SCPEP1
8	S100P	33	HBA1 HBA2	58	CD99	83	NAV2
9	DEFA1 DEFA1B DEFA3	34	VEPH1	59	APOC2	84	HNRPLL
10	LCN2	35	STOM	60	CDA	85	CD59
11	MMP9	36	TCN1	61	HK3	86	ALOX5
12	HBB	37	MS4A3	62	ARG1	87	CYP4F2 CYP4F3
13	TFF3	38	TUBA4A	63	GLT1D1	88	FLT3
14	CYP4F3	39	APP	64	TCL1A	89	HP
15	CAMP	40	ITGAM	65	CKAP4	90	NCF2
16	CRISP2	41	ATP2C2	66	CEACAM6	91	CT45A1,2,3,4,5,6 LOC100133581
17	WT1	42	UGCG	67	ALOX5AP	92	ANXA5
18	DEFT1P DEFT1P2	43	PLBD1	68	FCN1	93	CLC
19	CEACAM8	44	HSPC159	69	C5orf32	94	KLF5
20	CD24	45	CLEC4D	70	RBP7	95	ANXA3
21	CSTA	46	CCND3	71	BCL2L15	96	HIST1H2BK
22	S100A12	47	PPEF1	72	CXCR1	97	SLC25A21
23	S100A8	48	LGALS3	73	PKP2	98	LOC404266
24	BPI	49	NGFRAP1	74	RETN	99	NPW
25	CD177	50	ABCA13	75	S1PR5	100	GLUD1

Table 22: Top Genes of AML Using CMNC-OBF

Rank	Gene	Rank	Gene	Rank	Gene	Rank	Gene
1	ORM1 ORM2	26	CD177	51	S100A8	76	RETN
2	LTF	27	ATP2C2	52	CXCR1	77	AMPD3
3	CRISP3	28	S100A12	53	FPR1	78	TCL1A
4	CHIT1	29	TCN1	54	MMP8	79	CCND3
5	DNAH10	30	VEPH1	55	ARG1	80	FGR
6	CAMP	31	GLT1D1	56	C5orf32	81	FCN1
7	LCN2	32	COL17A1	57	PKP2	82	CDA
8	MMP9	33	DEFA4	58	BPI	83	PPEF1
9	ORM1	34	HSPC159	59	SLC25A21	84	ITGAM
10	CYP4F3	35	CEACAM1	60	CD99	85	GJB6
11	CRISP2	36	RBP7	61	FLT3	86	PFKP
12	TFF3	37	CD24	62	ANXA3	87	CA4
13	DEFA1 DEFA1B DEFA3	38	S100A9	63	PLBD1	88	APP
14	CHI3L1	39	CLEC4D	64	RAB31	89	MBOAT2
15	WT1	40	HP	65	OLR1	90	CD59
16	OLFM4	41	IL18RAP	66	KLF5	91	AKTIP
17	SYNE1	42	KDELR1	67	LGALS3	92	MS4A3
18	DEFT1P DEFT1P2	43	STOM	68	CYP4F2 CYP4F3	93	WIPI1
19	PGLYRP1	44	BASP1	69	PADI4	94	HNRPLL
20	S100P	45	HP HPR	70	CTSE	95	CPNE3
21	PRDM5	46	ABCA13	71	SLPI	96	NAV2
22	HBB	47	CKAP4	72	QPCT	97	CSTA
23	UGCG	48	TTPAL	73	HBA1 HBA2	98	HK3
24	TUBA4A	49	CEACAM6	74	PECR	99	CYBB
25	CEACAM8	50	BCL2L15	75	CDC14B	100	KIF21B

Table 23: Top Genes of AML Using POFAC

Rank	Gene	Rank	Gene	Rank	Gene	Rank	Gene
1	S100A12	26	UGCG	51	MMP8	76	CD59
2	S100A9	27	OLFM4	52	SDHC	77	PKP2
3	ORM1 ORM2	28	DEFT1P DEFT1P2	53	ATP2C2	78	LGALS3
4	CRISP3	29	ABCA13	54	TMEM56	79	CXCR1
5	LTF	30	GLT1D1	55	CTSE	80	IL18RAP
6	CAMP	31	CDA	56	HIST1H2BK	81	PADI4
7	LCN2	32	HP HPR	57	RAB31	82	ELANE
8	DNAH10	33	ANXA3	58	FCN1	83	KLF5
9	CHIT1	34	HP	59	CLEC4D	84	CYBB
10	MMP9	35	TUBA4A	60	HBB	85	CD99
11	CYP4F3	36	DEFA4	61	SLPI	86	BCL2L15
12	S100A8	37	FGR	62	KDELR1	87	MBOAT2
13	ORM1	38	FPR1	63	FLT3	88	B4GALT5
14	CHI3L1	39	CKAP4	64	SLC25A21	89	GCA
15	CEACAM8	40	CEACAM1	65	ARG1	90	ADSS
16	TFF3	41	CD24	66	ALOX5AP	91	LRRN4
17	CRISP2	42	RBP7	67	STOM	92	C5AR1
18	PGLYRP1	43	CD177	68	HSPC159	93	CYP4F2 CYP4F3
19	CEACAM6	44	QPCT	69	GLUD1	94	GJB6
20	WT1	45	COL17A1	70	NCF2	95	ALOX5
21	DEFA1 DEFA1B DEFA3	46	BPI	71	C5orf32	96	TTPAL
22	TCN1	47	ITGAM	72	C19orf59	97	TGFBI
23	SYNE1	48	HK3	73	VEPH1	98	AKTIP
24	PLBD1	49	MS4A3	74	BASP1	99	PKLR
25	S100P	50	PRDM5	75	TACSTD2	100	GLRX

Table 24: Top Genes of AML Using limma

Rank	Gene	Rank	Gene	Rank	Gene	Rank	Gene
1	CRISP3	26	CKAP4	51	TTPAL	76	BPI
2	CAMP	27	CEACAM6	52	CDA	77	GCLM
3	MMP9	28	CD24	53	DEFA4	78	TUBB1
4	LCN2	29	TFF3	54	MBOAT2	79	PCOLCE2
5	ORM1	30	S100A12	55	CD59	80	HK3
6	CRISP3	31	FPR1	56	CDC14B	81	RCN1
7	CAMP	32	C5orf32	57	RGL4	82	GYG1
8	MMP9	33	CEACAM1	58	CYBB	83	OLR1
9	LCN2	34	DNAH10	59	FLT3	84	APOC2
10	ORM1	35	ARG1	60	PKP2	85	CRISP2
11	CHI3L1	36	CEACAM8	61	RAB31	86	ALOX5AP
12	WT1	37	ANXA3	62	C19orf59	87	GCA
13	CYP4F3	38	SYNE1	63	KLF5	88	NFIA
14	PGLYRP1	39	HSPC159	64	GGTA1	89	HNRPLL
15	UGCG	40	CHIT1	65	DEFT1P DEFT1P2	90	CD99
16	OLFM4	41	MMP8	66	WIPI1	91	ALOX5
17	GLT1D1	42	QPCT	67	BASP1	92	CCND3
18	TCN1	43	LTF	68	MAMDC2	93	TMEM56
19	ORM1 ORM2	44	SLPI	69	LRIG1	94	EPC1
20	TUBA4A	45	STOM	70	ITGAM	95	PKLR
21	HP	46	PLBD1	71	TACSTD2	96	ABCA13
22	CLEC4D	47	PADI4	72	KIF21B	97	S100P
23	RBP7	48	ATP2C2	73	BCL2L15	98	GLRX
24	IL18RAP	49	COL17A1	74	PFKP	99	PECR
25	HP HPR	50	RETN	75	AMPD3	100	FCAR

Table 25: Top AML Pathways Using CMNC-OBF Gene Set

Rank	Pathway	P.W. size	#Found	P-value
1	Heme biosyn.	13	7	$2.78E-04$
2	Pentose phosphate P.W.	8	4	$7.34E-03$
3	De novo purine biosyn.	30	8	$8.40E-03$
4	Interferon-gamma sig. P.W.	30	8	$8.40E-03$
5	N-acetylglucosamine metabolism	7	3	$2.92E-02$
6	Thyrotropin-releasing hormone receptor sig. P.W.	60	11	$2.92E-02$
7	Blood coagulation	47	9	$3.64E-02$
8	Inflammation med. by chemokine and cytokine sig. P.W.	261	34	$3.94E-02$
9	Formyltetrahydroformate biosyn.	8	3	$4.07E-02$
10	VEGF sig. P.W.	72	12	$4.32E-02$
11	Oxytocin receptor med. sig. P.W.	58	10	$5.10E-02$
12	5HT2 type receptor med. sig. P.W.	67	11	$5.58E-02$
13	Dopamine receptor med. sig. P.W.	59	10	$5.59E-02$
14	Glycolysis	22	5	$5.89E-02$
15	CCKR sig. map	173	23	$6.59E-02$
16	Beta2 adrenergic receptor sig. P.W.	46	8	$7.25E-02$
17	Beta1 adrenergic receptor sig. P.W.	46	8	$7.25E-02$
18	5HT1 type receptor med. sig. P.W.	46	8	$7.25E-02$
19	Angiotensin II-stim. sig. through G prot.s and beta-arrestin	39	7	$7.86E-02$
20	Tetrahydrofolate biosyn.	5	2	$8.12E-02$

Table 26: Top AML Pathways Using REMAIN Gene Set

Rank	Pathway	P.W. size	#Found	P-value
1	Interferon-gamma sig. P.W.	30	7	$5.86E-04$
2	Alzheimer disease-presenilin P.W.	124	13	$6.11E-03$
3	Inflammation med. by chemokine and cytokine sig. P.W.	261	22	$6.22E-03$
4	Glutamine glutamate conversion	4	2	$1.52E-02$
5	Heme biosyn.	13	3	$2.33E-02$
6	Ras Pathway	76	8	$2.72E-02$
7	Gonadotropin-releasing hormone receptor P.W.	235	18	$2.87E-02$
8	Heterotrimeric G-prot. sig. P.W.-Gq alpha & Go alpha med. P.W.	123	11	$3.08E-02$
9	Mannose metabolism	6	2	$3.21E-02$
10	Heterotrimeric G-prot. sig. P.W.-rod outer seg. phototrans.	40	5	$4.01E-02$
11	Oxidative stress response	56	6	$4.83E-02$
12	B cell activation	72	7	$5.29E-02$
13	Integrin signalling P.W.	192	14	$6.75E-02$
14	Insulin/IGF P.W.-mitogen act. prot. kinase/MAP kinase cas.	33	4	$6.90E-02$
15	Blood coagulation	47	5	$6.97E-02$
16	TCA cycle	10	2	$7.92E-02$
17	Heterotrimeric G-prot. sig. P.W.-Gi alpha & Gs alpha med. P.W.	164	12	$8.40E-02$
18	Pyridoxal phosphate salvage P.W.	2	1	$8.84E-02$
19	Alanine biosyn.	2	1	$8.84E-02$
20	Interleukin sig. P.W.	98	8	$8.92E-02$

Table 27: Top AML Pathways Using POFAC Gene Set

Rank	Pathway	P.W. size	#Found	P-value
1	Pentose phosphate P.W.	8	5	$1.15E-03$
2	Formyltetrahydroformate biosyn.	8	5	$1.15E-03$
3	Heme biosyn.	13	6	$1.77E-03$
4	Inflammation med. by chemokine and cytokine sig. P.W.	261	41	$1.80E-03$
5	De novo pyrimidine deoxyribonucleotide biosyn.	13	5	$8.84E-03$
6	Tetrahydrofolate biosyn.	5	3	$1.27E-02$
7	Angiotensin II-stim. sig. through G prot.s and beta-arrestin	39	9	$1.41E-02$
8	Ras Pathway	76	14	$1.67E-02$
9	p38 MAPK P.W.	42	9	$2.15E-02$
10	T cell activation	96	16	$2.51E-02$
11	Interferon-gamma sig. P.W.	30	7	$2.70E-02$
12	Insulin/IGF P.W.-mitogen act. prot. kinase/MAP kinase cas.	33	7	$4.15E-02$
13	Integrin signalling P.W.	192	26	$5.21E-02$
14	Alzheimer disease-presenilin P.W.	124	18	$5.64E-02$
15	Salvage pyrimidine deoxyribonucleotides	4	2	$5.67E-02$
16	Glutamine glutamate conversion	4	2	$5.67E-02$
17	Cytoskeletal regulation by Rho GTPase	83	13	$5.98E-02$
18	Glycolysis	22	5	$6.20E-02$
19	Cell cycle	22	5	$6.20E-02$
20	De novo purine biosyn.	30	6	$7.07E-02$

Table 28: Top AML Pathways Using SPM Gene Set

Rank	Pathway	P.W. size	#Found	P-value
1	Ubiquitin proteasome P.W.	66	43	$1.72E-08$
2	T cell activation	96	46	$2.07E-05$
3	Inflammation med. by chemokine and cytokine sig. P.W.	261	98	$3.00E-05$
4	Apoptosis sig. P.W.	122	52	$1.21E-04$
5	p53 P.W.	88	40	$1.98E-04$
6	PDGF sig. P.W.	149	59	$2.92E-04$
7	B cell activation	72	34	$2.94E-04$
8	Heme biosyn.	13	11	$4.44E-04$
9	Cell cycle	22	15	$4.45E-04$
10	CCKR sig. map	173	65	$5.77E-04$
11	Ras Pathway	76	33	$1.40E-03$
12	Transcription regulation by bZIP transcription factor	56	26	$1.76E-03$
13	Toll receptor sig. P.W.	60	26	$4.30E-03$
14	DNA replication	35	17	$6.59E-03$
15	Interleukin sig. P.W.	98	37	$7.18E-03$
16	De novo purine biosyn.	30	15	$8.03E-03$
17	p53 P.W. feedback loops 2	53	22	$1.27E-02$
18	Parkinson disease	100	36	$1.53E-02$
19	Interferon-gamma sig. P.W.	30	14	$1.74E-02$
20	VEGF sig. P.W.	72	27	$2.06E-02$

Table 29: Top AML Pathways Using Top 2000 limma Gene Set

Rank	Pathway	P.W. size	#Found	P-value
1	Heme biosynthesis	13	7	$2.75E-04$
2	Angiotensin II-stimulated signaling through G proteins and beta-arrestin	39	10	$4.46E-03$
3	Inflammation mediated by chemokine and cytokine sig. P.W.	261	38	$6.49E-03$
4	Pentose phosphate P.W.	8	4	$7.30E-03$
5	Formyltetrahydroformate biosynthesis	8	4	$7.30E-03$
6	Interferon-gamma sig. P.W.	30	8	$8.33E-03$
7	Histamine H1 receptor mediated sig. P.W.	44	10	$9.90E-03$
8	Oxytocin receptor mediated sig. P.W.	58	12	$1.01E-02$
9	5HT2 type receptor mediated sig. P.W.	67	13	$1.24E-02$
10	Thyrotropin-releasing hormone receptor sig. P.W.	60	12	$1.28E-02$
11	Blood coagulation	47	10	$1.50E-02$
12	Glycolysis	22	6	$1.89E-02$
13	B cell activation	72	13	$2.09E-02$
14	De novo purine biosynthesis	30	7	$2.49E-02$
15	Ras P.W.	76	13	$3.04E-02$
16	Beta2 adrenergic receptor sig. P.W.	46	9	$3.22E-02$
17	Beta1 adrenergic receptor sig. P.W.	46	9	$3.22E-02$
18	5HT1 type receptor mediated sig. P.W.	46	9	$3.22E-02$
19	De novo pyrimidine deoxyribonucleotide biosynthesis	13	4	$3.55E-02$
20	Opioid prodynorphin P.W.	33	7	$3.85E-02$
